# Combined FAK and MEK inhibition suppresses chromosome 8 gain malignant peripheral nerve sheath tumors

**DOI:** 10.1172/JCI201331

**Published:** 2026-08-11

**Authors:** Guangfeng Wang, Dana C. Borcherding, Jiawan Wang, Xiaochun Zhang, Liuzhan Yang, Gorkem Oztosun, James J. Sears, Kangwen Xiao, Belinda B. Garana, Mark I. Zoberi, Aaron U. Bektas, Jeffrey J. Szymanski, Richa Rathore, Silvia Coma, Jonathan A. Pachter, Sara J.C. Gosline, Christine A. Pratilas, Angela C. Hirbe

**Affiliations:** 1 Division of Oncology, Department of Medicine, Washington University in St. Louis School of Medicine and Siteman Cancer Center, St. Louis, Missouri, USA.; 2 Department of Oncology, Sidney Kimmel Comprehensive Cancer Center and Johns Hopkins University School of Medicine, Baltimore, Maryland, USA.; 3 Division of Biological Sciences, Pacific Northwest National Laboratory, Seattle, Washington, USA.; 4 Department of Radiation Oncology, Mayo Clinic, Rochester, Minnesota, USA.; 5 Verastem Oncology, Needham, Massachusetts, USA.; 6 Department of Biomedical Engineering, Oregon Health and Sciences University, Portland, Oregon, USA.

**Keywords:** Cell biology, Oncology, Biomarkers, Cancer, Drug therapy

## Abstract

Aneuploidy is a hallmark of cancer often associated with inferior prognosis. Copy number gains of chromosome 8 (chr8) are recurrent in multiple cancers, including breast, prostate, and colorectal cancers, and sarcomas, such as malignant peripheral nerve sheath tumors (MPNSTs). MPNSTs are aggressive, hard-to-treat sarcomas frequently linked to the neurofibromatosis type 1 (NF1) cancer predisposition syndrome. To investigate the role of chr8 gain in MPNST pathogenesis, we performed a CRISPR-knockout screen and identified 58 essential genes on chr8, including *PTK2*, which encodes focal adhesion kinase (FAK). We evaluated FAK as a therapeutic target and tested small-molecule FAK inhibitors (FAKi) alone or combined with RAF/MEK inhibitors (RAF/MEKi), a class of agents relevant to NF1-deficient tumors with ERK pathway hyperactivation. Both pharmacological and genetic inhibition of FAK reduced MPNST cell proliferation in vitro and tumor growth in vivo. Combined FAKi and RAF/MEKi treatment further suppressed phosphorylation of FAK, STAT3, and AKT while increasing cleaved caspase-3 and poly (ADP-ribose) polymerase 1 (PARP-1), indicating enhanced apoptosis. In MPNST patient-derived xenograft (PDX) models, combination therapy significantly reduced tumor growth, showing superior efficacy, particularly in chr8 gain MPNST PDX. These results support FAK/RAF/MEK cotargeting as a promising therapeutic strategy for chr8 gain MPNST and related tumors.

## Abstract 2

Dual FAK and RAF/MEK inhibition overcomes resistance, suppresses growth, and induces tumor regression in chromosome 8 gain malignant peripheral nerve sheath tumors.

## Graphical abstract

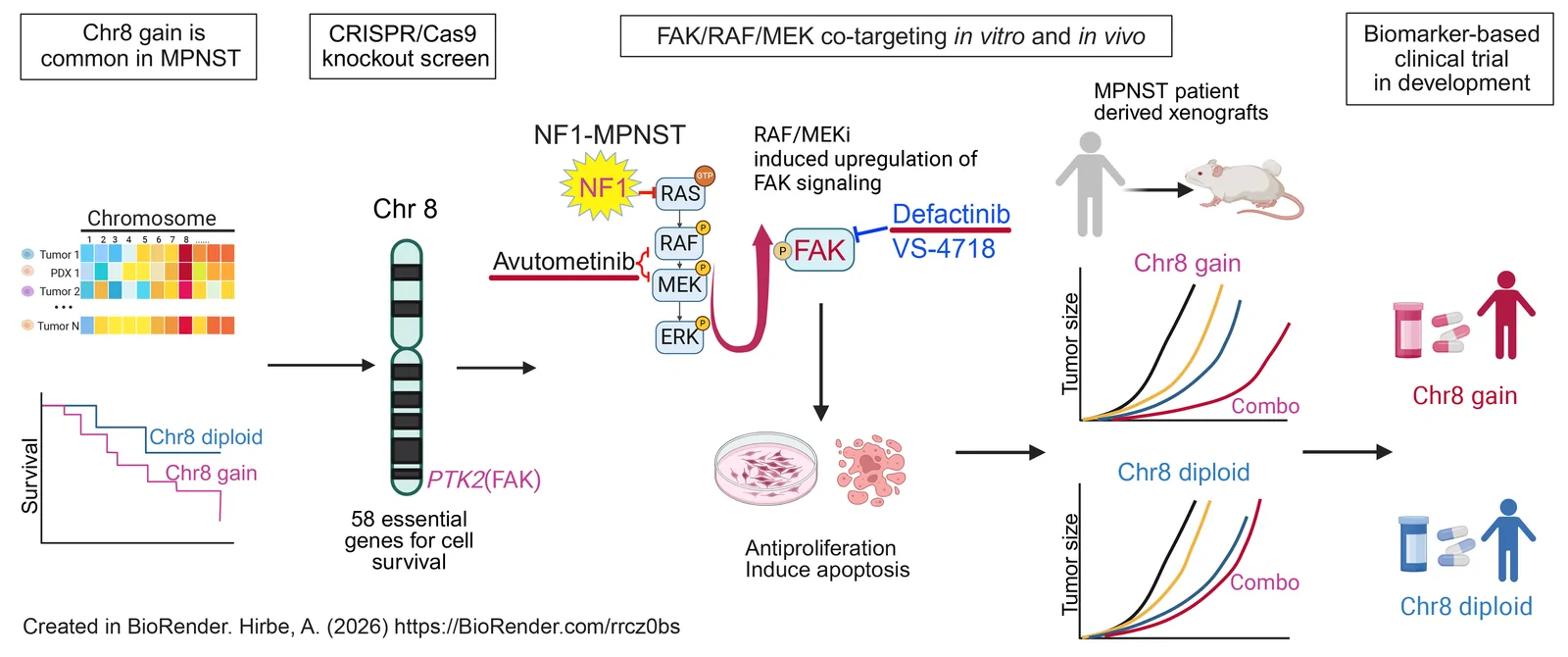

## Introduction

Malignant peripheral nerve sheath tumors (MPNSTs) are aggressive soft tissue sarcomas that arise from the protective sheaths surrounding peripheral nerves. Accounting for approximately 10% of all soft tissue sarcomas (1), MPNSTs present substantial diagnostic and therapeutic challenges because of their complex biology. These tumors can occur sporadically or in association with neurofibromatosis type 1 (NF1), a genetic disorder characterized by *NF1* germline mutations (2, 3). Surgical resection with negative margins remains the cornerstone of curative MPNST treatment, but achieving clear margins is challenging due to the tumor’s infiltrative nature (4). Radiation therapy is frequently used in conjunction with surgery to enhance local control and may serve as a primary treatment for inoperable tumors (5). Despite aggressive therapy, 50% of patients recur with metastatic disease, which is universally fatal. Chemotherapy has shown limited efficacy in MPNSTs (6, 7), prompting the investigation of alternative therapeutic approaches, including targeted therapies (8) and immunotherapy (9), though effective treatment options remain an unmet need.

The majority of MPNSTs, whether sporadic or NF1 associated, harbor loss-of-function *NF1* mutations, leading to dysfunction of neurofibromin, a tumor suppressor (GTPase-activating protein) that negatively regulates the RAS/MAPK signaling pathway (10). *NF1* loss results in hyperactivation of ERK signaling, driving uncontrolled cell proliferation and tumor growth, making it an important target for therapeutic intervention (11, 12). MEK1 and -2 (MAPK/ERK kinases 1 and 2) are key components of this pathway, acting downstream of RAS and RAF to activate ERK1 and -2 signaling. In light of the observed MEK/ERK hyperactivation in MPNST, MEK has emerged as a promising therapeutic target. However, MEK inhibitors (MEKi) as single agents have shown insufficient efficacy in preclinical MPNST studies (13–18). The inadequate effects of MEKi alone are likely due to additional alterations in genes such as *TP53*, *EED*, *SUZ12*, and *CDKN2A* (19, 20), chromosomal alterations such as chromosome 8 (chr8) gain (21), and adaptive dysregulation of parallel signaling pathways. Prior work in our lab identified chr8 gain as the most common copy number alteration in MPNST (22, 23). This chromosomal aberration has also been shown to be a driver in other solid tumors (24–27).

In this study, we identified chr8 genes essential for MPNST survival using a CRISPR guide RNA (gRNA) screen. *PTK2*, encoding focal adhesion kinase (FAK), was one such gene and was found to be highly expressed in MPNST. Genetic knockdown of *PTK2* reduced MPNST cell proliferation in vitro and tumor growth in vivo. We tested the effects of combined FAKi with MEKi using avutometinib, a MEKi with a novel mechanism of action in that it stabilizes a RAF/MEK complex such that MEK cannot be phosphorylated (i.e., “RAF/MEK clamp”) (28, 29). When the FAKi defactinib was combined with avutometinib, additive or synergistic effects enhancing cell death were observed. In chr8 gain MPNST patient-derived xenograft (PDX) models, FAK inhibition moderately reduced tumor growth, but combination therapy with the RAF/MEKi avutometinib proved to be more effective, resulting in tumor regressions in PDX models. These findings support a promising therapeutic strategy for this aggressive sarcoma, which could be translated to other solid tumors with chr8 gain.

## Results

### CRISPR screen identifies 58 genes on chr8 important for MPNST cell survival.

Given the frequent gain of chr8 in MPNST and its potential role in driving tumor progression, we sought to systematically investigate genes on this chromosome to uncover potentially novel biomarkers and therapeutic vulnerabilities. Using a CRISPR gRNA screen targeting all 652 genes on chr8, we identified 58 genes (*P* < 0.05) crucial for the survival of human MPNST JH-2-002 cells (Figure 1A and Supplemental Table 1; supplemental material available online with this article; https://doi.org/10.1172/JCI201331DS1). To further prioritize clinically relevant targets, we applied the LinkedOmics Target Explorer (https://targets.linkedomics.org/), which highlighted 5 genes — *ATP6V1B2*, *FGFR1*, *POLB*, *PTK2*, and *TOP1MT* — as druggable candidates. These genes were therefore selected for deeper investigation as potential pathological biomarkers and therapeutic targets in MPNST.

To evaluate the clinical relevance of these candidate genes, we queried The Cancer Genome Atlas (TCGA) Firehose Legacy Sarcoma dataset using the cBioPortal (http://cbioportal.org). Among 243 tumors with available mutation and copy number alteration (CNA) data, 7 samples were excluded due to mutations in any of the 5 druggable targets, resulting in a final cohort of 236 samples for subsequent analysis. Notably, 4 of the 5 genes (*FGFR1*, *POLB*, *PTK2*, and *TOP1MT*) exhibited recurrent copy number amplifications, whereas *ATP6V1B2* exhibited no amplification in this cohort and was therefore excluded from survival analyses. Survival analysis revealed that amplification of *PTK2* and *TOP1MT* was significantly associated with worse overall survival compared with patients without such amplification (Figure 1, B–D). Specifically, *PTK2* amplification showed the strongest impact, correlating with inferior overall survival (*P* = 3.097 × 10^–3^) over a 120-month evaluation period (Figure 1C). *TOP1MT* encodes a mitochondrial topoisomerase involved in regulating mitochondrial function and cellular metabolism (30, 31). Although *TOP1MT* may contribute to tumor biology, it is less directly linked to canonical oncogenic signaling pathways and currently lacks well-established clinically actionable inhibitors. In contrast, *PTK2* encodes FAK, a key mediator of oncogenic signaling involved in tumor proliferation, survival, migration, and therapeutic resistance (32). Collectively, these findings imply *PTK2* may play a more direct role in sarcoma progression and highlight its possibility as a therapeutic target in MPNST. As TCGA sarcoma cohort includes a variety of sarcoma subtypes, the observed frequency of chr8 amplification, including *PTK2* amplification, is expected to be lower than in MPNST-focused datasets.

We next performed Western blotting assays to evaluate FAK protein expression in MPNST patient tumors, and the results demonstrated increased FAK expression in MPNST tissues compared with plexiform neurofibroma (pNF) tissues (Figure 1E). To further investigate its relevance in MPNST, we analyzed *PTK2* expression in MPNST patient samples (*n* = 94) based on variance-stabilizing transformation–normalized (VST-normalized) RNA-seq data (21). The results demonstrated that *PTK2* gene expression was significantly lower in the chr8 diploid MPNST group (*n* = 45) compared with the chr8 gain group (*n* = 49) (*P* = 0.0202, Figure 1F). All patient samples included in the analysis exhibited *PTK2* expression levels greater than 4. In addition, survival analysis of MPNST patients from the JHU cohort, GEM, and GSE241224 (*n* = 132) demonstrated that patients with high *PTK2* expression tended to have poorer survival (Figure 1G). This finding is consistent with the CRISPR screening data, suggesting that elevated *PTK2* expression contributes to chr8 gain effects in MPNST. The observed upregulation of *PTK2* in chr8 gain tumors supports its potential role in the pathogenesis of this aggressive tumor.

### Knockdown of PTK2 inhibits MPNST cell proliferation and tumor growth.

To investigate the involvement of *PTK2* (FAK) in the pathogenesis of MPNSTs, we utilized lentiviral delivery of shRNA to achieve effective knockdown of *PTK2* expression in both murine and human MPNST cell lines (Figure 2, A and B). In vitro Incucyte live-cell experiments revealed that *PTK2* knockdown significantly impaired cell proliferation in the murine MPNST cell line JW23.3, as well as in the human MPNST cell lines JH-2-002 and WU-356 (Figure 2C). Representative images of cell confluences and morphology are shown in Figure 2D and Supplemental Figure 1. These findings illustrate the role of *PTK2* in sustaining the growth and survival of MPNST cells, further suggesting its potential as a key oncogenic driver in this malignancy. An in vivo study using murine JW23.3 cells in an immune-competent model further demonstrated the oncogenic significance of *PTK2*. *PTK2* knockdown in these cells led to markedly reduced tumor growth to approximately 50% of shLacZ controls, indicating an important function for *PTK2* in promoting tumorigenesis and progression in MPNST (Supplemental Figure 2). For the human JH-2-002 cell line, cells were injected orthotopically to the sciatic nerve of nude mice. In these mice, *PTK2* knockdown increased overall survival by nearly 2 weeks (Figure 2E and Supplemental Figure 3). H&E staining shows MPNST morphology with high cellular proliferation with marked nuclear atypia and mitoses. In IHC staining, FAK expression was substantially reduced in shPTK2 tumors. Ki67 staining demonstrated a decrease in the proliferation, confirming effective target suppression and supporting the role of PTK2/FAK signaling in MPNST tumor growth (Figure 2, F and G). This in vivo evidence reinforces the notion that *PTK2* is important not only for cell proliferation but also for tumor growth and development in MPNST.

### FAK inhibition leads to decreased proliferation and increased cell death.

Next, we used the FAKi defactinib to assess the effects of pharmacological FAK inhibition on cell proliferation and cell death in both murine and human MPNST cell lines. Defactinib (VS-6063) is a potent and selective FAKi that has advanced to late-stage clinical development (33). In combination with the RAF/MEK “clamp” avutometinib, defactinib received FDA accelerated approval for the treatment of patients with KRAS-mutated, low-grade serous ovarian cancer based on data from the phase II study RAMP 201 (34). The FAKi defactinib demonstrated significant antiproliferative activity against MPNST cell lines (Figure 3A). The IC_50_ values for defactinib treatments ranged from 1.70 μM to 2.03 μM in murine MPNST JW23.3 cells and human MPNST JH-2-002 and WU-356 cells (Supplemental Table 2). In addition, defactinib increased cell death by 3- to 6-fold in JW23.3, JH-2-002, and WU-356 cells (Figure 3B). In contrast, for the chr8 diploid cell line JH-2-055-d, defactinib exhibited much higher IC_50_ values (>10 μM) (Supplemental Table 2). The potent inhibitory effects of defactinib on chr8 gain MPNST cells reinforces the importance of FAK as a critical therapeutic target in MPNST.

To investigate the mechanism of action of defactinib, biomarkers for crucial survival pathways were analyzed using the WES Simple Western assay. As expected, FAK inhibition by defactinib effectively reduced the phosphorylation of FAK itself in a dose- and time-dependent manner (Figure 3C). Defactinib treatment also suppressed the activation of downstream AKT, STAT3, and ERK signaling while increasing cleavage of the apoptotic proteins caspase-3 and poly (ADP-ribose) polymerase 1 (PARP-1) (Figure 3D). Thus, these data demonstrate that FAK inhibition decreased cell proliferation and induced apoptosis in MPNST cells.

### Synergistic effect of the combination of FAKi and RAF/MEKi.

The antiproliferative effect of the FAKi defactinib was further enhanced when combined with avutometinib, a RAF/MEK clamp inhibitor (Figure 4 and Supplemental Figures 4 and 5). The defactinib/avutometinib combination synergistically reduced proliferation with statistically significant synergy scores averaging between 11.71 and 17.69 for NF1-MPNST cell lines (JW23.3, JH-2-002, and WU-356) (Figure 4A). A synergy score greater than 10 is indicative of synergy. The cell confluence over time demonstrated that RAF/MEK inhibition initially promoted an increase in confluence, followed by a slowing of proliferation. Notably, the combination of RAF/MEKi and FAKi began to show superior efficacy compared with either single agent alone from approximately 48 hours onward, indicating an enhanced and sustained inhibitory effect on tumor cell growth (Figure 4B). Representative images for MPNST cells treated with avutometinib and/or defactinib are shown in Figure 4C and Supplemental Figure 5. In the chr8 diploid cell line JH-2-055-d, the synergy score was 6.39 (Supplemental Figure 6), indicating slight additive effects. While the doses shown here are higher than those utilized for serous ovarian cancer, MPNSTs are a more aggressive entity, and the doses shown are in line with the IC_50_s for these cells. Further, additive effects at a range of lower doses are observed as well (Supplemental Figure 7). These data suggest that cotargeting the FAK and RAF/MEK pathways effectively improves the efficacy in chr8 gain MPNST cells, which are notoriously resistant to single-agent therapies.

### Synergy of FAKi and RAF/MEKi to induce cell death.

To further evaluate the potential of this combination strategy and the mechanisms of synergy, we investigated the effect of the combination on cell death, relevant survival signaling pathways, and apoptotic pathway markers. The combination of defactinib and avutometinib synergistically induced cell death in JW23.3, JH-2-002, and WU-356 cells (synergy scores 19.8–45.2), as determined by the Incucyte cell death assay (Figure 5, A and B). Consistently, representative images (Figure 4C and Supplemental Figure 5) using YOYO-1 green fluorescent dye showed that the combination treatment resulted in an increased number of dead cells, characterized by bright green nuclear fluorescence and cell shrinkage.

As expected, incubation with avutometinib effectively suppressed the phosphorylation of downstream ERK (Figure 5C and Supplemental Figures 7 and 8). Importantly, consistent with previous findings (35), MEKi-treated MPNST cells exhibited upregulation of FAK-related genes (Supplemental Figure 8A) (17, 36), and MEK inhibition with avutometinib alone triggered a compensatory activation of FAK and AKT (Supplemental Figure 8B and Figure 5C), which can contribute to therapeutic resistance. This compensatory feedback loop underscores the limitation of MEKi monotherapy in MPNST, where tumor cells activate alternative survival pathways in response to treatment, and supports the rationale for combination strategies, such as incorporating FAK inhibition. Indeed, combining the FAKi defactinib with the RAF/MEKi avutometinib mitigated this compensatory action in JW23.3, JH-2-002, and WU-356 cells (Figure 5C and Supplemental Figure 7). The addition of a FAKi not only reduced FAK phosphorylation but also further downregulated the activity of key downstream proteins, including AKT, STAT3, and ERK (Figure 5C and Supplemental Figure 7). This broad inhibition of multiple survival pathways by the FAKi/RAF/MEKi combination substantially enhanced the antiproliferation response, as evidenced by increased cleavage of caspase-3 and PARP-1, biomarkers of apoptosis (Figure 5C and Supplemental Figure 7), supporting the hypothesis that dual inhibition of FAK and RAF/MEK signaling pathways potentiates cell death.

These data highlight the mechanistic rationale for combining a RAF/MEKi with a FAKi in MPNST. The dual-inhibition strategy successfully targets multiple compensatory signaling pathways, overcoming resistance mechanisms that limit the efficacy of single-agent treatments.

### FAKi and RAF/MEKi combination inhibits tumor growth in PDX mouse models.

The efficacy of the FAKi VS-4718, both as monotherapy and in combination with the RAF/MEKi avutometinib, was evaluated in PDX mouse models of MPNST with either chr8 gain (Figure 6) or chr8 normal status (Supplemental Figure 9). For these studies we used the FAKi VS-4718 as a surrogate for the clinical FAKi defactinib because although defactinib inhibits tumor FAK activity with tolerability in cancer patients (33), VS-4718 has better pharmacokinetics than defactinib in mice. Previous work from our lab identified MN2 and WU-225 as chr8 gain–associated MPNST PDX models, while WU-487 and JH-2-055 were determined to be chr8 diploid models (22, 23). VS-4718 and/or avutometinib were administered orally at a dose of 50 mg/kg bid and 0.3 mg/kg qd, respectively, over the course of 4 to 5 weeks. This dosing regimen aimed to evaluate the therapeutic efficacy and potential synergistic effects of combining these agents in preclinical models.

In the chr8 gain MN2 PDX model, FAKi as a single agent moderately inhibited tumor growth, with a tumor growth inhibition (TGI) of 57.17% at the end of treatment. RAF/MEKi treatment yielded a TGI of 79.49%, while the combination therapy resulted in a TGI of 96.17% (Figure 6A). Remarkably, complete tumor regression was observed in 4 of 6 mice following 3 weeks of combination therapy (Supplemental Figure 10A). In the chr8 gain WU-225 PDX model, FAKi monotherapy was less effective, achieving a TGI of 15.55%, while RAF/MEKi treatment resulted in a TGI of 56.13%, and the combination therapy reached a TGI of 75.85% (Figure 6B). In contrast, in the chr8 diploid models WU-487 and JH-2-055, FAKi as a single agent did not show significant effect on tumor growth (Supplemental Figure 9). Further, the combination treatment did not significantly improve efficacy compared to RAF/MEKi alone. In WU-487, the combination achieved a TGI of 61.99% (Supplemental Figure 9A), while in JH-2-055, a similar trend was observed, with a TGI value of 49.39% for the combination group (Supplemental Figure 9B).

Survival analysis demonstrated a consistent benefit of combination therapy in chr8 gain models. In MN2, median survival was 64 days for the combination group, representing an extension of 21% (11 days) increase over RAF/MEKi (53 days), a 64% increase over FAKi (39 days), and a 137% increase over vehicle (27 days). In WU-225, combination therapy extended survival to 77 days, corresponding to a 10% increase over RAF/MEKi (70 days) and a 38% increase over FAKi or vehicle (56 days). In contrast, the survival advantage in chr8 diploid models was more modest: WU-487 showed 76 days for combination, a 6% increase over RAF/MEKi (72 days), 21% over FAKi (63 days), and 24% over vehicle (58 days), while JH-2-055 showed 86 days for combination, only a 3% increase over single agents (83 days) and 11% over vehicle (77.5 days). (Supplemental Figure 10B). Overall, the treatment was well tolerated, with no significant effects on animal body weight observed across all single-agent and combination therapy groups (Supplemental Figure 11).

In a 10-day pharmacodynamic study with MN2 PDX, avutometinib almost completely inhibited phosphorylated ERK while increasing phosphorylation of FAK and AKT, both as a single agent and combination treatment. VS-4718, the FAKi, effectively suppressed FAK activity in both the single-agent and combination groups, thereby enhancing the induction of cleavage of caspase-3. Notably, Ki67 staining did not significantly differ between the treatment groups. However, this could have been influenced by the timing of sample collection (10 days of treatment). In addition, the increase in the number of cells positive for cleaved caspase-3 suggests that the enhanced antitumor activity of the combination is not driven by suppression of proliferation but rather by increased induction of cell death (Figure 6, C and D, and Supplemental Figure 12).

These results suggest that the combination of FAKi with RAF/MEKi significantly reduces tumor growth in chr8 gain MPNST PDX models, while the effect is less pronounced in chr8 diploid models. In vivo data further confirmed that suppressing compensatory signaling pathways, such as the FAK/AKT pathway activated by RAF/MEK inhibition, could overcome resistance mechanisms that limit the efficacy of single-agent treatment (Figure 6D).

## Discussion

We previously identified chr8 gain as the most common copy number event in MPNST, and it is associated with poor overall survival. Herein, we have made several important discoveries. First, through a CRISPR screen, we identified 58 genes located on chr8 that influence MPNST survival, including *PTK2* (encoding FAK), a pivotal regulator of cell survival, proliferation, and migration. Second, *PTK2* knockdown reduced growth of MPNST cells in vitro and tumors in vivo, and the FAKi defactinib suppressed cell proliferation and induced apoptosis in MPNST cell lines. Third, our data demonstrate that FAK signaling in MPNST involves the PI3K/AKT pathway, the MEK/MAPK pathway, and the transcription factor STAT3, and the combination of defactinib and the RAF/MEKi avutometinib synergistically reduced proliferation and increased cell death in chr8 gain MPNST cell lines. Finally, we explored the combination therapy in vivo and demonstrated that this combination therapy had greater efficacy in chr8 gain models (MN-2 and WU-225) compared with diploid models (WU-487 and JH-2-055).

Mechanistically, one of the primary pathways influenced by FAK in a variety of cell types is the PI3K/AKT signaling axis, which promotes cell survival and resistance to apoptosis processes critical for sustaining cancer growth (37). Additionally, the interaction of FAK with the transcription factor STAT3, which is heavily involved in tumor progression and inflammation, further amplifies its function in oncogenic signaling (38). FAK-related pathways have also been nominated as responsible for persister cells or drug-tolerant residual disease following inhibition of ERK signaling pathways in various cancers (39, 40). Collectively, these pathways highlight the central role of FAK in cancer-related signaling networks, making it a compelling target for therapeutic intervention.

Despite the importance of FAK in these oncogenic processes, preclinical models and clinical studies have shown that FAKi as monotherapy exhibit limited efficacy in treating cancers (41, 42). However, FAK has emerged as a crucial alternative signaling mechanism in the development of resistance to chemotherapy (43, 44), radiotherapy (45, 46), and targeted therapies (39, 40, 47), highlighting the importance of rational combination strategies. Targeting multiple pathways, such as FAK/AKT and RAS/MAPK, can improve efficacy, overcome resistance, and limit toxicity. Such approaches are especially promising for aggressive tumors like MPNST, where single-agent treatments often fall short. Building on this rationale, a triple-combination strategy incorporating chemotherapy with MEK and FAK inhibition may further improve outcomes by blocking therapy-induced adaptive signaling and potentiating cytotoxic effects. There is also a strong rationale for combining this approach with radiotherapy, as radiation can activate prosurvival FAK and MAPK pathways; concurrent inhibition may enhance radiosensitivity and mitigate resistance (48). Supporting this concept, ongoing clinical trials are evaluating the combination of stereotactic body radiation therapy with defactinib (NCT04331041) or defactinib/avutometinib (NCT07126158) in advanced pancreatic cancer. These strategies warrant further investigation, particularly in aggressive malignancies such as MPNST.

Given the dysregulated RAS signaling observed in MPNST because of loss of *NF1*, MEK has been a logical target to explore. However, MEKi have failed to achieve durable ERK signaling inhibition in MPNST, likely due to activation of RTKs or adaptive resistance resulting from ERK negative feedback release (18). Notably, MEKi-treated MPNST cells exhibit upregulation of FAK-related genes, including *PTK2B* (*PYK2*), *RHOA*, *R-RAS*, and Src family kinases genes *FYN* and *LCK*, along with increased expression of several genes encoding integrins and laminin-related proteins (Supplemental Figure 8A) (17, 36). This suggests activation of FAK pathway contributing to therapeutic resistance. Additionally, MEK inhibition has been reported to reactivate C-RAF and induce RAF/MEK complex formation, potentially contributing to MEKi resistance (29). Avutometinib is a dual RAF/MEKi that targets the RAS/MAPK pathway through a unique mechanism inhibiting both RAF and MEK kinases, aiming to enhance anticancer efficacy. The combination of avutometinib with the FAKi defactinib received FDA accelerated approval for the treatment of patients with KRAS-mutated, low-grade serous ovarian cancer (LGSOC) based on data from the phase II study RAMP 201 (34). In patients with KRAS-mutated LGSOC, overall response rate was 44% (25/57), and the median progression-free survival was 22.0 months (34). A randomized phase III study of avutometinib and defactinib versus investigator’s choice of therapy for women with recurrent LGSOC is currently enrolling (49). While LGSOC is a fairly indolent disease, the combination of avutometinib and defactinib is also being investigated in additional, more aggressive, RAS/MAPK pathway–driven malignancies. The phase I FRAME trial demonstrated tolerability and clinical activity across multiple advanced solid tumors, including RAS- or RAF-mutant endometrioid gynecologic tumors, KRAS-mutant colorectal cancer, KRAS-mutant pancreatic cancer, and KRAS G12V-mutant non–small cell lung cancer (34). Consistent with these findings, the phase I/II RAMP 205 study in metastatic pancreatic ductal adenocarcinoma (NCT05669482) showed encouraging activity for the combination in conjunction with standard-of-care chemotherapy. Ongoing trials are evaluating this regimen in KRAS-mutant non–small cell lung cancer (NCT05074810) and colorectal cancer (NCT05554354). The available data demonstrate durable efficacy and tolerability, supporting further investigation of this combination for the treatment of MPNST and other aggressive, high-grade malignancies.

Indeed, our in vivo preclinical studies show that the FAKi and RAF/MEKi combination decreased tumor growth over either drug alone in chr8 gain MPNST PDX mouse models, with weaker response in chr8 diploid models. Consistently, in vitro data in JH-2-055-d cells (chr8 diploid) showed a higher IC_50_ for defactinib relative to cells with ch8 gain (Supplemental Table 2), with a slightly enhanced effect when combined with avutometinib (Supplemental Figure 5). These data suggest that chr8 gain models with FAK overexpression are more sensitive to the combination of MEK/FAK inhibition and that this genomic event could serve as a biomarker for response in clinical trials.

Notably, although the combination therapy demonstrated robust TGI in chr8 gain PDX models, activity was also observed in chr8 diploid models. The apparent reduced efficacy in diploid tumors may reflect their slower baseline growth rates, which may limit the ability to detect separation between vehicle- and treatment-treated tumors within the study period. Thus, while the magnitude of response is less pronounced, these data do not rule out meaningful activity of the combination in chr8 diploid MPNST.

Moreover, NF1 negatively regulates FAK signaling through direct interaction between the C-terminal domain of NF1 and the N-terminal domain of FAK (50). Loss of NF1 leads to activation of the FAK pathway. This direct protein interaction between NF1 and FAK may also contribute to the observed synergistic effects. While one hypothesis is that chr8 gain drives FAK overexpression and part of the interaction reflects adaptation to MEK inhibition, it is also possible that the synergy arises from the direct effect of NF1 loss in enhancing FAK activity.

In addition to FAK, other genes on chr8, such as *MYC* (51) and *FGFR1* (52, 53), are well-known oncogenes implicated in multiple cancers, including sarcomas (51, 53). Based on their statistical significance in our CRISPR screen and previously reported roles in cancer biology, genes such as *UBR5* (54), *GLI4* (55), *PUF60* (56–58), *COPS5* (59, 60), *EXT1* (61), *MCM4* (62, 63), *PSD3* (64), and *SDC2* (65, 66) may also contribute to the malignant behavior of MPNST. While further validation is needed, these findings point to several chr8 genes that may represent therapeutic vulnerabilities in MPNST and other solid tumors with chr8 gain.

In summary, we have identified that the chr8 gene *PTK2* (FAK) plays an essential role in MPNST pathogenesis. FAK inhibition, both as a monotherapy and in combination with RAF/MEKi, shows promise in PDX models of MPNST, particularly in tumors with chr8 gain, supporting FAK/RAF/MEK cotargeting as a promising therapeutic approach for chr8 gain tumors such as MPNST. In addition, considering that patients with NF1 who develop MPNST may have previously received MEKi therapy, we performed preliminary in vitro studies using trametinib-resistant cells and found that FAK or FAK/RAF/MEK inhibition remained effective in these resistant models (Supplemental Figure 13). Collectively, these data lay the groundwork for a biomarker-driven clinical trial in MPNST, which is currently in development by the NF Clinical Trials Consortium. They also present a potential research avenue in other malignancies with chr8 gain.

## Methods

### Sex as a biological variable.

There has not been a published observation of sex difference in the risk of development of MPNST (67, 68). Sex was not specifically evaluated as a biological variable in this study. For consistency in animal housing, our study examined female mice. Cell lines and PDX were derived from both male and female patients.

### Cell lines and culture reagents.

Murine MPNST cell line JW23.3 and human MPNST cell line WU-356 were generated in our lab (23, 69). Human MPNST cell lines JH-2-002 and JH-2-055-d were obtained from the Pratilas lab at JHU/the JH NF1 biospecimen repository (70, 71). HEK293T cells were purchased from ATCC. JW23.3 and HEK293T cells were grown in DMEM (10-013-CM, Corning) supplemented with 10% FBS (Gibco) and 100 μg/mL primocin (ant-pm-2, InvivoGen). JH-2-002 and JH-2-055-d cells were grown in RPMI medium 1640 (11875-085, Gibco) supplemented with 20% FBS and 100 μg/mL primocin. WU-356 cells were cultured in modified sarcoma growth media as described previously (72), containing DMEM/Ham’s F12 medium (1:1; Mediatech), supplemented with 10% FBS (Gibco), 12.5 μg/mL bovine pituitary extract (13028014, Invitrogen), 1% ITS+ (41400045, Thermo Fisher Scientific), 0.5 ng/mL basic fibroblast growth factor (100-18B-10UG, PeproTech), 1 ng/mL epidermal growth factor (AF-100-15-100UG, PeproTech), 0.1 ng/mL TGF-β1 (100-21-2UG, PeproTech), and 100 μg/mL primocin (InvivoGen). All cells were maintained at 37°C in a humidified atmosphere at 5% CO_2_.

### Compounds.

Defactinib, VS-4718, and avutometinib were provided by Verastem Oncology. For in vitro studies, compounds were dissolved in dimethyl sulfoxide (DMSO) (D2650, Sigma). For in vivo studies, VS-4718 was dissolved in 0.5% methyl cellulose (274429, Sigma)/0.1% Tween 80 (S6702, Selleckchem), and avutometinib was dissolved in 10% captisol (NC-05E-21039, Cydex Pharmaceuticals) and 5% DMSO.

### CRISPR gRNA screen.

A CRISPR/Cas9 gRNA library specifically targeting genes located on chr8 was employed to identify genes essential for the survival of MPNST cells. The gRNA library was specifically designed to target the coding regions of chr8 genes, with 3–4 gRNAs per gene to ensure thorough coverage. Lentiviral vectors carrying Cas9 were transduced into JH-2-002 MPNST cells under blasticidin (5 μg/mL) selection. Then the lentiviral vectors carrying the gRNA library transduced at a low multiplicity of infection (<0.3), ensuring that each cell incorporated a single gRNA.

Following transduction, cells were subjected to dual selection with puromycin (1 μg/mL) and blasticidin (5 μg/mL) to enrich for populations carrying both Cas9 and integrated gRNAs.

The cells were maintained and passaged for 34 days to allow for the depletion of cells in which essential genes had been knocked out. During the screening period, genomic DNA was extracted from the surviving cells on days 0, 10, 24, 30, and 34, and the integrated gRNAs were amplified via PCR and analyzed through next-generation sequencing to quantify gRNA abundance. Sequencing data were then processed to map the gRNAs to their corresponding target genes on chr8. A comparison between the initial library distribution and the gRNA abundance in the postscreening cell population allowed for the identification of candidate genes essential for MPNST survival, as indicated by the significant depletion of their corresponding gRNAs.

### Analysis of TCGA Firehose Legacy Sarcoma data.

To investigate the association between overall survival and genomic alterations in druggable targets identified from the CRISPR screen, TCGA Firehose Legacy Sarcoma dataset was queried using cBioPortal (http://cbioportal.org). Druggable targets among the significant genes in the CRISPR screen (*P* < 0.05) were identified using the LinkedOmics Target Explorer (https://targets.linkedomics.org/), which integrates Drug-Gene Interaction Database annotations to classify genes as potentially druggable by small molecules. Briefly, of the 1,075 genes cataloged in the database known to be targeted by drugs (i.e., assigned tiers 1–3 meaning approved for oncology, approved for other indications, or classified as investigational or experimental, respectively), 5 candidates (*ATP6V1B2*, *FGFR1*, *POLB*, *PTK2*, and *TOP1MT*) were identified among the CRISPR hits and selected for further analysis.

Out of 243 TCGA Firehose Legacy Sarcoma samples with mutation and CNA data, 236 samples had no mutations in any of these 5 druggable targets. CNA status was determined based on putative copy number alterations from the Genomic Identification of Significant Targets in Cancer (GISTIC) (73). Four genes (*FGFR1*, *POLB*, *PTK2*, and *TOP1MT*) showed recurrent amplifications, whereas *ATP6V1B2* exhibited no significant alterations and was excluded from survival analyses. Association between copy number amplification in these 236 samples with overall survival was evaluated using the Kaplan-Meier survival analysis tool available on cBioPortal. The amplification is defined on cBioPortal using the GISTIC algorithm, which scores copy number changes as –2 (homozygous deletion), –1 (heterozygous loss), 0 (diploid/no change), 1 (low-level gain), and 2 (high-level amplification). We only selected those as “high-level” amplification for the analysis. Survival differences between amplified and diploid groups were analyzed using the log-rank test, with *P* values less than 0.05 reported for statistical significance.

### PTK2 expression and survival analysis in MPNST patient samples.

MPNST patient samples (*n* = 94) were collected from Washington University in St. Louis, JHU, and the University of Minnesota. For DNA-sequencing data, FASTQ files were trimmed using Trimmomatic v0.39 (74) and aligned to the human reference genome hg38 using BWA-MEM. Duplicate reads were marked using Picard (“MarkDuplicates”). GATK V4.2 base quality score recalibration was used to process BAM files. Copy number variations (CNVs) were detected using CNVkit (75). The chr8 status was determined based on absolute copy number (CN) thresholds: gain if CN > 2.4, and diploid if 1.6 ≤ CN ≤ 2.4.

For RNA sequencing, raw reads were processed using the nf-core/rnaseq pipeline (v3.14.0) (76), implemented with Nextflow. Adapter and quality trimming was performed with Trim Galore, and initial read quality was assessed using FastQC, with summaries compiled by MultiQC. Reads were aligned to the human reference genome (GRCh38/hg38) using STAR, and transcript abundance was estimated using Salmon in alignment-based mode with GENCODE v40 annotation. Gene-level expression values, including raw counts and TPMs, were obtained from *quant.genes.sf* files for downstream analyses. Difference of *PTK2* expression was visualized using VST normalization from DESeq2 package (77).

For survival analysis, patients with MPNST from the JHU cohort, GEM, and GSE241224 (*n* = 132) were stratified into high- and low-*PTK2* expression groups using the median *PTK2* expression value (9.951) as the cutoff. Among these samples, chr8 status included chr8 gain (*n* = 32), chr8 loss (*n* = 3), chr8 diploid/normal (*n* = 35), and NA (*n* = 62) for cases without available chr8 status information. Log-rank test was used, with *P* values less than 0.05 reported for statistical significance.

### shRNA-mediated PTK2/FAK knockdown in MPNST cells.

Murine MPNST tumor cell line JW23.3, derived from C57BL6/J Nf1^+/−^ Trp53^+/−^ (NPcis) (69), and human MPNST tumor cell lines JH-2-002 and WU-356 were used for in vitro studies. JW23.3 and JH-2-002 were used for in vivo tumor growth experiments. Cells were transduced with either a control lentiviral vector targeting *LacZ* (Genome Institute at Washington University in St. Louis) or lentiviral vectors carrying independent shRNAs targeting murine *PTK2* (Sigma-Aldrich; TRCN0000321963, or TRCN0000023485) or human *PTK2* (Sigma-Aldrich; TRCN0000121129, TRCN0000121318, TRCN0000196310, or TRCN0000194984) (Supplemental Table 3). Two stable cell lines exhibiting the best knockdown effects, as determined by protein and mRNA analysis, were selected and expanded for subsequent experiments.

### Cell proliferation and death assay.

For the cell proliferation assay of *PTK2*-knockdown cells, cells were seeded into 96-well plates, and scanning was initiated 6 hours later, once the control cells had attached to the plates.

For the antiproliferation assay of compounds, cells were seeded in 96-well plates and treated with different concentrations of drugs for the indicated times. YOYO-1 iodide (Y3601, Invitrogen, Thermo Fisher Scientific), at a ratio of 1:80,000, was added into the media for the detection of cell death. Each treatment was tested in quadruplicate. The cell confluence (a measure of cell proliferation and antiproliferative effect) and dead cell counts were determined using Incucyte S3 Live-Cell Analysis System (Sartorius). Images were analyzed with Incucyte S3 analysis software module (Sartorius). IC_50_ values were calculated with GraphPad Prism software (version 10.0; GraphPad Software) using nonlinear regression (curve fitting) analysis.

For combination studies, concentrations around IC_50_ were used to enable assessment of combinatorial effects while minimizing single-agent cytotoxicity. For synergy calculations of drug combinations, Incucyte dose response data for cell proliferation or cell death, normalized to maximum inhibition values, were analyzed using SynergyFinder software (www.synergyfinderplus.org) and the HSA model. Synergy scores > 10 were considered synergistic, while –10 to 10 was additive, and <–10 was antagonistic.

### Quantitative reverse transcriptase PCR analysis for PTK2 transcripts.

Total RNA was isolated from cells using the RNeasy Mini Kit (74104, QIAGEN) according to the manufacturer’s protocol, and RNA concentration and purity were assessed using a NanoDrop 1000 spectrophotometer (Thermo Fisher Scientific). cDNA was synthesized from total RNA by reverse transcription using the High-Capacity RNA-to-cDNA Kit (4387406, Applied Biosystems, Thermo Fisher Scientific). Quantitative PCR (qPCR) was carried out on a CFX96 Real-Time PCR Detection System (Bio-Rad) using the Power SYBR Green qPCR Kit (4309155, Applied Biosystems, Thermo Fisher Scientific). Primers were synthesized by Thermo Fisher Scientific and are listed in Supplemental Table 4. Each reaction was performed in triplicate, and melting curve analysis was included to confirm product specificity. Data were analyzed using Bio-Rad CFX Manager Software (version 3.1, 2017), and gene expression levels were normalized to 18S rRNA as the internal control using the 2^-ΔΔCt^ method.

### WES Western blotting assay.

After treatments with drugs for indicated times, cultured cells were harvested and lysed in Cell Lysis Buffer (9803, Cell Signaling Technology) containing 1% protease inhibitors (P8340, Sigma-Aldrich). Tumor tissues were lysed in RIPA (9806, Cell Signaling Technology) containing 1% protease inhibitors (P8340, Sigma-Aldrich). Protein concentrations were determined using the Pierce BCA Protein Assay Kit (23225, Thermo Fisher Scientific). Equal amounts of cell protein lysates (0.25 μg) were separated and analyzed on a Simple Western WES system (Bio-Techne) using a 12–230 kDa Separation Module (SM-W001, Bio-Techne). Primary antibodies including rabbit anti Phospho-FAK (Tyr397) (#3283), FAK (#3285), Phospho-p44/42 MAPK (Erk1/2) (Thr202/Tyr204) (#4370), p44/42 MAPK (Erk1/2) (#4695), Phospho-STAT3 (Tyr705) (D3A7) (#9145), STAT3 (#4904), Phospho-Akt (Ser473) (#4060), Akt(pan) (#4691), Caspase-3 (#9662), PARP (#9542), and β-Actin (#4967 and #4970) antibodies were purchased from Cell Signaling Technology. Primary rabbit anti-GAPDH (TAB1001, Invitrogen) antibody was purchased from Thermo Fisher Scientific. Anti-Rabbit Detection Module (DM-001, Bio-Techne) was used to detect the targeted primary antibodies.

For the FAK protein expression analysis in patient samples, MPNST specimens (JH-2-002-g, JH-2-031, JH-2-084, JH-2-117-b, JH-2-139, JH-2-201-c, JH-2-242-c, JH-2-295-b, JH-2-379-a, and JH-2-340) and pNF specimens (JH-2-001, JH-2-002, JH-2-010, JH-2-023-c, JH-2-100, JH-2-120-d, JH-2-125, JH-2-140, JH-2-150, and JH-2-252-a) were provided by the Pratilas lab at JHU/the JH NF1 biospecimen repository.

### IHC and H&E staining.

Tumor tissues were harvested and fixed in 10% neutral-buffered formalin (245-486, Thermo Fisher Scientific). Paraffin-embedding, sectioning (5 μm), and H&E staining were processed by the Musculoskeletal Histology and Morphometry Core of Washington University in St. Louis.

IHC was conducted on an automated Roche Discovery Ultra system as per manufacturer’s instructions. Briefly, sections were deparaffinized and rehydrated, followed by antigen retrieval using citrate-based buffer (CC1, 950-500, Roche). Endogenous peroxidase activity was quenched with hydrogen peroxide. Slides were incubated with primary antibodies at the indicated concentrations and nonspecific binding was blocked using 5% goat serum (S-1000, Vector Laboratories). Primary antibodies included pFAK (1:400; Invitrogen, Thermo Fisher Scientific, 700255), total FAK (1:50; Cell Signaling Technology, 3285S), pERK (1:400; Cell Signaling Technology, 4370S), pSTAT3 (1:400; Cell Signaling Technology, 9145), Cleaved Caspase-3 (Asp175) (1:400; Cell Signaling Technology, 9661), and Ki-67 (manufacturer’s concentration; Roche, 790-4286). Then slides were exposed to Discovery OmniMap anti-Rabbit HRP secondary antibody (Roche, 760-4311). Chromogen detection was performed with Discovery ChromoMap Diaminobenzidine (DAB) RUO (Roche, 760-159) followed by counterstaining with hematoxylin (Roche, 790-2208), and slides were dehydrated and mounted.

Images were acquired using a bright-field microscope (P250 Pannoramic scanner), and staining intensity and distribution were evaluated qualitatively using image analysis software ImageJ.

### In vivo PDX models.

Female 4- to 8-week-old mice were used for in vivo studies. The in vivo antitumor effects of *PTK2* knockdown were assessed using murine MPNST JW23.3 cells and human MPNST JH-2-002 cells expressing shRNA to achieve effective knockdown of *PTK2* expression. JW23.3 cells were injected subcutaneously into C57BL/6J mice (strain 000664, The Jackson Laboratory) at 1 × 10^6^ cells in 100 μL PBS per mouse, 5 mice per group. JH-2-002 cells, derived from a male patient, at 1 × 10^5^ cells in 5 μL PBS per nerve, were injected orthotopically into the sciatic nerve of nude mice (strain 002019, The Jackson Laboratory). Bilateral injections were performed in 5–7 mice per group. The study endpoint was the development of leg limping and a palpable mass in the leg. Tumor tissues were harvested at the endpoint and fixed in 10% formalin or frozen for subsequent analyses.

The in vivo antitumor effects of compounds were assessed using MPNST PDX models, specifically MN2 (female) and WU-225 (male), which exhibit chr8 gain, and WU-487 (male) and JH-2-055 (female), which have diploid chr8 status. MN2, WU-487, and JH-2-055 were implanted into NRG mice (strain 007799, The Jackson Laboratory), and the WU-225 line was implanted into nude mice (strain 002019, The Jackson Laboratory). PDX tumor models were established by subcutaneously implanting 3 mm × 3 mm tumor pieces under sterile conditions. Mice were randomized into treatment groups, with a minimum of 5 mice per group, once tumor volumes reached 50–150 mm^3^. The FAKi VS-4718 was administered at a dose of 50 mg/kg via oral gavage twice daily, 5 days per week for 4 weeks. Additionally, the RAF/MEKi avutometinib was administered at 0.3 mg/kg via oral gavage once daily, 5 days per week for 4 weeks.

For pharmacodynamic studies, MN2 tumor was implanted into NSG mice (strain 005557, The Jackson Laboratory) with a minimum of 3 mice per group. Once tumor volumes reached 100–300 mm^3^, VS-4718 was administered twice daily, and avutometinib was administered once daily for 10 days. Tumor tissues were harvested 2 hours after the final dose for subsequent analyses. A total of 100–200 μL blood was collected from the submandibular vein at the indicated time points for further analyses.

Animal body weight and tumor size were measured twice weekly. Tumor volume (TV) was calculated according to the following equation, TV = L × W^2^/2, where L indicates length of the tumor and W indicates width of the tumor. TGI (%) = (TV_control_ – TV_treatment_) / TV_control_ × 100.

Treatment information for patients at the time of PDX and cell line generation is summarized in Supplemental Table 5.

### Statistics.

A 1-way ANOVA was used for comparisons among multiple groups, while a Student’s *t* test was applied to assess differences between 2 groups in both in vitro and in vivo experiments. When ANOVA results were significant, post hoc multiple-comparison test (Tukey’s test) was performed to identify specific pairwise differences. All statistical tests were 2-sided, and data are presented as the mean ± SEM unless otherwise specified. Survival analyses were conducted using the Kaplan-Meier method, and survival distributions were compared with the log-rank (Mantel-Cox) test. A *P* < 0.05 was considered statistically significant. GraphPad Prism software (version 10.0; GraphPad Software) was used for all statistical analyses and to generate graphical presentations.

### Study approval.

All animal experiments were conducted in accordance with the protocol approved by the Institutional Animal Care and Use Committee at Washington University in St. Louis, St. Louis, Missouri, USA (protocol 22-0157, 22-0041, and 25-0069). In accordance with the ethical principles and research guidelines stipulated by the U.S. Common Rule, the use of patient samples in this study was approved by the Institutional Review Board of Johns Hopkins University, Baltimore, MD, USA, or Washington University in St. Louis, MO, USA. Written informed consent was obtained from the patients, or the requirement for informed consent was waived by the Institutional Review Board.

### Data availability.

The datasets for CRISPR screening have been deposited in the Synapse repository at https://www.synapse.org/Synapse:syn53128248/wiki/635415 Values for all data points shown in the graphs are provided in the Supporting Data Values XLS file to facilitate reuse and reproducibility. All other data are available within the main manuscript or the supplement.

## Author contributions

ACH and SG obtained funding. ACH, SJCG, and GFW designed the research studies. GFW, JW, XZ, LY, MIZ, and AUB conducted experiments and acquired data. GFW, BBG, KX, JJ Szymanski, GO, and JJ Sears analyzed data. CAP and JW provided cell lines, PDX, and sequencing data resources produced at JHU. RR, SC and JAP provided compounds. GFW, DCB, and ACH wrote the manuscript. All authors contributed to editing the manuscript.

## Conflict of interest

ACH has been a consultant for Aadi Subsidiary, AstraZeneca Pharmaceuticals LP/Alexion, and SpringWorks Therapeutics; has licensing agreements with Boehringer Ingelheim GmbH and Deutsches Krebsforschungszentrum; and has received research support from Tango Therapeutics and Polaris Pharmaceuticals. JW and CAP have a pending patent (US20240238284A1); CAP has an active patent (US7812143B2); and JW and CAP have received research support from Kura Oncology and Novartis Institute for Biomedical Research. RR, SC, and JP work for Verastem Oncology.

## Funding support

This work is the result of NIH funding, in whole or in part, and is subject to the NIH Public Access Policy. Through acceptance of this federal funding, the NIH has been given a right to make the work publicly available in PubMed Central.

- CDMRP Neurofibromatosis Research Program (W81XWH2210324, ACH).
- St. Louis Men’s Group Against Cancer (ACH).
- Neurofibromatosis Therapeutic Acceleration Program (ACH).
- NIH NINDS (1R01NS134815-01, ACH).
- NIH NCI Cancer Center Support grant (P30 CA091842, Siteman Cancer Center).
- DOE (Contract DE-AC05-76RL01830, to Battelle Memorial Institute for operation of Pacific Northwest National Laboratory).

## Supplementary Material

Supplemental data

Unedited blot and gel images

Supporting data values

## Figures and Tables

**Figure 1:**
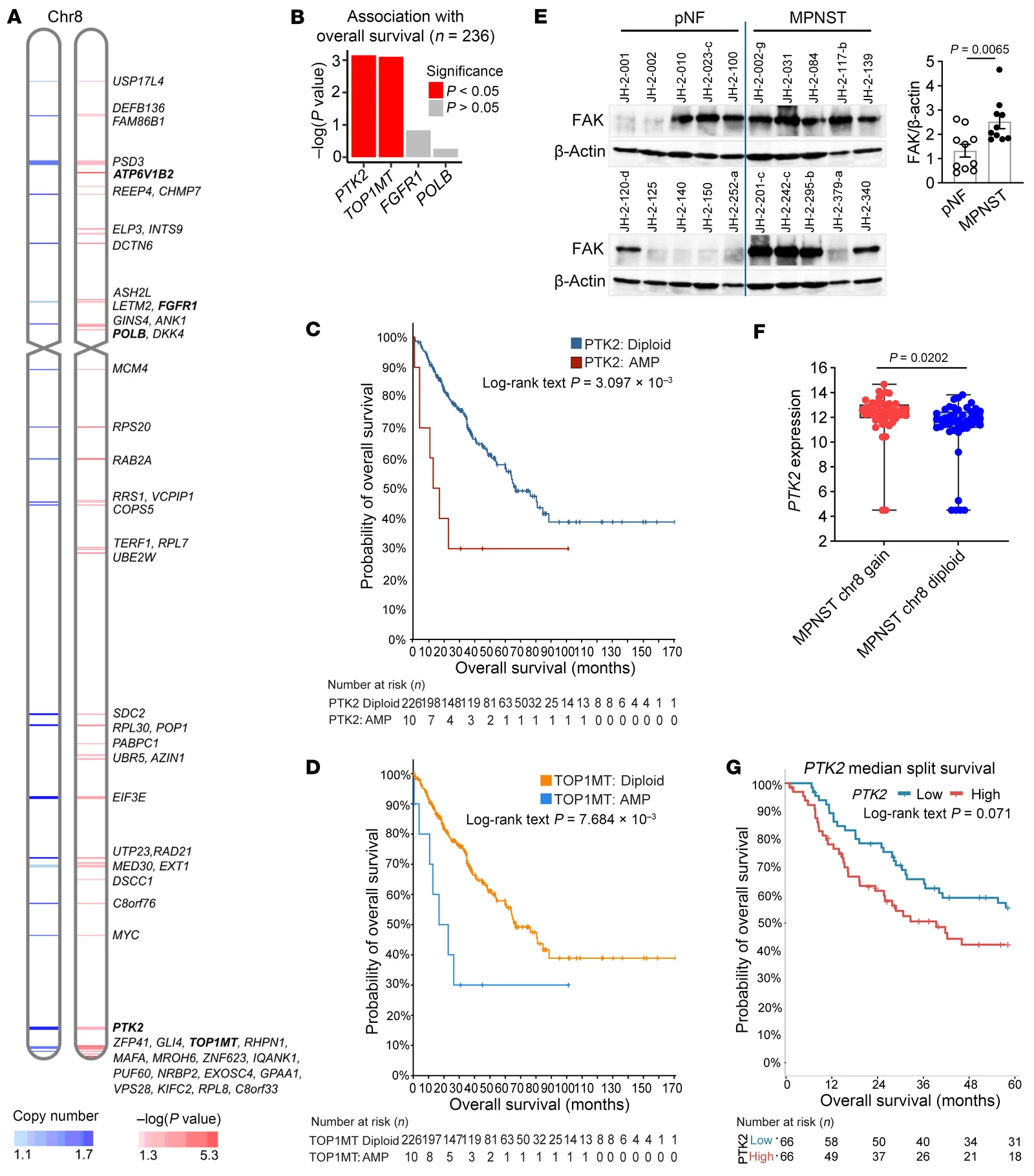
*PTK2* is an important gene on chr8 for survival of MPNST cells. (**A**) Location of all 58 genes in chr8 with *P* < 0.05 in CRISPR gRNA screening. (**B**) Association of gene amplification with overall survival in TCGA Firehose Legacy Sarcoma data (*n* = 236 for this analysis). Four of the 5 genes (*FGFR1*, *POLB*, *PTK2*, and *TOP1MT*) exhibited copy number amplifications. *ATP6V1B2* showed no significant alterations and is therefore not displayed. Association between copy number amplification with overall survival was evaluated using the Kaplan-Meier survival analysis. (**C**) Comparison of overall survival of patients with either *PTK2*-amplified or *PTK2*-diploid sarcoma in TCGA Firehose Legacy Sarcoma data. Differences in survival distributions were assessed using the log-rank test. (**D**) Comparison of overall survival of patients with wither *TOP1MT*-amplified or *TOP1MT*-diploid sarcoma in TCGA Firehose Legacy Sarcoma data. Differences in survival distributions were assessed using the log-rank test. (**E**) FAK (*PTK2*) overexpression in MPNST patient samples compared with pNF. Student’s *t* test was used for comparisons between 2 groups. (**F**) The gene expression (VST-normalized) of *PTK2* between chr8 diploid (*n* = 45) and chr8 gain (*n* = 49) MPNST samples. Student’s *t* test was used for comparisons between 2 groups. (**G**) Survival analysis of patients with MPNST from Johns Hopkins University (JHU) cohort, Genomics of MPNST (GEM), and NCBI GEO GSE241224 (*n* = 132). Differences in survival distributions were assessed using the log-rank test.

**Figure 2:**
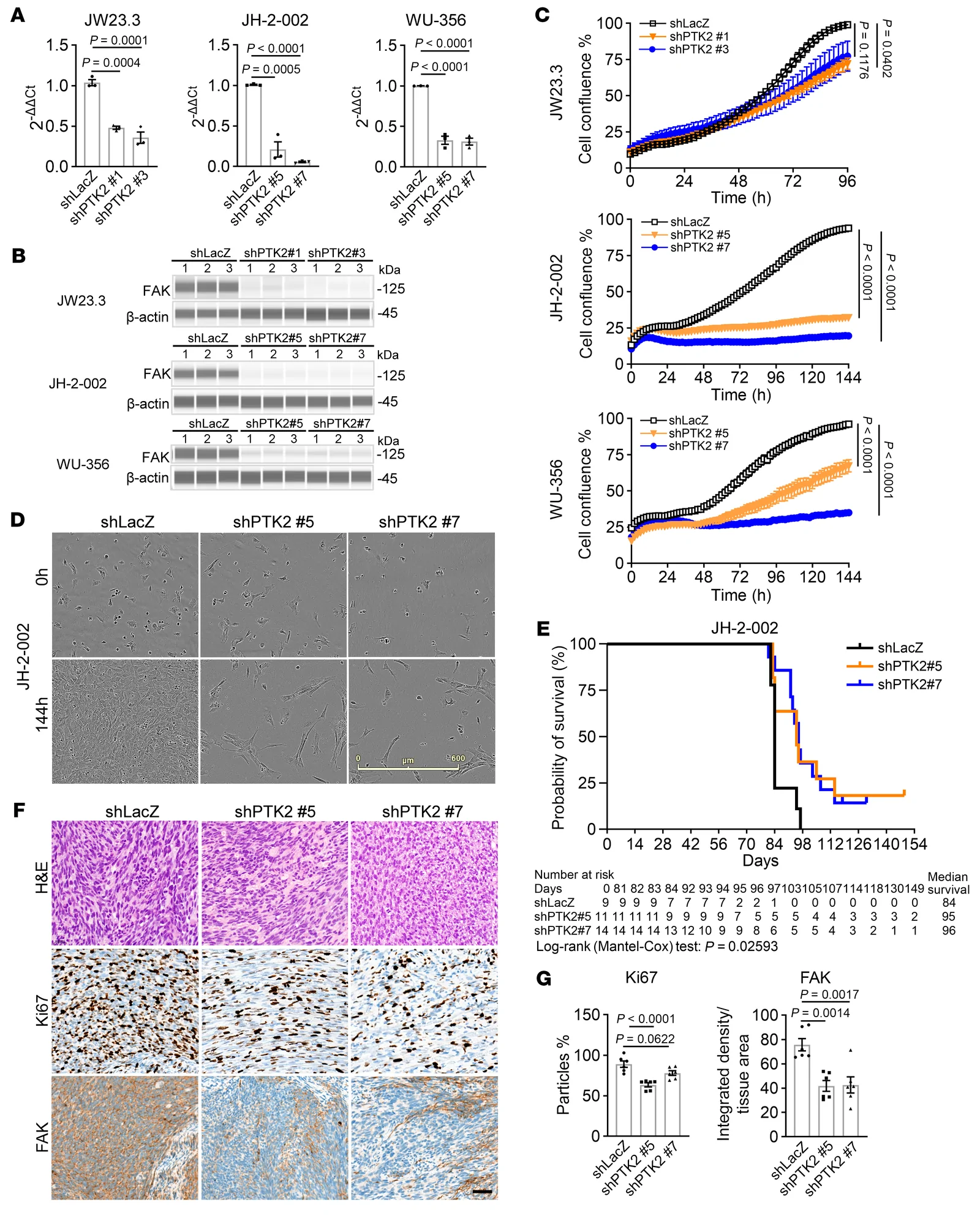
Knockdown of *PTK2* inhibits cell proliferation and tumorigenesis of MPNST cells. (**A**) Reverse transcription-PCR (RT-PCR) results demonstrated a significant decrease in *PTK2* mRNA in murine MPNST cells (JW23.3) and human MPNST cells (JH-2-002 and WU-356) following *PTK2* knockdown by shRNA. Statistical significance was determined using 1-way ANOVA followed by Tukey’s post hoc test for multiple-group comparisons. (**B**) FAK protein expression is decreased with *PTK2* shRNA knockdown in MPNST cell lines JW23.3, JH-2-002, and WU-356, as determined by Western blot (Simple WES) analysis. (**C**) Cell confluence over time after *PTK2* knockdown in MPNST cell lines JW23.3, JH-2-002, and WU-356. Cell confluence was monitored over time using the Incucyte system. Statistical significance was determined using 1-way ANOVA followed by Tukey’s post hoc test for multiple-group comparisons. (**D**) Representative images of Incucyte assay for JH-2-002 cells at 0 hour and 144 hours. (**E**) *PTK2* knockdown via shRNA prolonged survival in the MPNST cell JH-2-002 orthotopic mouse model. Kaplan-Meier survival analysis of nude mice injected orthotopically with 1 × 10^5^ JH-2-002 cells (5 μL) into the sciatic nerve, showing prolonged survival in the *PTK2*-knockdown groups. Survival distributions were compared using the log-rank (Mantel-Cox) test. (**F**) Histological (H&E) and immunohistochemical (Ki67 and FAK) staining of nerve tumor tissues. Scale bar, 50 μm. (**G**) IHC staining was quantified using ImageJ (NIH; http://imagej.org) by measuring integrated density or performing particle analysis. Statistical significance was determined using 1-way ANOVA followed by Tukey’s post hoc test for multiple-group comparisons.

**Figure 3:**
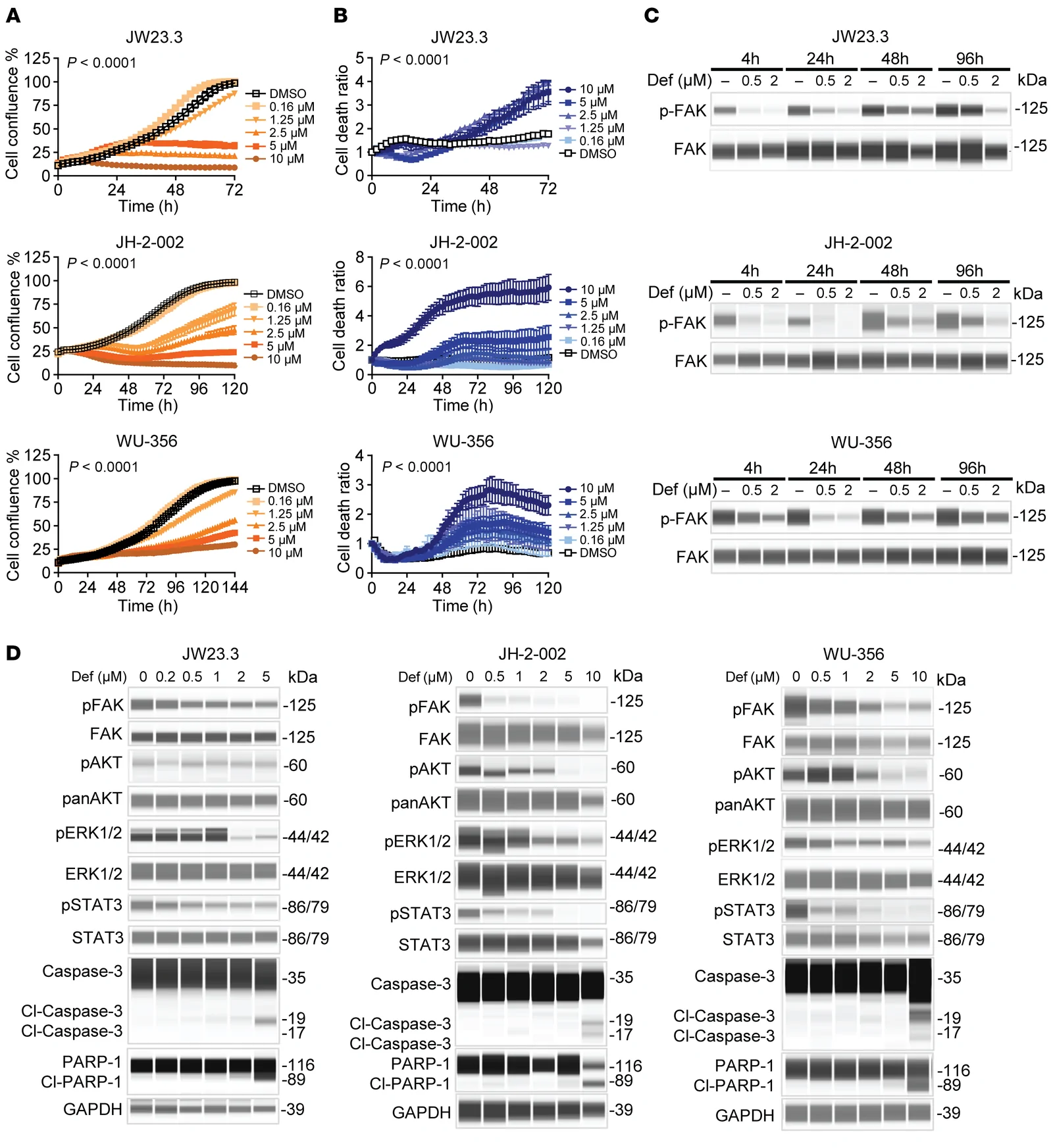
FAKi reduces cell proliferation and activation of FAK and downstream proteins in MPNST cells. Defactinib, a FAKi, inhibited cell proliferation (**A**) and induced cell death (**B**) in MPNST cell lines JW23.3, JH-2-002, and WU-356, as determined by Incucyte Live Cell assays. YOYO-1 green fluorescent dye was used as an indicator of cell death. The cell death ratio was determined by calculating the number of dead cells relative to cell confluence and normalizing this value to the baseline measurement at time 0. One-way ANOVA with Tukey’s post hoc test was used for multiple-group comparisons. (**C**) Defactinib as single agent time-dependently inhibited phosphorylation of FAK, as determined by WES Western analysis. (**D**) Defactinib dose-dependently inhibited phosphorylation of FAK and downstream AKT, STAT3, and ERK1/2 while augmenting cleavage of caspase-3 and PARP-1. GAPDH served as loading control. Cell lysates were collected after 48 hours of treatment. WES assays were repeated twice; representative images are shown.

**Figure 4:**
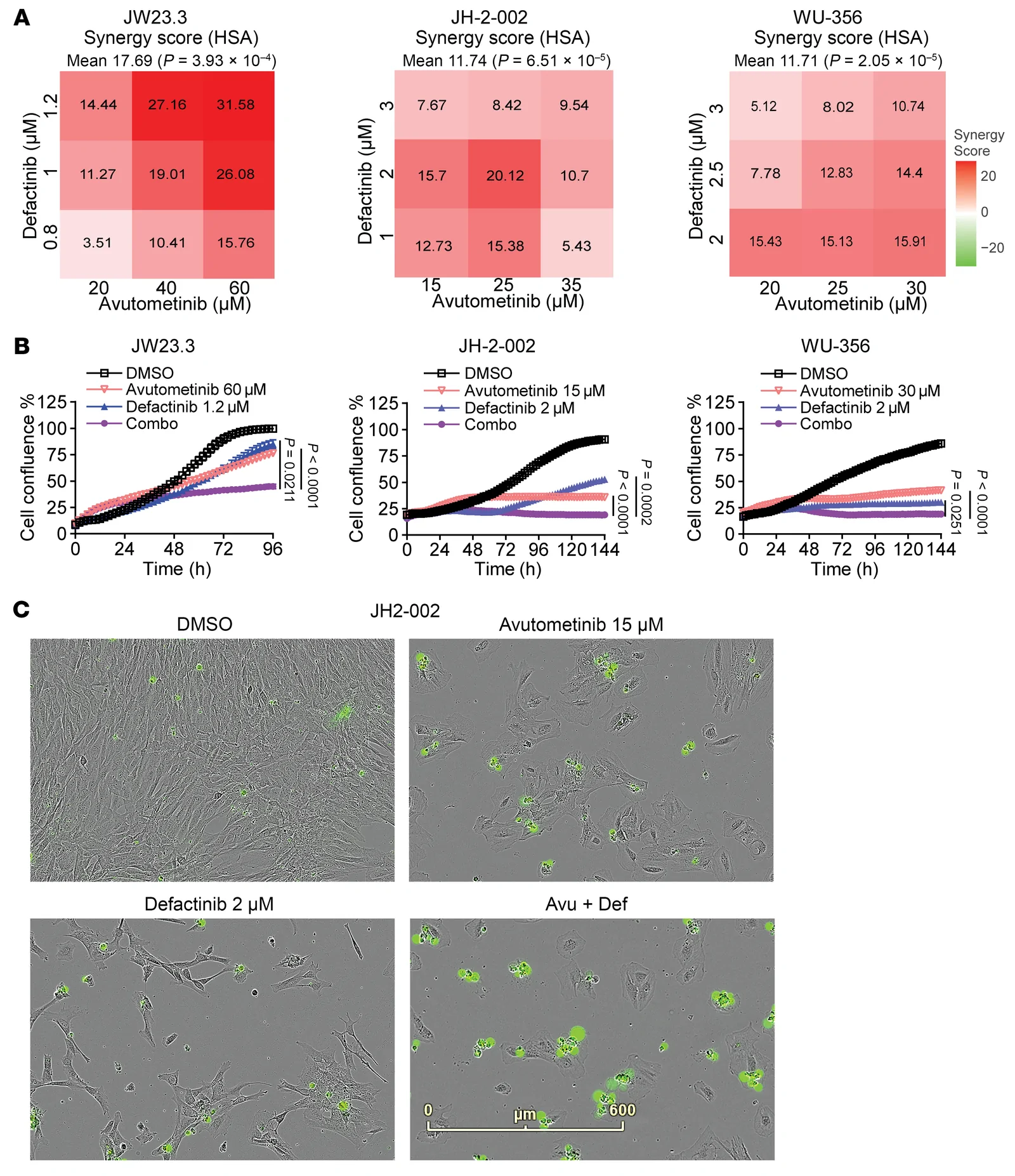
The combination of a FAKi and a RAF/MEKi is synergistic in decreasing proliferation of MPNST cells. (**A**) Heatmap of synergy scores for antiproliferation effect of the FAKi defactinib (Def) and the RAF/MEKi avutometinib (Avu) in JW23.3 (96 hours), JH-2-002 (144 hours), and WU-356 cells (144 hours), generated by using SynergyFinder. Synergy score > 10 indicates synergy. HSA, highest single agent. (**B**) Representative curves for cell confluences over time in Incucyte live-cell assays. One-way ANOVA with Tukey’s post hoc test was used for multiple-group comparisons. (**C**) Representative images of cell confluence in Incucyte assay at 144 hours for JH-2-002, using YOYO-1 green fluorescent dye as an indicator of cell death.

**Figure 5:**
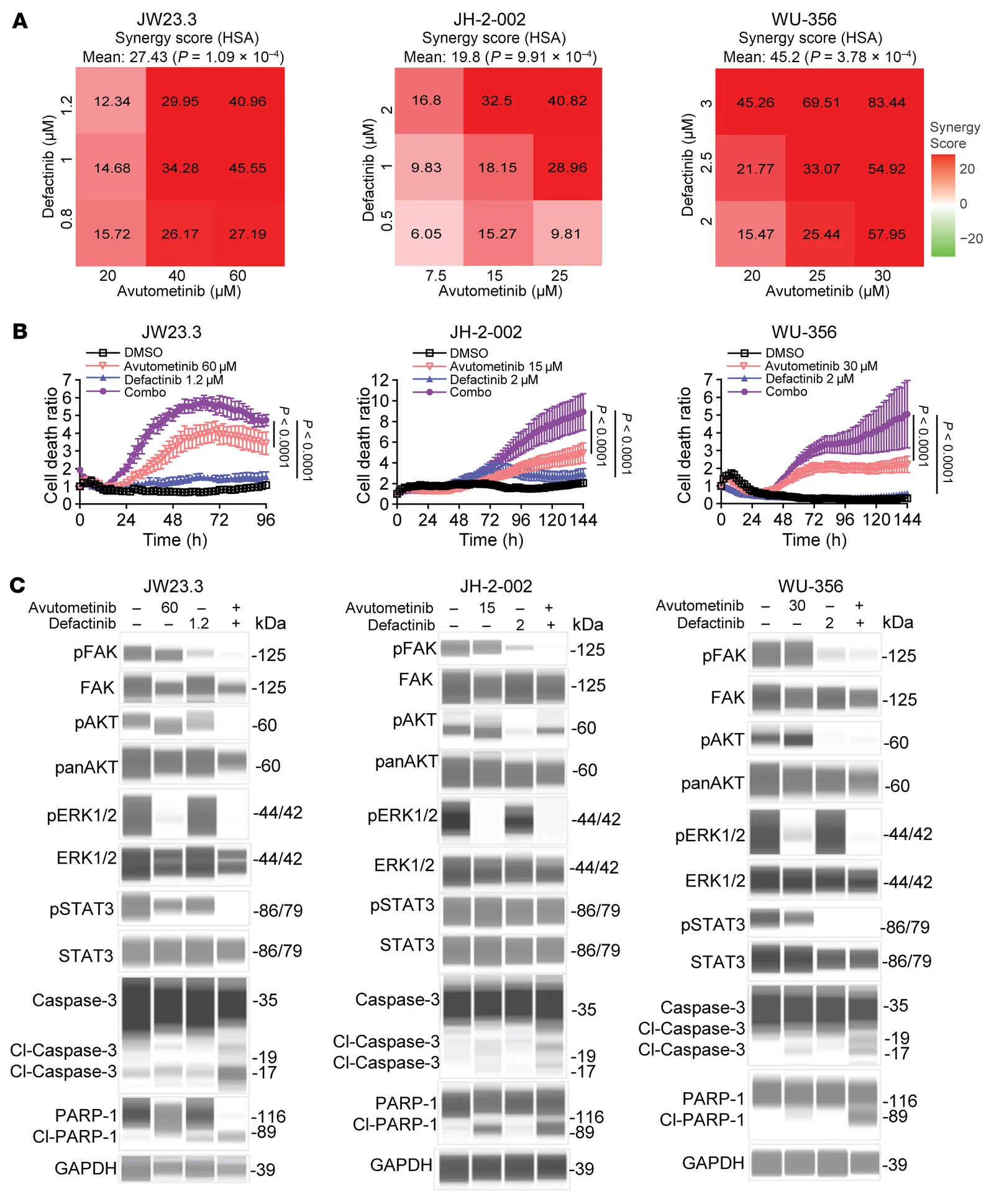
FAKi enhances the apoptotic effect of a RAF/MEKi in MPNST cells. (**A**) Heatmap of synergy scores for cell death in JW23.3, JH-2-002, and WU-356 MPNST cells, generated by using SynergyFinder. Synergy score > 10 indicates synergy. (**B**) Defactinib enhanced cell death induction when used together with RAF/MEKi avutometinib, using YOYO-1 green fluorescence as an indicator of cell death. The cell death ratio was determined by calculating the number of green dead cells relative to cell confluence per well and normalizing this value to the baseline measurement at time 0. One-way ANOVA with Tukey’s post hoc test was used for multiple-group comparisons. (**C**) Representative bands of Western blot (Simple WES) assay for total or phosphorylated FAK, AKT, ERK1/2, and STAT3, as well as total or cleaved caspase-3 and PARP-1. GAPDH served as loading control. Avutometinib and defactinib were tested at indicated concentrations. Cell lysates were collected after 48 hours of treatment. WES assays were repeated twice; representative images are shown.

**Figure 6:**
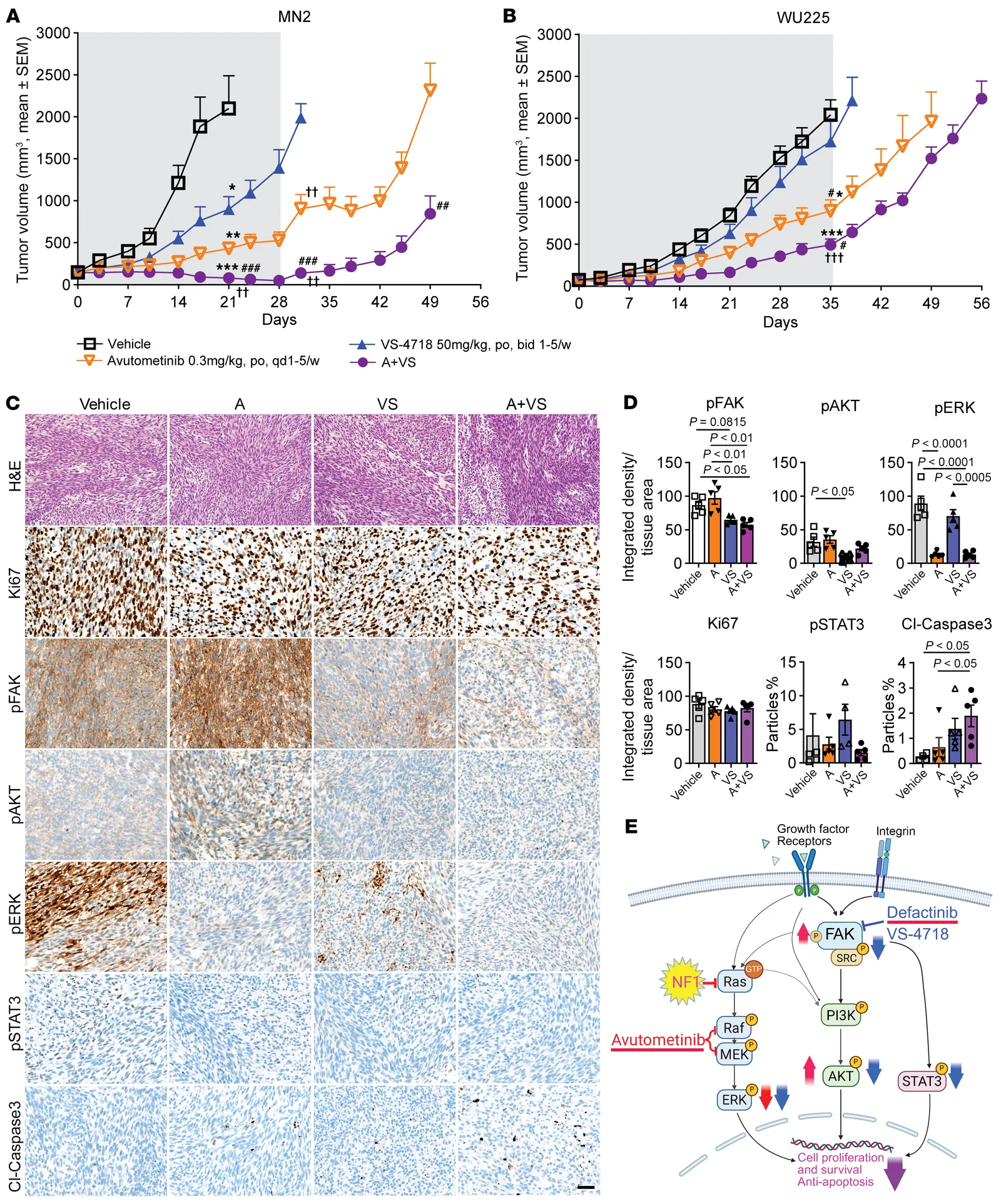
Combination of FAKi with RAF/MEKi decreases tumor growth in chr8 gain PDX mouse models. (**A**) MN2 MPNST PDX tumors (*n* = 6 per group); (**B**) WU-225 MPNST PDX tumors (*n* = 7 per group). Mice were treated once daily with 0.3 mg/kg avutometinib (A), twice daily with 50 mg/kg VS-4718 (VS), the combination of drugs, or vehicle control for 4–5 weeks. Gray area indicates treatment period. One-way ANOVA with Tukey’s post hoc test was used for multiple-group comparisons, and Student’s *t* test was used for comparisons between 2 groups. *P < 0.05, **P < 0.01, ***P < 0.001 vs. vehicle control; ^††^*P* < 0.01, ^†††^*P* < 0.001, for drug combination vs. VS-4718; ^#^*P* < 0.05, ^##^*P* < 0.01, ^###^*P* < 0.001 for drug combination vs. avutometinib. (**C**) Histological (H&E) and immunohistochemical (Ki67, p-FAK, p-AKT, p-ERK, p-STAT3, and cleaved caspase-3) staining of MN2 tumor tissues after 10 days of treatment. Scale bar, 50 μm. (**D**) In quantification of IHC staining, increased p-FAK and p-AKT were observed after treatment of avutometinib, while the combination with VS-4718 inhibited activity of both FAK and MEK pathways and induced apoptosis. One-way ANOVA with Tukey’s post hoc test was used for multiple-group comparisons, and Student’s *t* test was used for comparisons between 2 groups. (**E**) Mechanistic model to explain the combination benefit of FAK and RAF/MEK inhibition in NF1-MPNST with chr8 gain. The red arrow indicates the effect of the RAF/MEKi, the blue arrow shows the effect of the FAKi, and the purple arrow represents the combination effect. Created in BioRender. Hirbe, A. (2026) https://BioRender.com/aerpo6u po, orally; bid, twice daily; qd, once daily.

## Version 1 08/11/2026

In-Press Preview

## Version 2 10/01/2026

Electronic publication
