# Supplementary material for: Combined FAK and MEK inhibition suppresses chromosome 8 gain malignant peripheral nerve sheath tumors
Source: J Clin Invest. 2026 Aug 11;136(19):e201331. doi: 10.1172/JCI201331 (PMC13626845; doi:10.1172/JCI201331)
Supplement: Supplementary file 1 [file jci-136-201331-s007.pdf]

Supplementary Tables and Figures

Supplementary Table 1. 58 genes with *p* values <0.05 in CRISPR gRNA Screening. JH-2-002 human MPNST cell was used to knockdown the genes.

Gene	neg| p-value	Gene	neg| p-value
RPL8	4.95E-06	*ANK1*	0.014561
TOP1MT	4.45E-05	*ZFP41*	0.016134
ATP6V1B2	9.40E-05	*FAM86B1*	0.016332
RPS20	0.00011379	*POLB*	0.020221
IQANK1	0.0011132	*DEFB136*	0.022407
RAB2A	0.00092519	*KIFC2*	0.022605
POP1	0.0012319	*EXOSC4*	0.02405
MROH6	0.0012616	*GLI4*	0.024634
RHPN1	0.001796	*PTK2*	0.024723
MED30	0.0023006	*RPL30*	0.02686
DCTN6	0.0023105	*NKX2*	0.028067
RPL7	0.0023798	*GINS4*	0.028206
ZNF623	0.0042203	*VCPIP1*	0.028335
ASH2L	0.0045765	*DSCC1*	0.030452
UBE2W	0.0081783	*CHMP7*	0.031462
UTP23	0.0089007	*C8orf76*	0.032936
RAD21	0.0096428	*REEP4*	0.033005
FGFR1	0.0097318	*PABPC1*	0.034638
RRS1	0.010118	*MYC*	0.036666
AZIN1	0.010326	*EXT1*	0.036805
COPS5	0.010454	*INTS9*	0.037141
EIF3E	0.011553	*MCM4*	0.037676
ELP3	0.011859	*USP17L4*	0.04105
TERF1	0.012008	*PSD3*	0.041545
VPS28	0.012275	*PUF60*	0.042089
C8orf33	0.012601	*LETM2*	0.042871
NRBP2	0.014096	*UBR5*	0.043524
GPAA1	0.014115	*DKK4*	0.048481
MAFA	0.014531	*SDC2*	0.049767

Supplementary Table 2. Antiproliferation effect of FAK inhibitor defactinib and RAF/MEK inhibitor avutometinib in MPNST cells *in vitro*

			IC_{50}(μM, Mean±SEM)	
**Cell lines**	**NF1**	**Chr8**	**Defactinib**	**Avutometinib**
JW23.3	+/-	N/A*	1.758±0.082	29.277 ± 4.448
JH-2-002	-/-	Amp	2.030±0.351	17.503 ± 10.202
WU356	-/-	Amp	1.703±0.167	9.183 ± 1.477
JH-2-055-d	-/-	Diploid	>10	2.027 ± 0.012

* Not applicable. JW23.3 is a murine cell line; the genes are located on different chromosomes.

Supplementary Table 3. Sequence of shRNA

Name	SigmaAldrach #	Sequence
shRNA-LacZ		CGCTAAATACTGGCAGGCGTT
Murine shRNA-FAK #1	TRCN0000321963	CCTGGCATCTTTGATATTATA
Murine shRNA-FAK #3	TRCN0000023485	GCCTTAACAATGCGTCAGTTT
Human shRNA-FAK #5	TRCN0000121129	GCCCAGGTTTACTGAACTTAA
Human shRNA-FAK #6	TRCN0000121318	CCGATTGGAAACCAACATATA
Human shRNA-FAK #7	TRCN0000196310	GATGTTGGTTTAAAGCGATTT
Human shRNA-FAK #8	TRCN0000194984	CAACAGGTGAAGAGCGATTAT

Supplementary Table 4. Sequence of primers

Name	Sequence
Murine PTK2	Forward 5′-TTAGGCGATCCTATTGGGAGATG-3’
	Reverse 5′-TTCTTAGTGTTTTGGCCTTGACA-3’
Murine 18S rRNA	Forward 5′-GTAACCCGTTGAACCCCATT-3’
	Reverse 5′- CCATCCAATCGGTAGTAGCG-3’
Human PTK2	Forward 5′-GCACCCACCGAGAGATTGAG-3’
	Reverse 5′-GGGCCAGTTTCATCTTGTTGA-3’
Human 18S rRNA	Forward 5′-CTCAACACGGGAAACCTCAC-3’
	Reverse 5′-CGCTCCACCAACTAAGAACG-3’

Supplementary Table 5. Patients' information

cell lines/PDX	Patients' information
**JH-2-002**	OS 98.85 months, alive, Pazo; from resection of tumor after neoadjuvant pazopanib and radiation
**WU356**	OS 15 months, deceased, dox/ifos before PDX made
**MN2**	The information could not be obtained
**WU487**	OS 33.8 months, deceased, PDX made from untreated MPNST (patient did have chemo after surgery)
**WU-225**	OS 19.8 months, deceased, dox/ifos before PDX made
**JH-2-055**	OS 65.08 months, alive, AI-ifos/eto + VIT; resection of tumor after neoadjuvant ifosfamide, doxorubicin (two cycles) and ifos/ etoposide (one cycle), and neoadjuvant radiation, with extensive necrosis on path at resection.

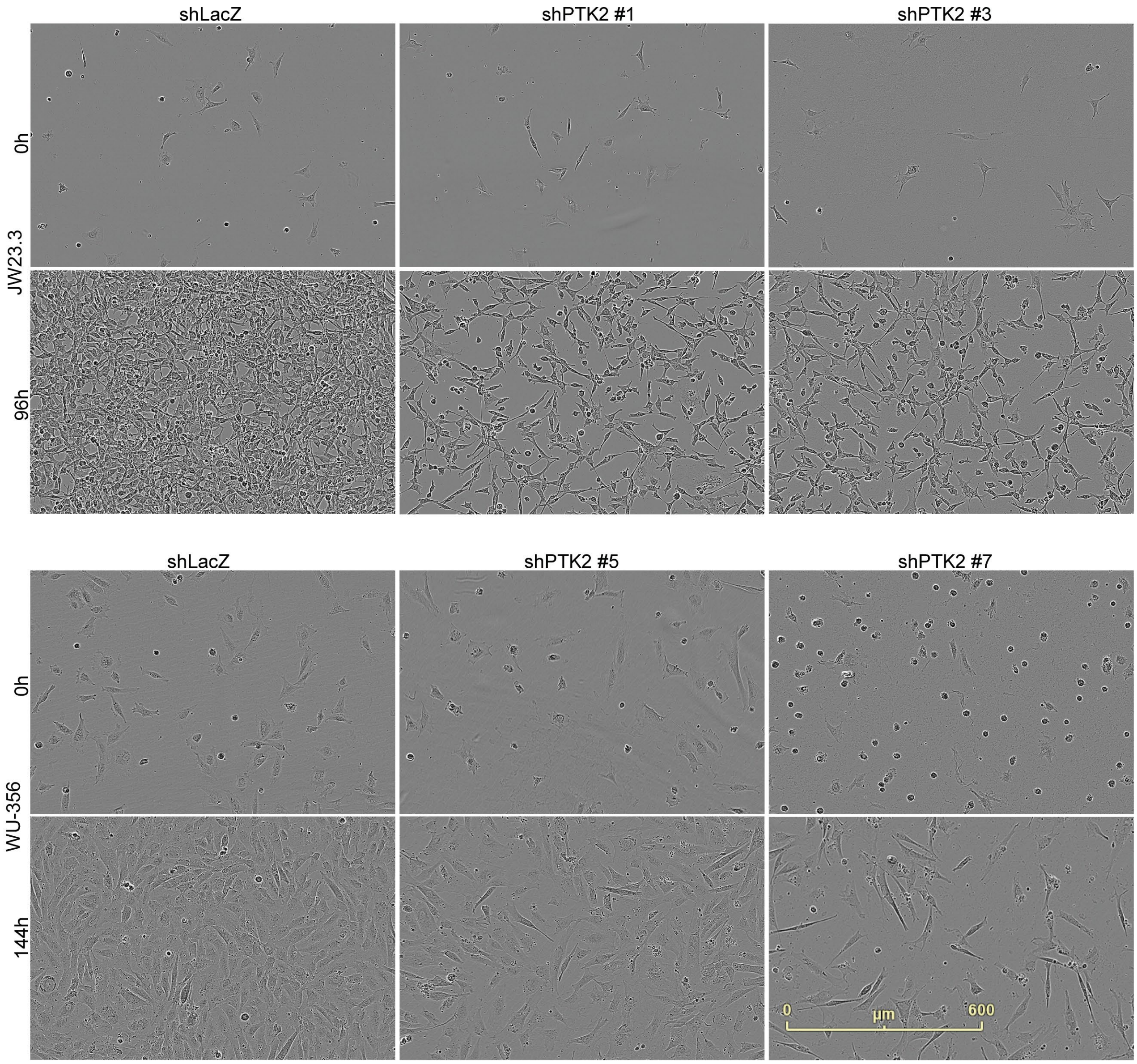

Supplementary Figure 1. Knockdown of *PTK2* inhibited cell proliferation of MPNST cells JW23.3 and WU-356. Representative images of IncuCyte assay for JW23.3 and WU-356 cells at indicated timepoints.

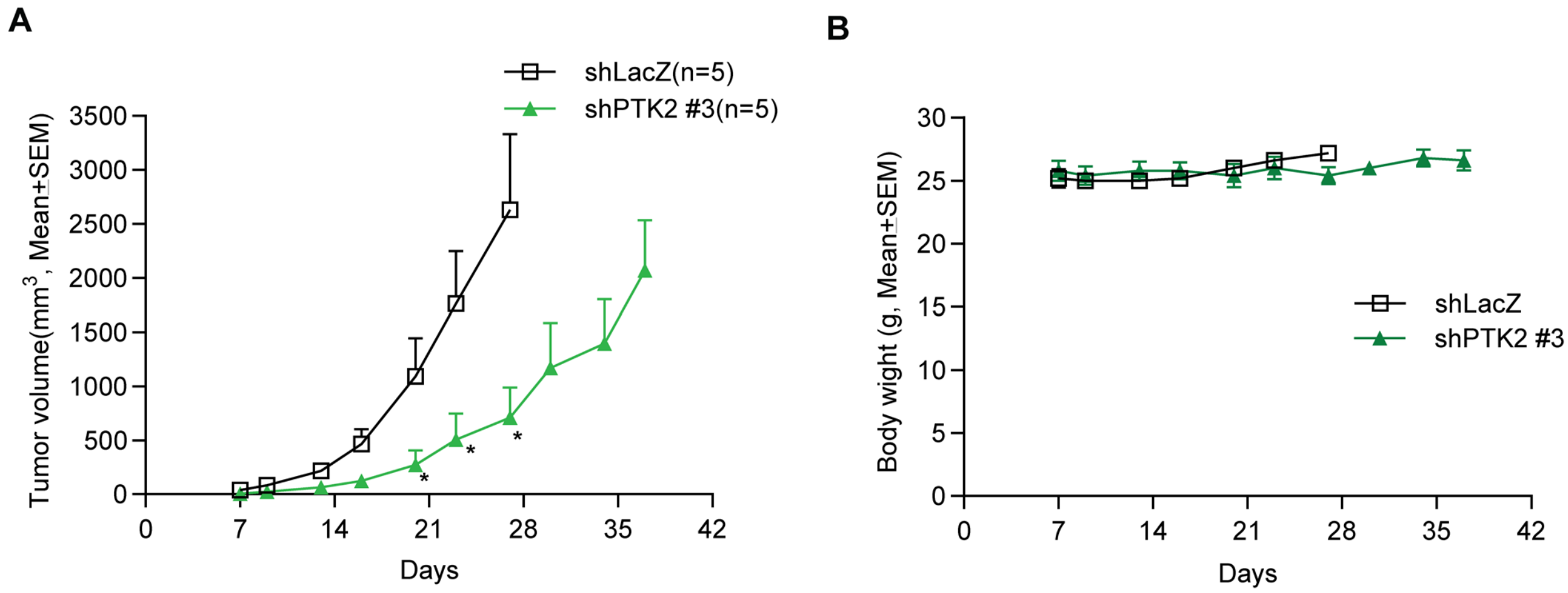

Supplementary Figure 2. PTK2 knockdown via shRNA reduces tumorigenesis in MPNST JW23.3 cells in vivo. (**A**) Tumor volume measurements over time in C57BL mice injected subcutaneously with 1×10⁶ JW23.3 cells, demonstrating reduced tumor growth in the *PTK2* knockdown group. Student's *t*-test was used for comparisons between two groups. *: $p<0.05$ vs. shLacZ. (**B**) Body weight monitoring of mice throughout the study, showing no significant differences between groups.

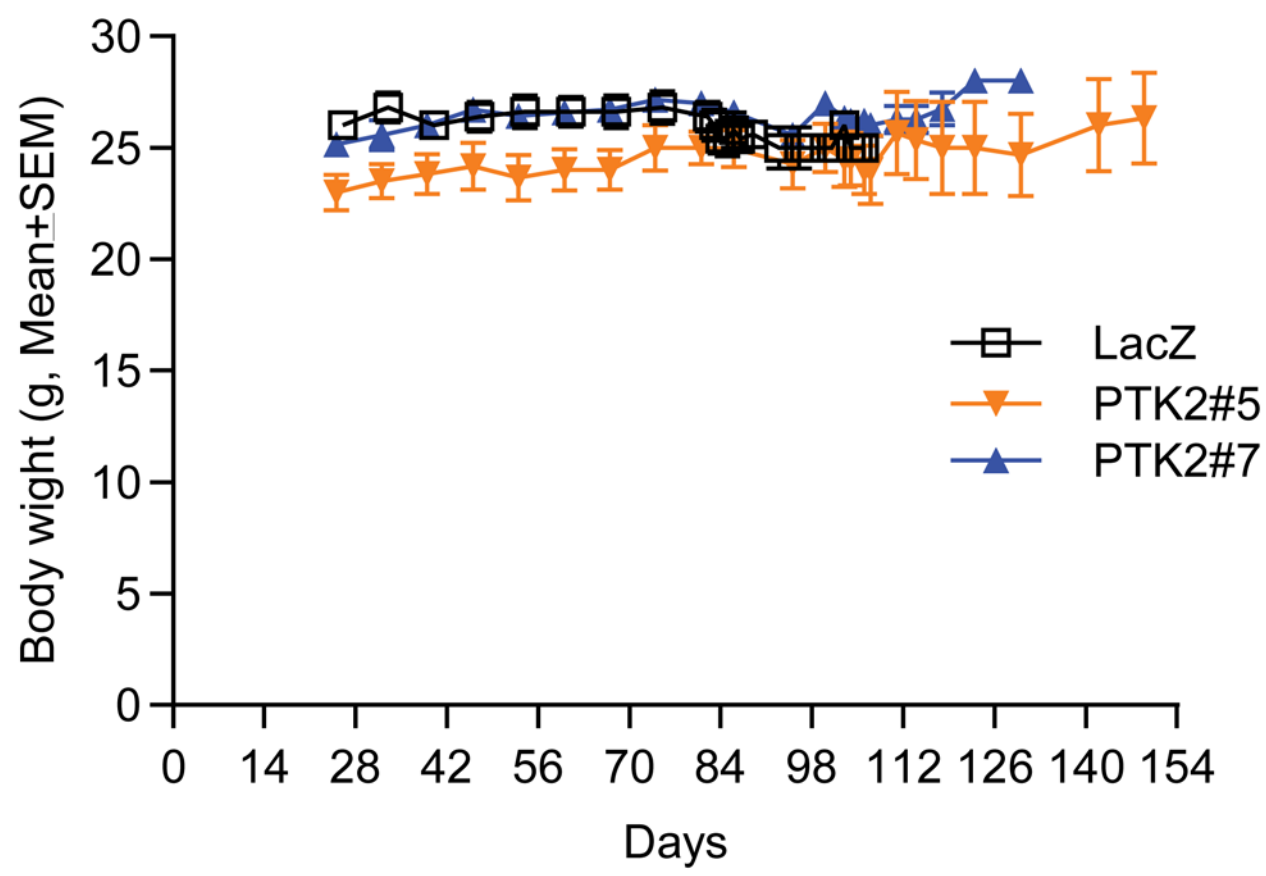

Supplementary Figure 3. Body weight of mice in the JH-2-002 orthotopic MPNST model with PTK2 knockdown via shRNA. Nude mice injected orthotopically with $1{\times}10^5$ JH-2-002 cells (5 µL) into the sciatic nerve, showing prolonged survival in the *PTK2* knockdown groups. Body weight monitoring throughout the study, indicating no significant adverse effects of *PTK2* knockdown.

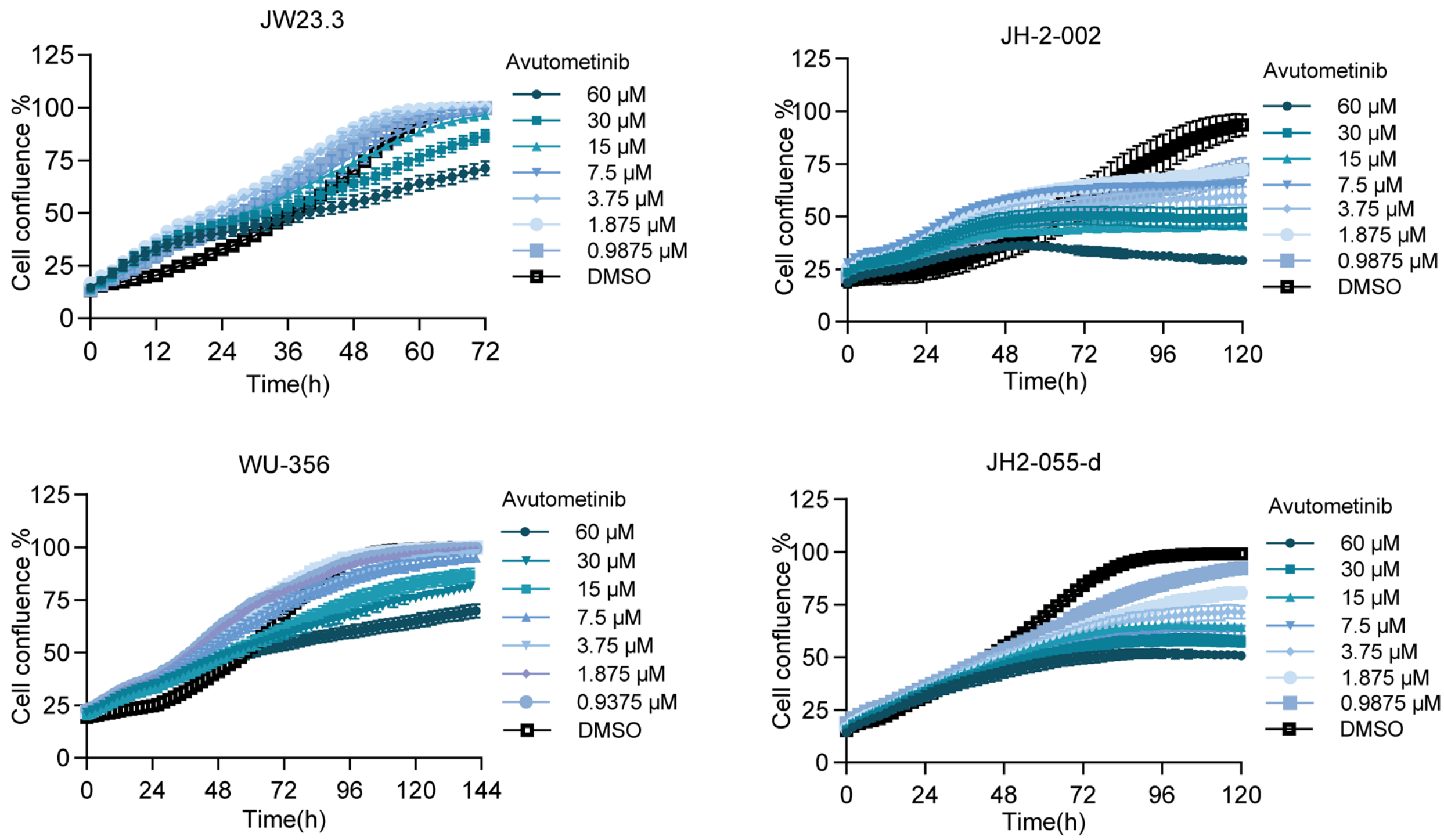

Supplementary Figure 4. Anti-proliferative effect of RAF/MEK inhibitor Avutometinib in MPNST cells *in vitro*. Real-time monitoring of MPNST cell proliferation under RAF/MEK inhibitor treatment using the Incucyte live-cell imaging system. Growth curves were generated by measuring confluence over time, showing the reduction in cell proliferation upon MEK inhibition.

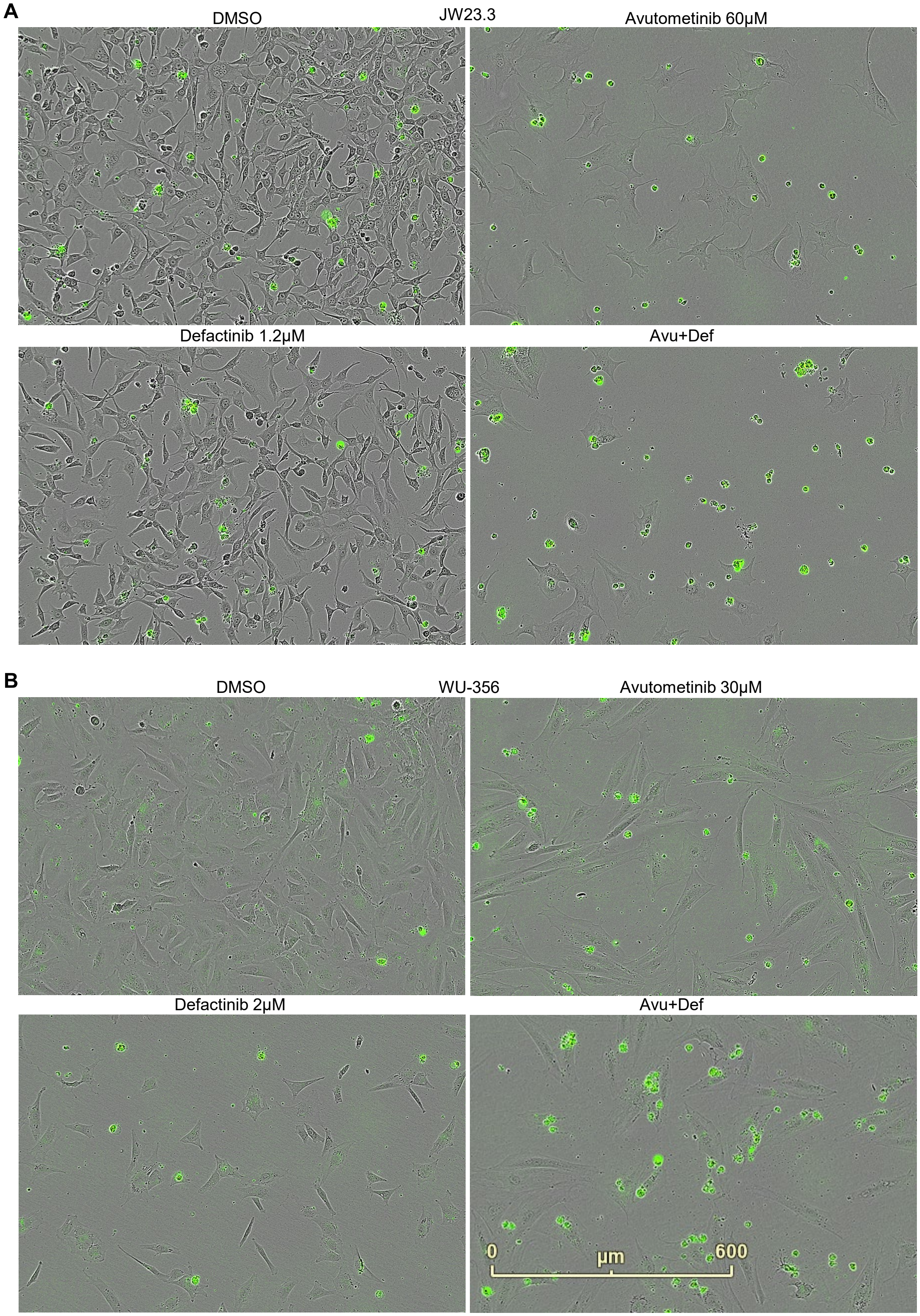

Supplementary Figure 5. The combination of a FAK inhibitor and a RAF/MEK inhibitor is synergistic in decreasing proliferation of MPNST cells. Representative images of IncuCyte assay at indicated timepoint, using YOYO-1 green fluorescent dye as an indicator of cell death. (**A**) JW23.3 (96h), (**B)** WU-356 (144h).

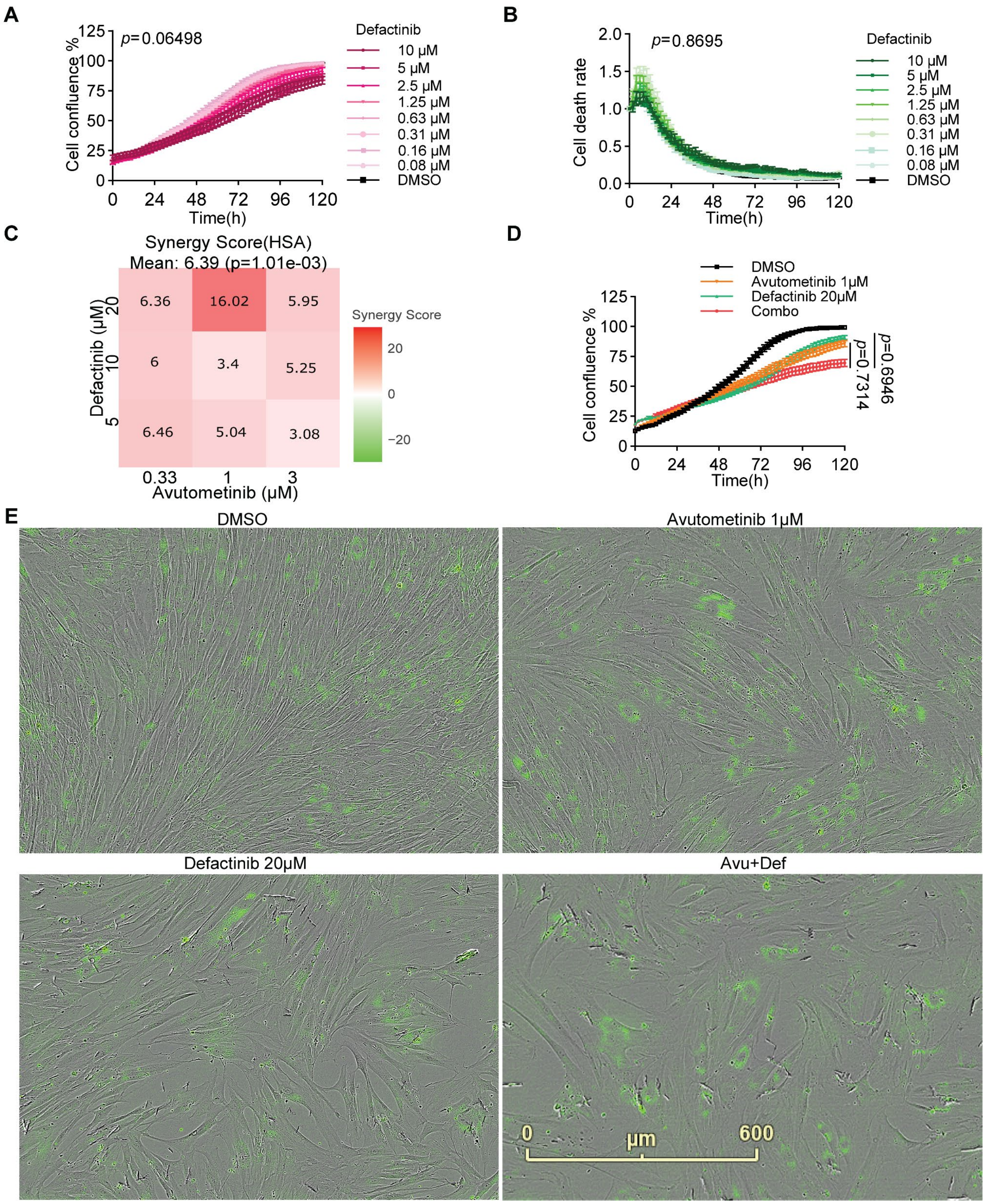

Supplementary Figure 6. Defactinib at higher concentration shows additive antiproliferation effect when used together with RAF/MEK inhibitor Avutometinib in human MPNST JH-2-055-d cells. (**A**) Cell proliferation after treatment of defactinib in JH-2-055-d cell lines. (**B**) Defactinib failed to induce cell death in JH-2-055-d cell line. Using YOYO-1 green fluorescence as an indicator of cell death. The cell death ratio was determined by calculating the number of dead cells relative to cell confluence and normalizing this value to the baseline measurement at time 0. and (**C**) Heatmap of Synergy Score generated by using SynergyFinder, cells were treated for 120h. Synergy Score: >10 indicates synergy; and (**D**) Representative curves for cell confluences over time in IncuCyte live cell assays. One-way ANOVA with Tukey's post hoc test was used for multiple-group comparisons; (**E**) Representative images of IncuCyte assay at 120h, using YOYO-1 green fluorescent dye as an indicator of cell death.

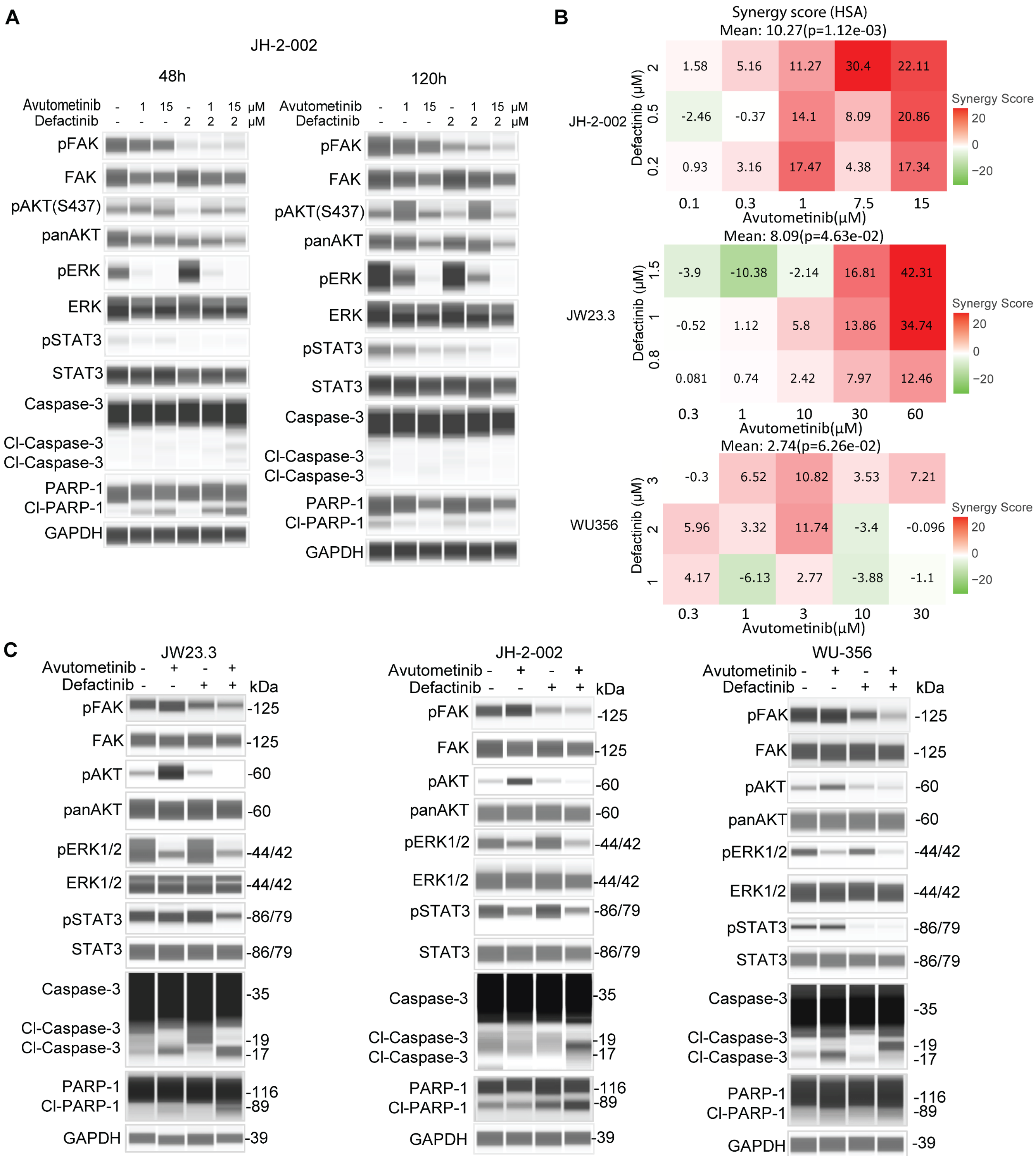

Supplementary Figure 7. FAK inhibitor enhances effect of RAF/MEK inhibitor in human MPNST cells. (**A**) Dose- and time-dependent molecular changes in JH-2-002 cells. Representative western blot (Simple WES) results are shown. (**B**) Heatmap of Synergy Scores for anti-proliferation effect of the FAK inhibitor Defactinib and the RAF/MEK inhibitor Avutometinib in JW23.3, JH-2-002, and WU-356 cells, generated by using SynergyFinder (www.synergyfinderplus.org). Cell confluence was monitored by using Incucyte system. Synergy Score: >10 indicates synergy, whereas scores between -10 and 10 indicate additive effects. (**C**)FAK inhibitor enhances apoptotic effect of RAF/MEK inhibitor in MPNST cells. Representative bands of western blot (SimpleWes) assay for total or phosphorylated FAK, AKT, ERK1/2, and STAT3, as well as total or cleaved Caspase-3 and PARP-1. GAPDH served as loading control. Avutometinib was tested at 500nM for JW23.3 and 50nM for JH-2-002 and WU356, Defactinib was tested at 200nM. Cell lysates were collected after 48 hours of treatment. WES assays were repeated twice; representative images are shown.

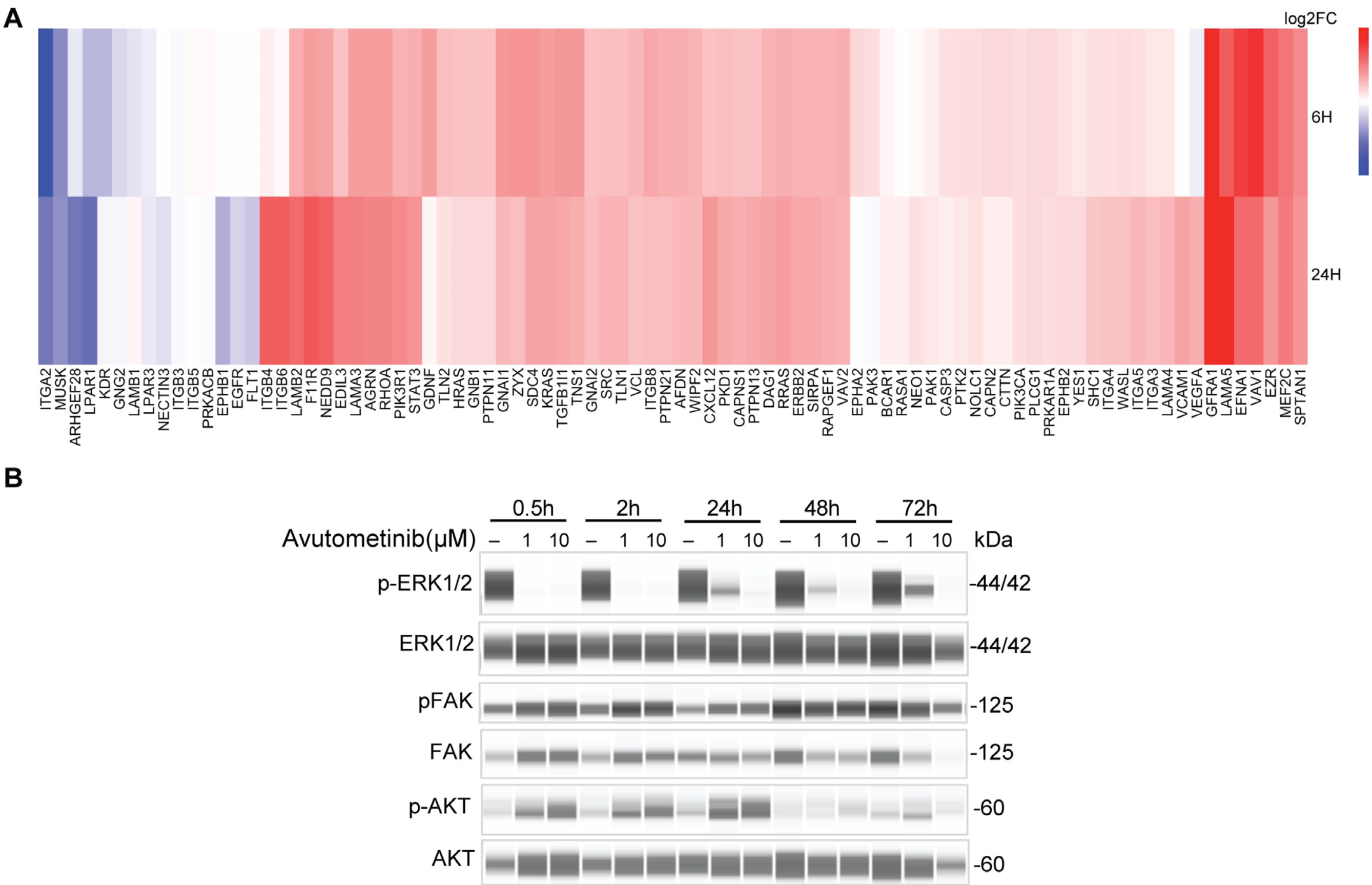

Supplementary Figure 8. MEK inhibitors treatment induce upregulation of FAK pathway in MPNST cells. (**A**) Heatmap showing expression changes of FAK-related genes following MEK inhibitor (trametinib) treatment at 6 or 24 hours**.** Differentially expressed gene (DEG) analysis following trametinib treatment in the JH-2-002 cell line is obtained from Synapse (accession: syn35589856). The list of FAK-related genes is curated from a previous study (34). Genes with an adjusted p-value (adj. p) < 0.05 were considered significantly differentially expressed. A total of 196 FAK-related genes were analyzed; among them, 86 genes were significantly regulated ($p < 0.05$, regardless of $\log_2$ fold change). Genes such as *PTK2B*, *RHOA*, *STAT3* and *R-RAS* are upregulated. Src family kinases *FYN* and *LCK* are also upregulated. Additionally, several integrins and laminin-related proteins show increased expression. (**B**) Western blot analysis showing increased phosphorylation levels of FAK (pFAK) and AKT (pAKT) following RAF/MEK inhibitor avutometinib treatment in MPNST JH-2-002 cells, suggesting activation of compensatory survival pathways.

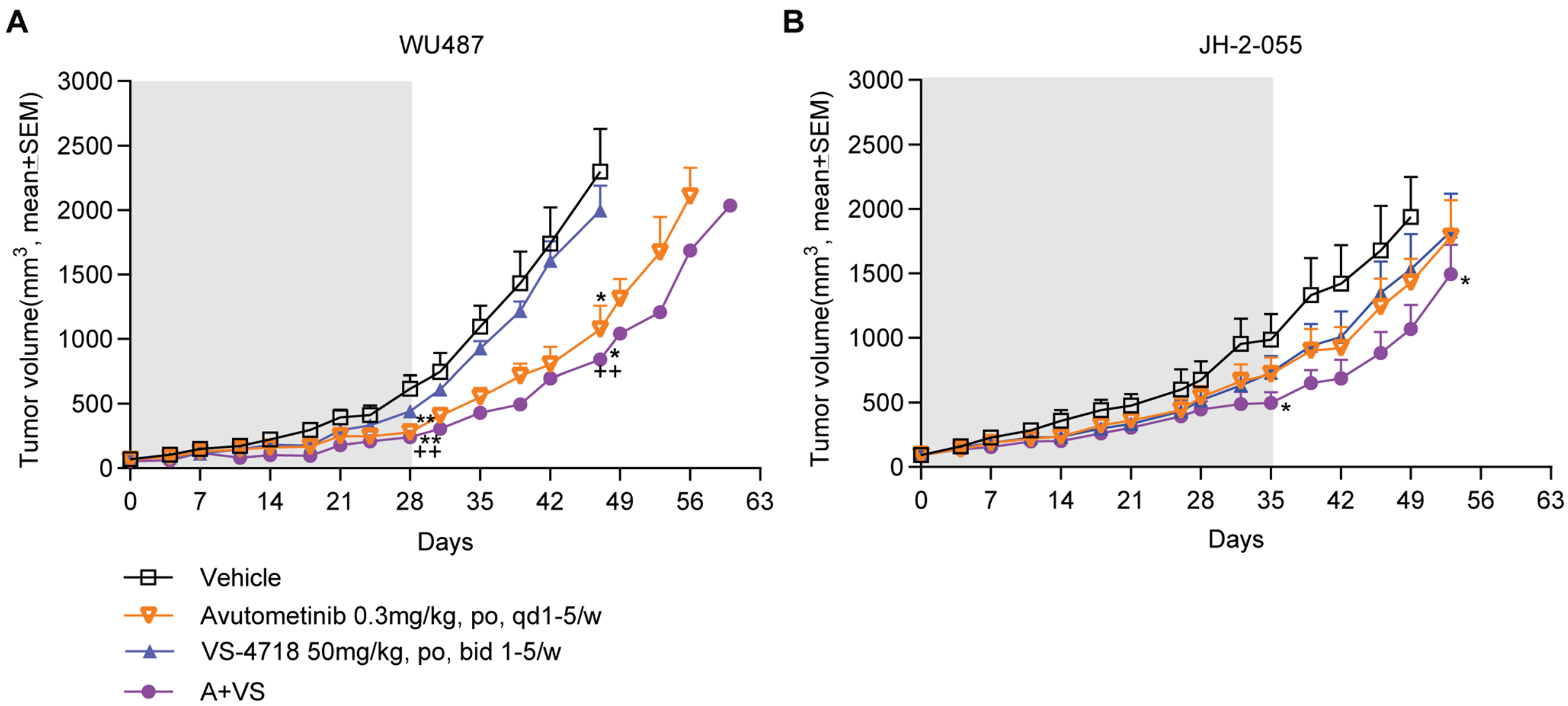

Supplementary Figure 9. **Combination of FAKi with MEKi in chromosome 8 diploid PDX mouse models.** (**A**) WU487 MPNST PDX tumors(n = 7 for vehicle control and n = 5 for treatment groups); and (**B**) JH-2-055 MPNST PDX tumors (n = 8 per group). Mice were treated once daily with 0.3 mg/kg avutometinib(A), twice daily with 50 mg/kg VS-4718(VS), the combination of drugs, or vehicle control for 4-5 weeks. Grey area indicates treatment period. One-way ANOVA with Tukey's post hoc test was used for multiple-group comparisons, and Student's *t*-test was used for comparisons between two groups. *: $p < 0.05$, **: $p<0.01$ vs. vehicle control; ++: $p<0.01$, for drug combination vs. VS-4718.

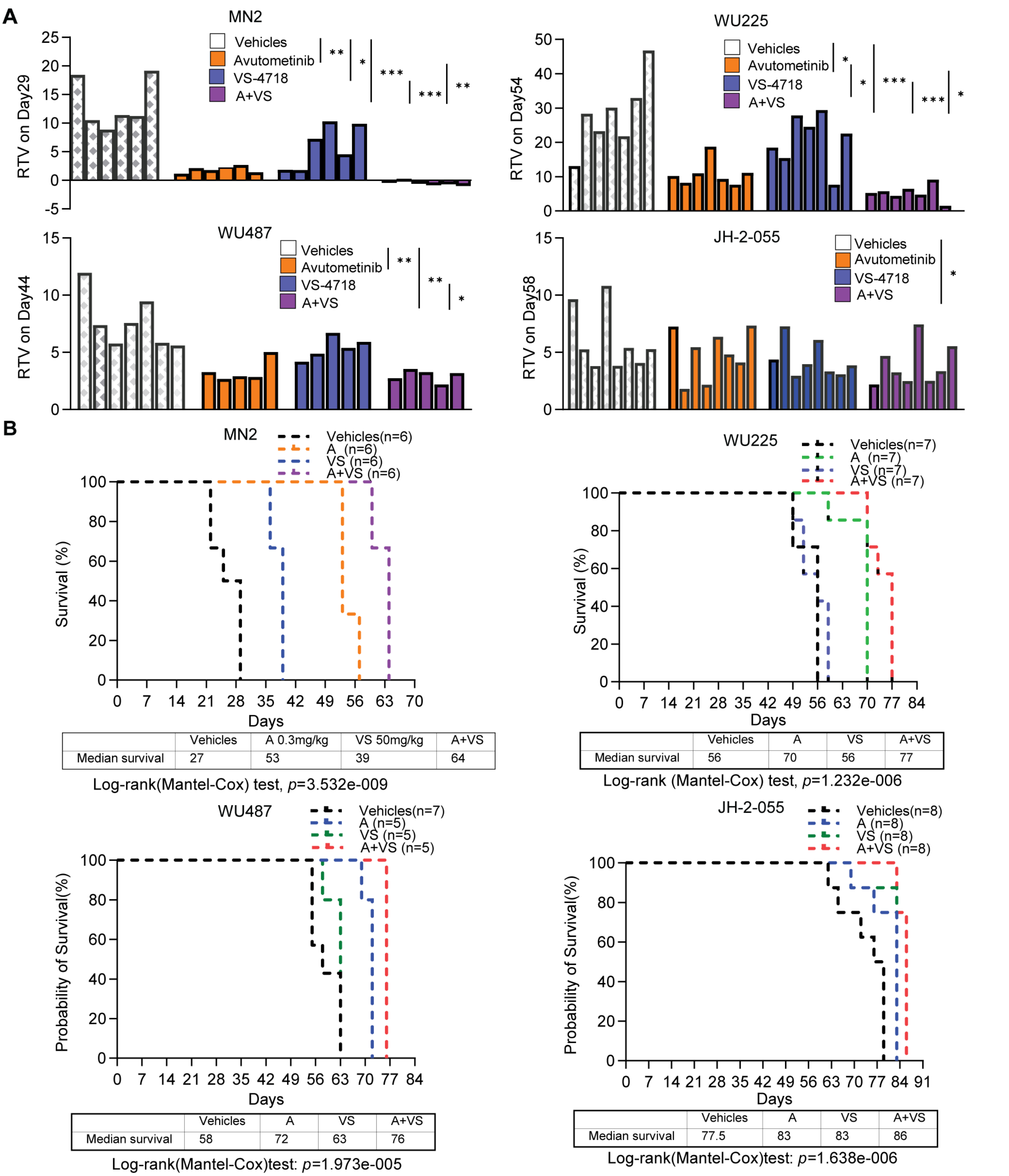

Supplementary Figure 10. Survival proportion of FAKi in combination with MEKi in chromosome 8 gain-associated PDX models and chromosome 8 diploid models.

(**A**) Waterfall plot for relative tumor volume (RTV) at the end of treatment. RTV was calculated as $(TVt - TV_0) / TV_0$, where TVt represents tumor volume at the indicated timepoint and TV_0 represents baseline tumor volume at Day 0. One-way ANOVA with Tukey's post hoc test was used for multiple-group comparisons. *: $p < 0.05$, **: $p<0.01$, ***: $p<0.001$; (**B**) Survival proportion. Endpoint defined as tumor volume reaching 1800 - 2000 mm³. Mice were treated once daily with 0.3 mg/kg avutometinib(A), twice daily with 50 mg/kg VS-4718(VS), the combination of drugs, or vehicle control for 4-5 weeks. Survival analyses were conducted using the Kaplan-Meier method, and survival distributions were compared with the log-rank (Mantel-Cox) test.

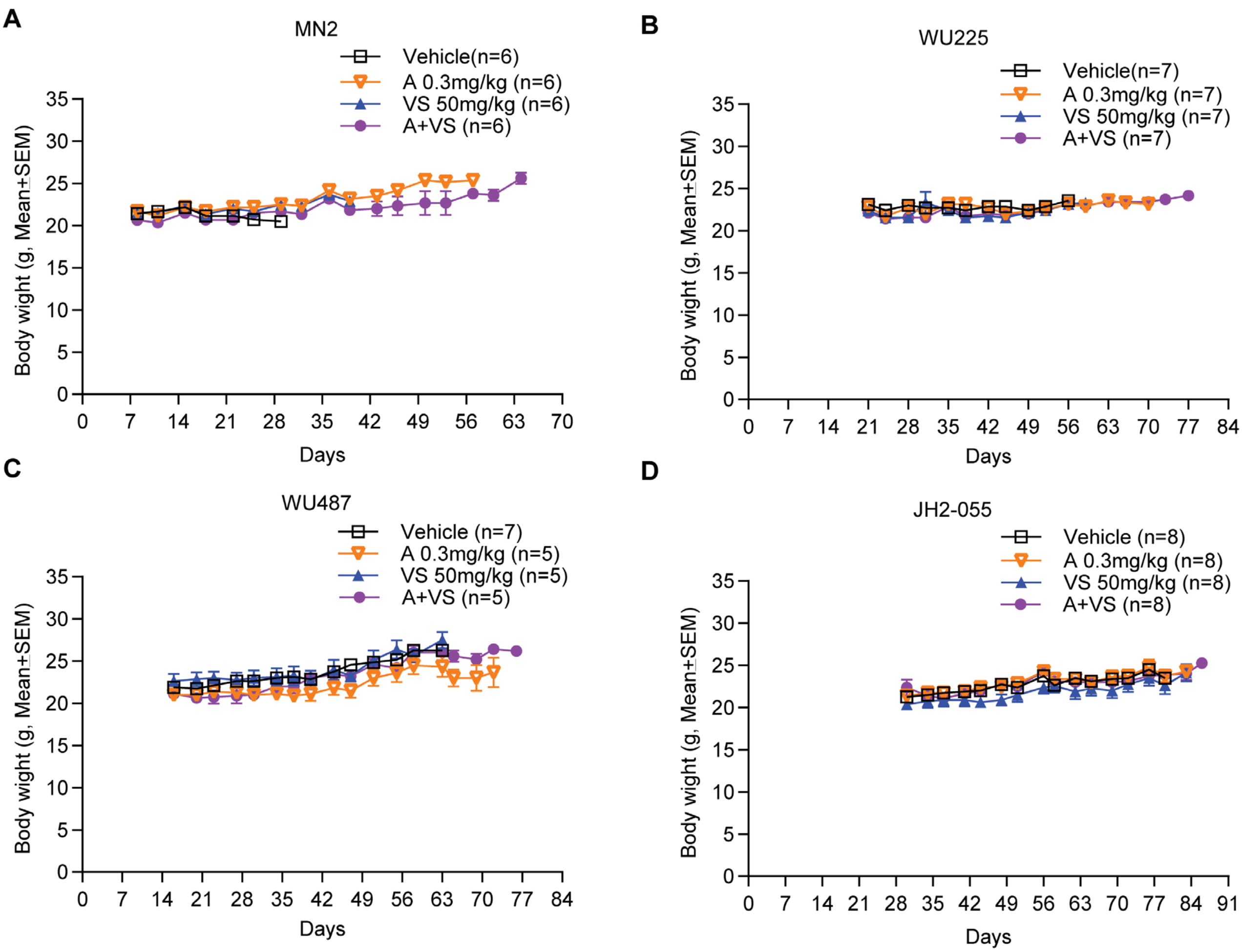

Supplementary Figure 11. **Body weight of FAKi in combination with MEKi in chromosome 8 gain-associated PDX models and chromosome 8 diploid models.**

(**A**) MN2 MPNST xenograft tumors; (**B**) WU-225 MPNST PDX tumors; (**C**) WU487 MPNST xenograft; and (**D**) JH-2-055 MPNST xenograft tumors. Mice were treated once daily with 0.3 mg/kg avutometinib (A), twice daily with 50 mg/kg VS-4718 (VS), the combination of drugs, or vehicle control for 4-5 weeks.

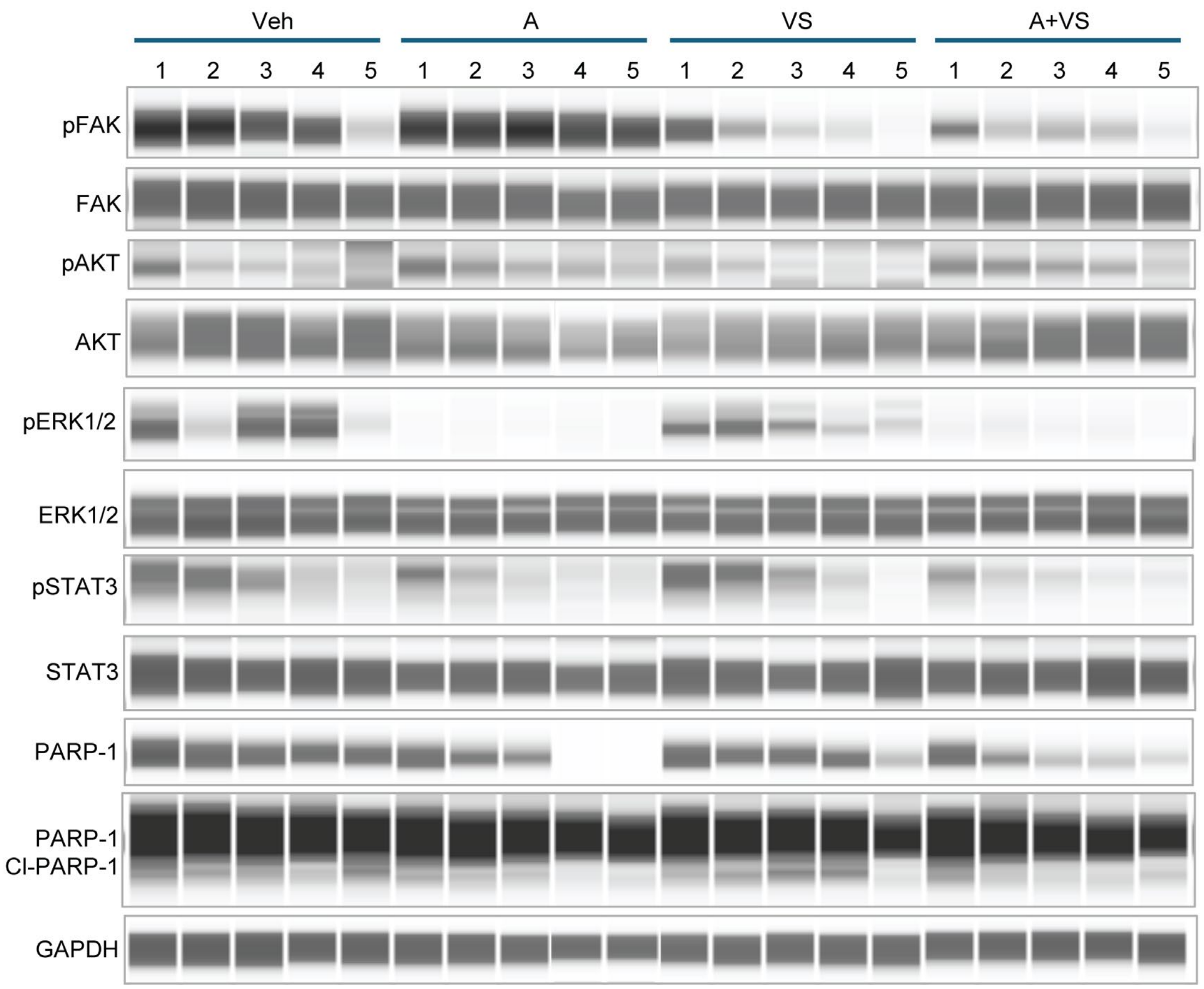

Supplementary Figure 12. FAK inhibitor enhanced effect of RAF/MEK inhibitor in MPNST PDX MN2 tumor. Representative bands of western blot (SimpleWes) assay for total or phosphorylated FAK, AKT, ERK1/2, and STAT3, as well as total or cleaved PARP-1. GAPDH served as loading control. VS-4718 (50mg/kg) was administered twice daily and avutometinib (1.5mg/kg) was administered once daily for 10 days. Tumor tissues were collected at 2 hours after the last treatment.

Supplementary Figure 13

Methods

Cell proliferation assay

ST8814 and NF90.8 cell lines were obtained from Dr. Gregory Riggins (Johns Hopkins University; Baltimore, MD, USA). Cells were cultured in Dulbecco's Modified Eagle Medium (DMEM, #10-013-CM, Corning, USA) supplemented with 10% FBS (Gibco, USA). Trametinib-resistant cells are maintained in complete cell culture medium with 20 nmol/L trametinib. 1000 cells per well were seeded in 96-well plates. A dose range of the compounds indicated was prepared by serial dilutions and then added to the wells containing adherent cells. Cells were incubated with drugs for five days. Cell growth was quantitated using the Cell Counting Kit-8 (Dojindo; Rockville, MD, USA) assay. For each cell line, two to four biological replicates with four (Fig S13A) and eight (Fig S13C) technical replicates of each condition were measured. Relative survival in the presence of drugs was normalized to the untreated controls after background subtraction and plotted as a function of log10 transformed drug doses or as bar graphs. The model Sigmoidal dose-response was used to perform the non-linear curve fitting and interpolate the IC50 values using the GraphPad Prism 11.

Colony formation assay

5000 cells/ well in 12-well plates were treated with DMSO, avutometinib (200 and 400 nmol/L), and/or defactinib (3 μmol/L) for 7 days. Cells were washed with PBS, fixed with 10% neutral buffered formalin (NBF), and then stained with 0.1% crystal violet for 30 minutes.

Immunoblotting

Cells were disrupted on ice in radioimmunoprecipitation assay lysis buffer. Protein concentration was determined with Pierce BCA (bicinchoninic acid) protein assay kit (Thermo Fisher Scientific, no. 23227). Equal amounts of protein were separated by SDS-polyacrylamide gel electrophoresis, transferred to nitrocellulose membranes, immunoblotted with specific primary and secondary antibodies, and detected by chemiluminescence with the ECL (electrochemiluminescence) detection reagents, Immobilon Western chemiluminescent horseradish peroxidase (HRP) substrate (MilliporeSigma, no. WBKLS0500). The membranes were imaged using ChemiDoc touch imaging system (Bio-Rad).
All antibodies p-FAK Y397(#3283), FAK (#3285), p-MEK1/2 S217/221 (#9121), p-ERK1/2 T202/Y204 (#9101), ERK1/2 (#9107), p-S6 S235/236 (#4858), CyclinD1 (#2978), GAPDH (#2118), beta-Actin (#4970) and Anti-rabbit IgG, HRP-linked Antibody (#7074) were purchased from Cell Signaling Technology.

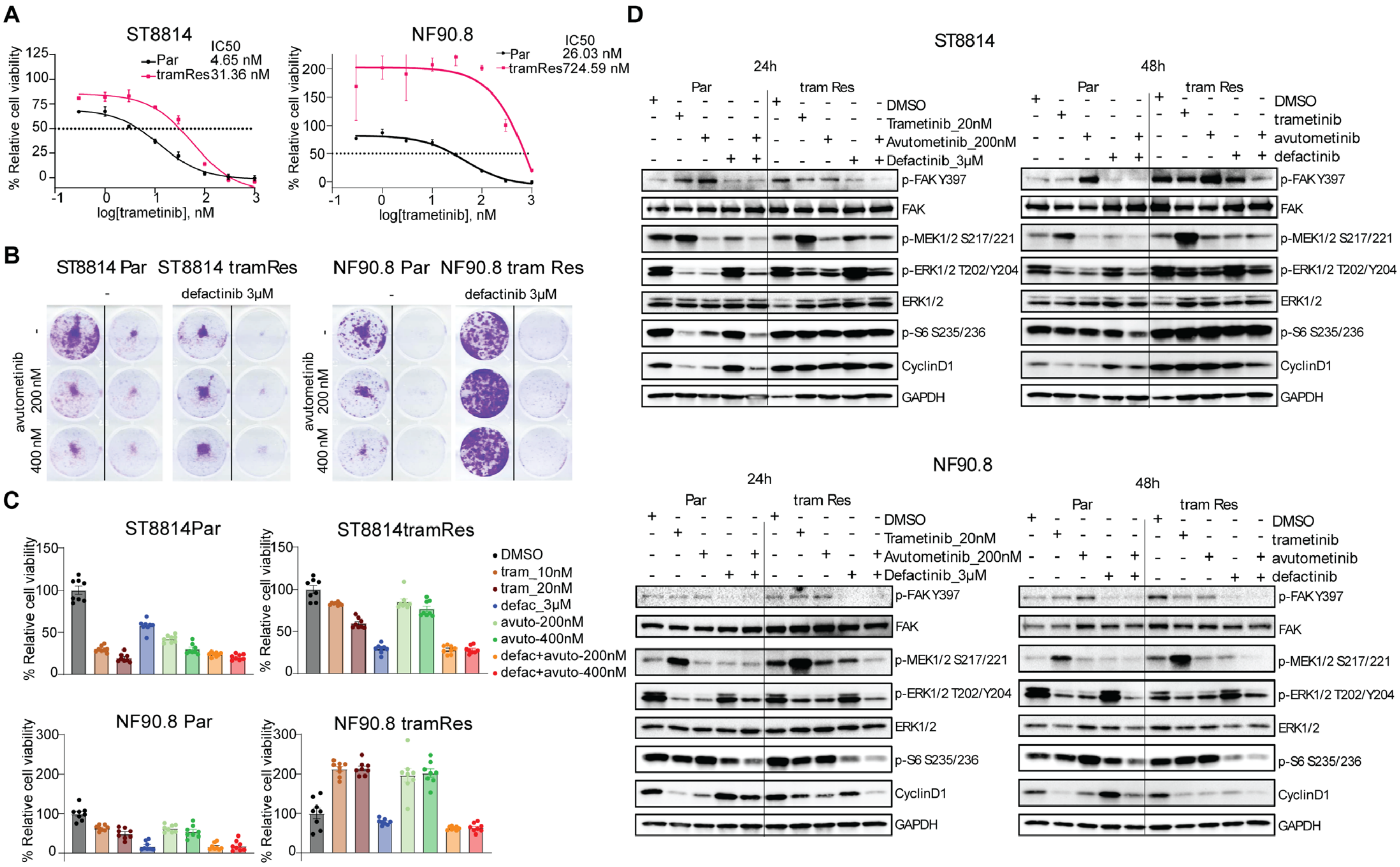

Supplementary Figure 13. Combination treatment with the FAK inhibitor defactinib and the RAF/MEK inhibitor avutometinib is effective in trametinib-resistant MPNST cell line models. (**A**) Generation of trametinib-resistant chr8 diploid MPNST cell line ST8814 and chr8 gain MPNST cell line NF90.8. (**B**) Defactinib, either as a single agent or in combination with avutometinib, inhibited colony formation in the paired parental and trametinib-resistant cell lines. (**C**) Defactinib alone or in combination with avutometinib suppressed cell proliferation in trametinib-resistant cells. (**D**) Western blot analysis demonstrated that defactinib effectively inhibited FAK phosphorylation and enhanced the effects of avutometinib in trametinib-resistant cells.
