# Supplementary material for: Combined FAK and MEK inhibition suppresses chromosome 8 gain malignant peripheral nerve sheath tumors
Source: J Clin Invest. 2026 Aug 11;136(19):e201331. doi: 10.1172/JCI201331 (PMC13626845; doi:10.1172/JCI201331)

Primary antibodies including rabbit anti Phospho-FAK (Tyr397) (#3283), FAK (#3285), Phospho-p44/42 MAPK (Erk1/2) (Thr202/Tyr204) (#4370), p44/42 MAPK (Erk1/2) (#4695), Phospho-Stat3 (Tyr705) (D3A7) (#9145), STAT3 (#4904), Phospho-Akt (Ser473) (#4060), Akt(pan)(#4691), p-ERK1/2 T202/Y204 (#9101), ERK1/2 (#9107), p-MEK1/2 S217/221 (#9121), p-S6 S235/236 (#4858), CyclinD1 (#2978), GAPDH (#2118), Caspase-3 (#9662), PARP (#9542), and β-Actin (#4967 and #4970) antibodies were purchased from Cell Signaling Technology. Primary rabbit anti-GAPDH (#TAB1001, Invitrogen) antibody was purchased from Thermo Fisher Scientific Inc. Anti-Rabbit Detection Module(#DM-001, Bio-Techne, Minnesota, USA) was used to detect the targeted primary antibodies.

Figure 1E

Raw blots

FAK

Panel 1

Panel 2

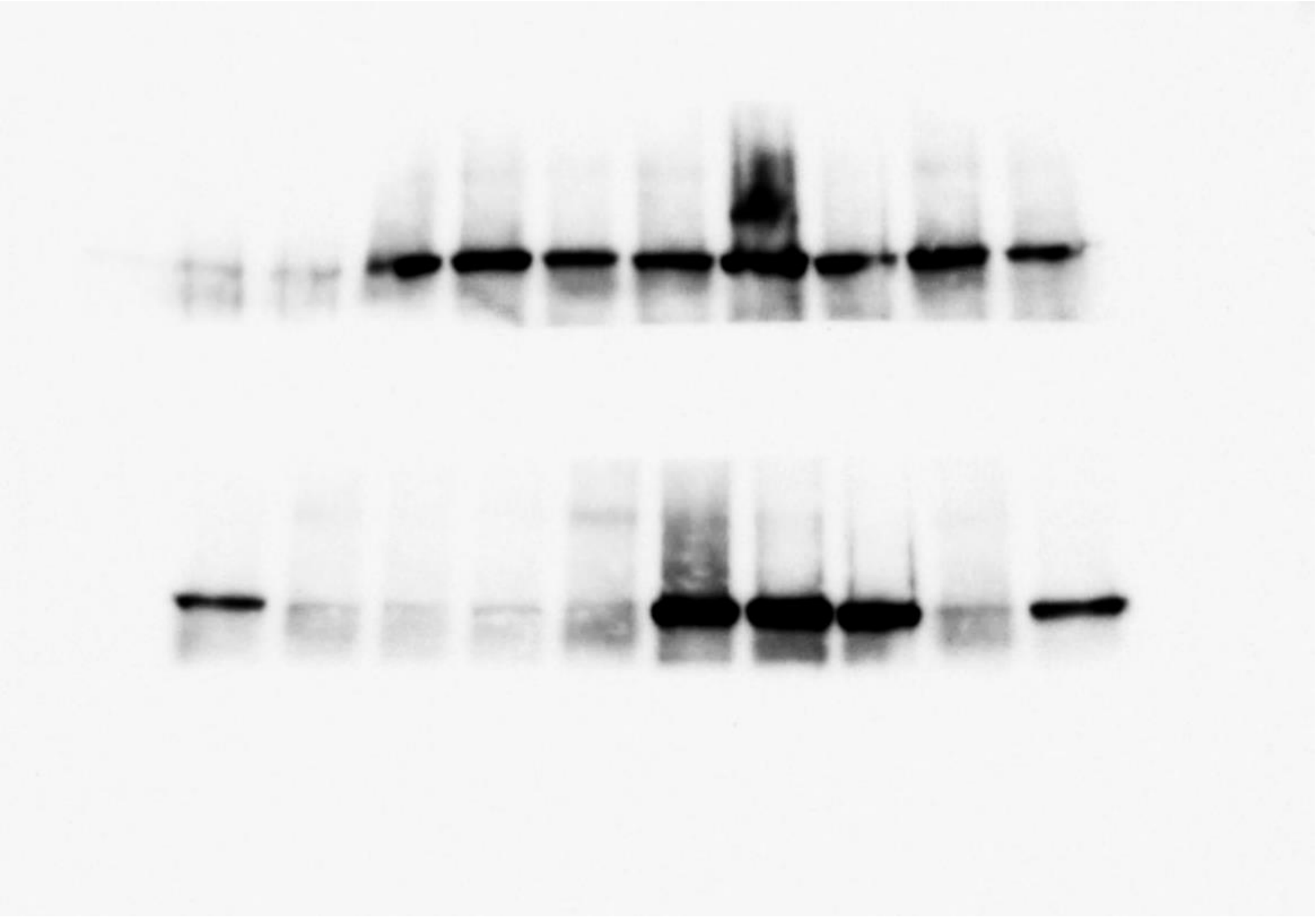

beta-Actin

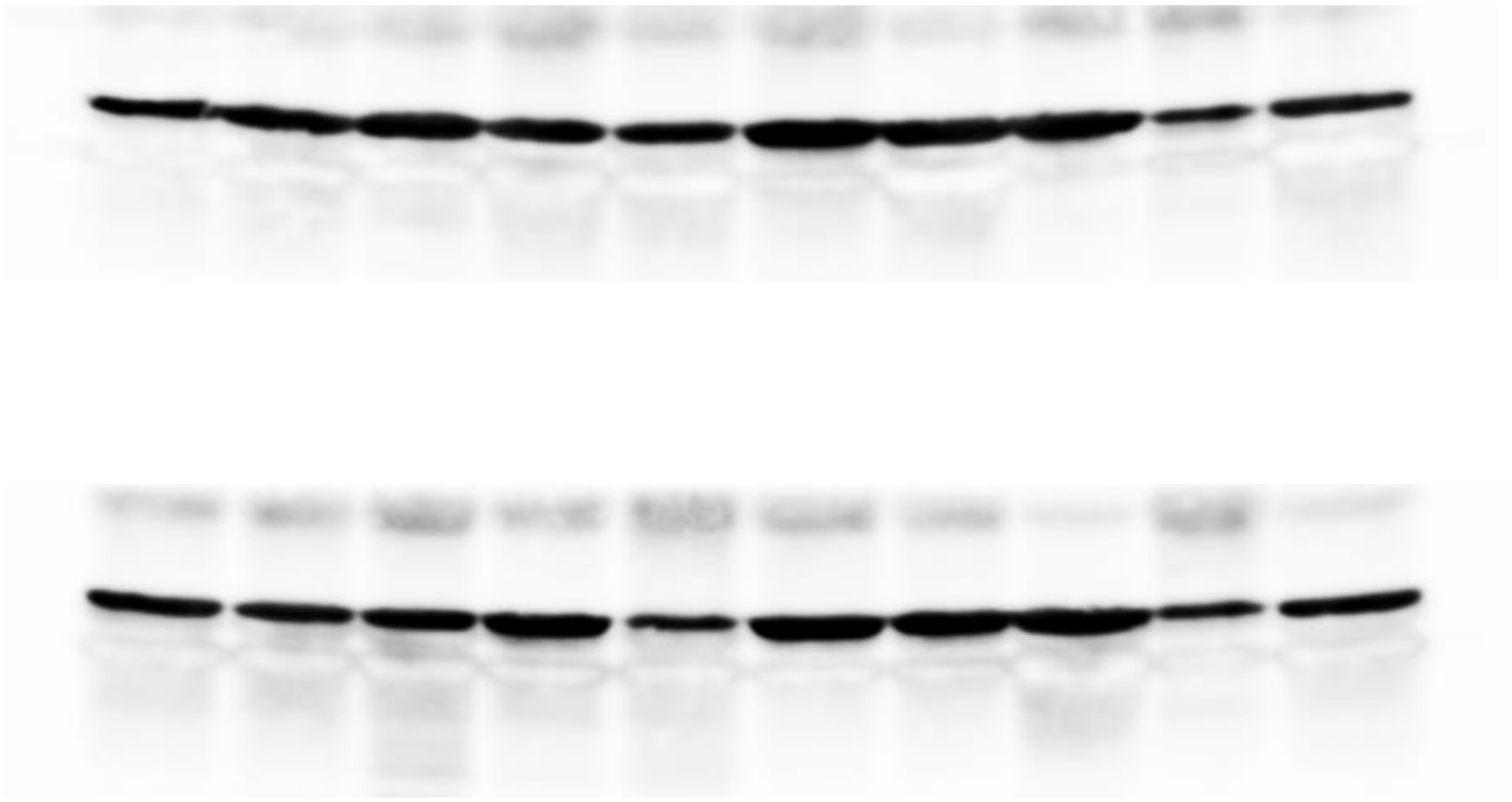

Figure 2B

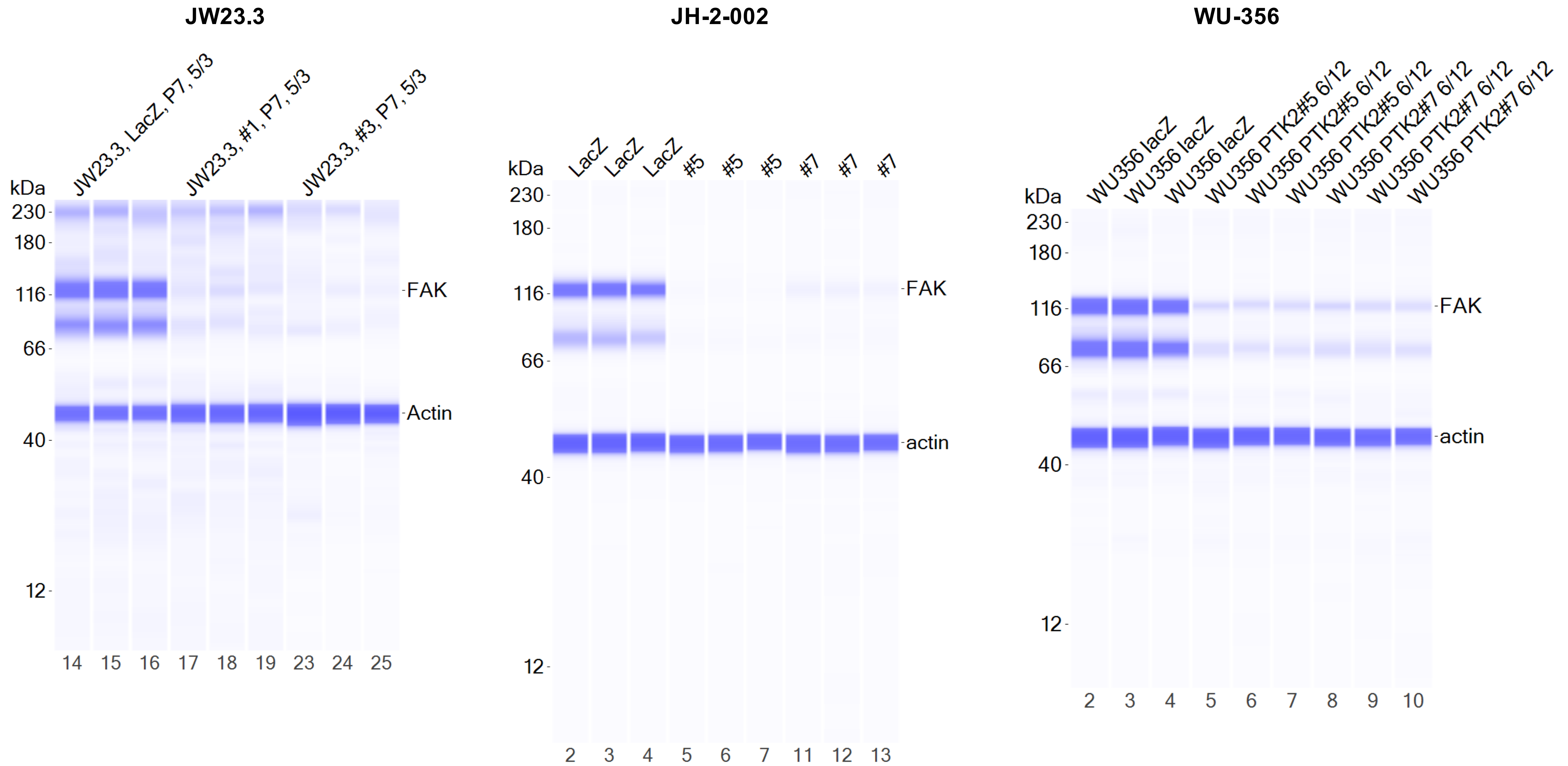

JW23.3
JW23.3, LacZ, P7, 5/3
JW23.3, #1, P7, 5/3
JW23.3, #3, P7, 5/3
kDa
230
180
116
66
40
12
FAK
Actin
14 15 16 17 18 19 23 24 25
JH-2-002
LacZ LacZ LacZ #5 #5 #5 #7 #7 #7
kDa
230
180
116
66
40
12
FAK
actin
2 3 4 5 6 7 11 12 13
WU-356
WU356 lacZ
WU356 lacZ
WU356 lacZ
WU356 PTK2#5 6/12
WU356 PTK2#5 6/12
WU356 PTK2#5 6/12
WU356 PTK2#7 6/12
WU356 PTK2#7 6/12
WU356 PTK2#7 6/12
kDa
230
180
116
66
40
12
FAK
actin
2 3 4 5 6 7 8 9 10

Figure 3C

JW23.3

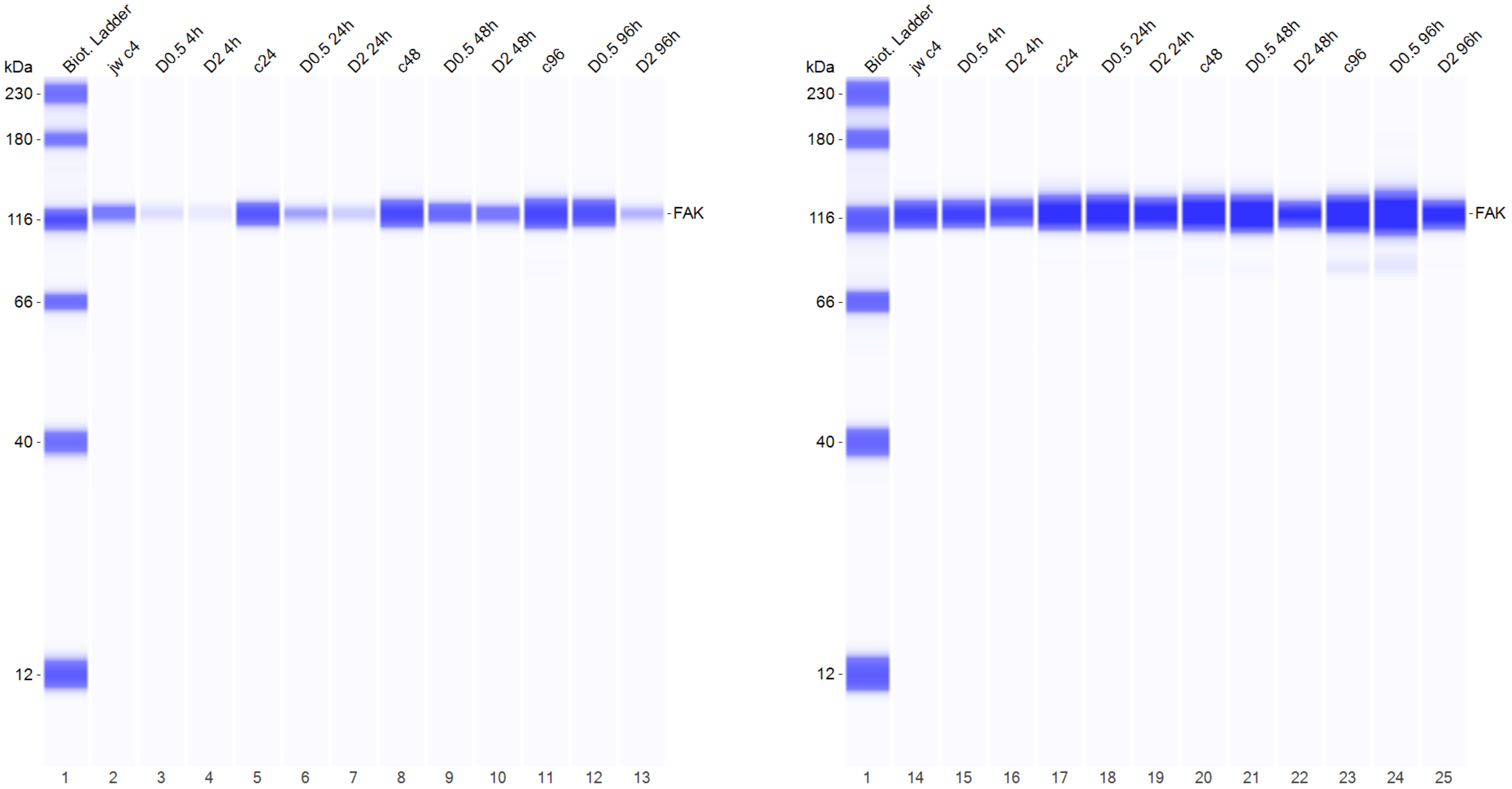

Figure 3C

JH-2-002

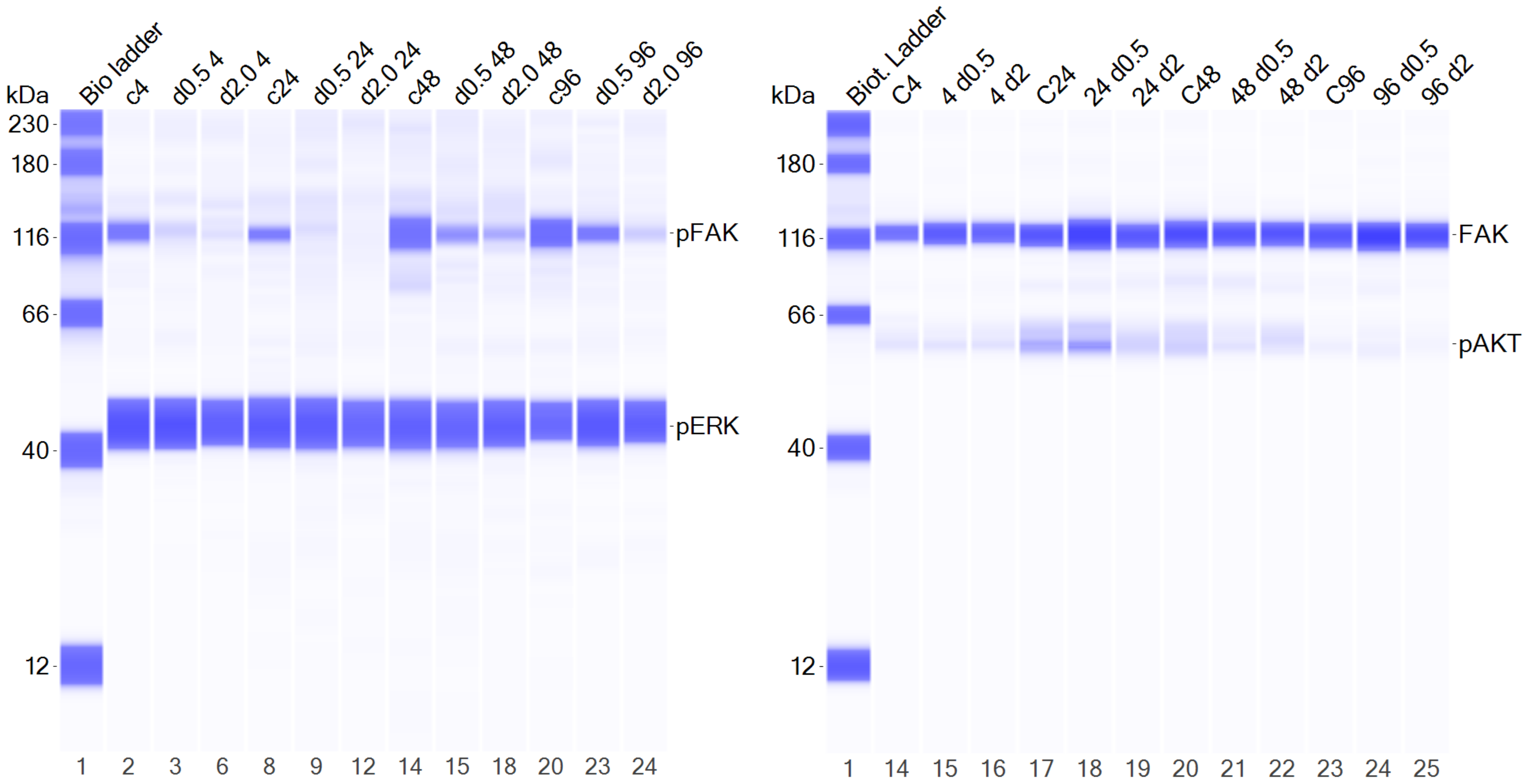

Figure 3C

WU356

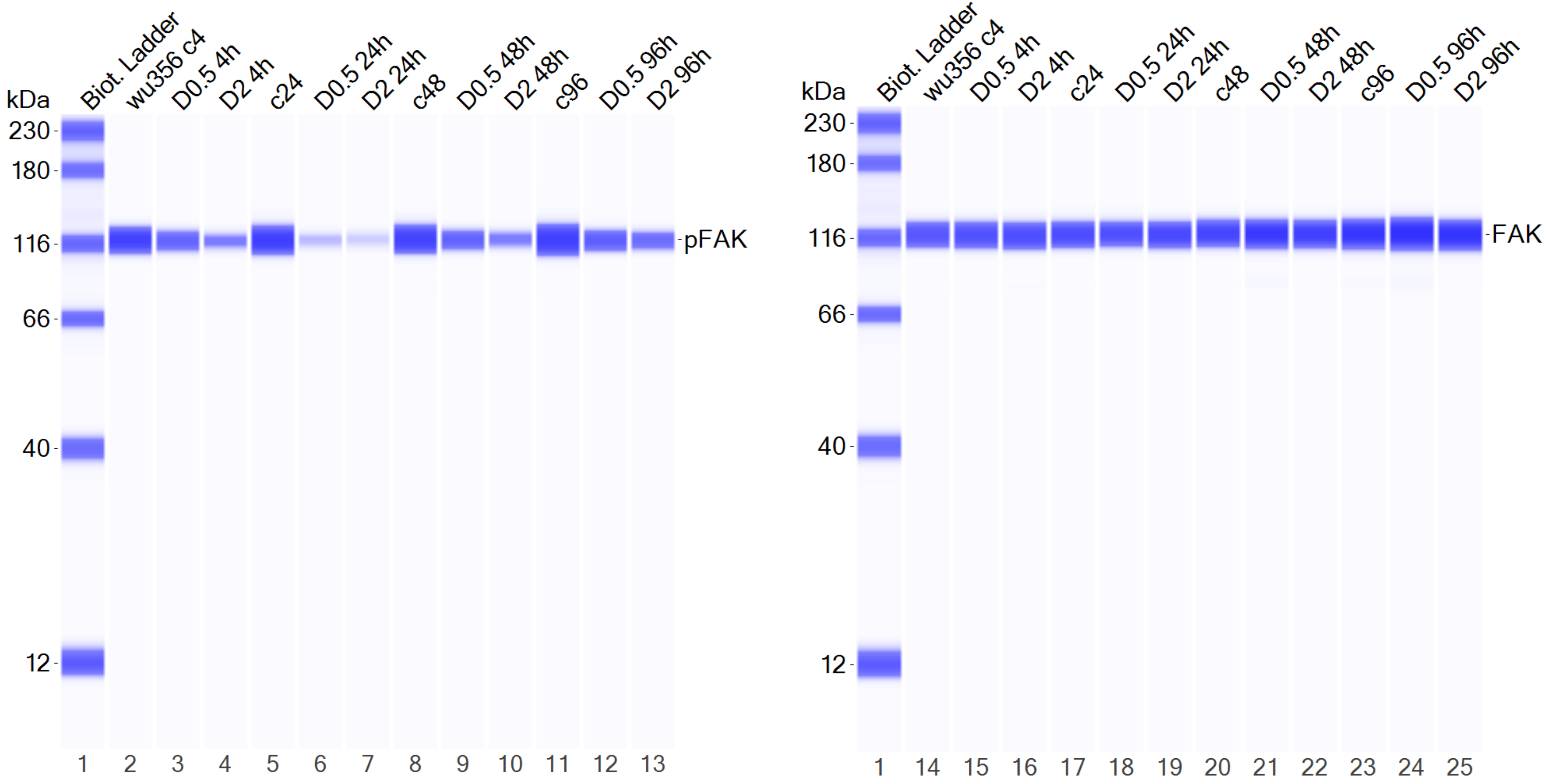

Figure 3D

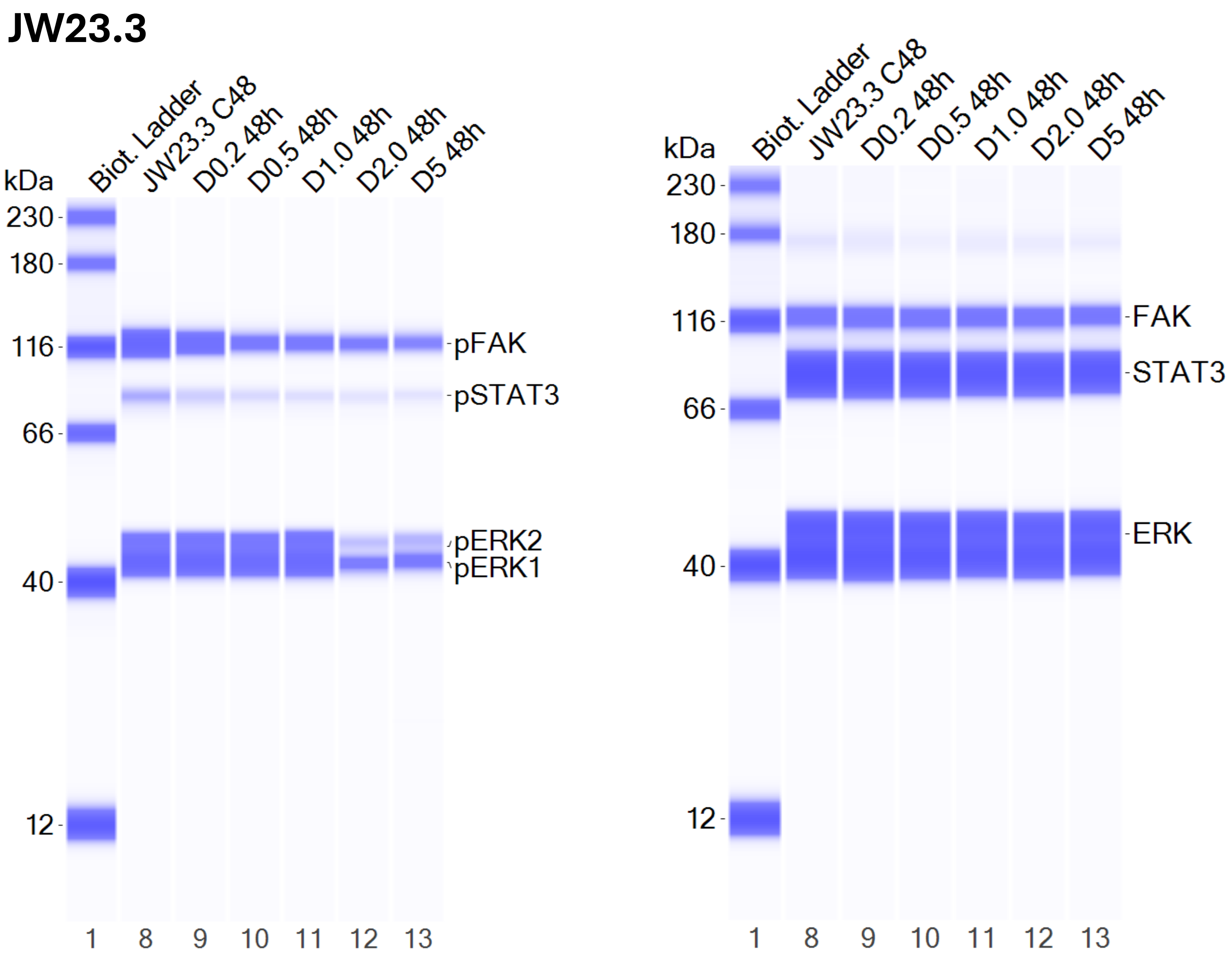
JW23.3
kDa
Biot. Ladder
JW23.3 C48
D0.2 48h
D0.5 48h
D1.0 48h
D2.0 48h
D5 48h
230
180
116
66
40
12
pFAK
pSTAT3
pERK2
pERK1
1
8
9
10
11
12
13
FAK
STAT3
ERK

Figure 3D

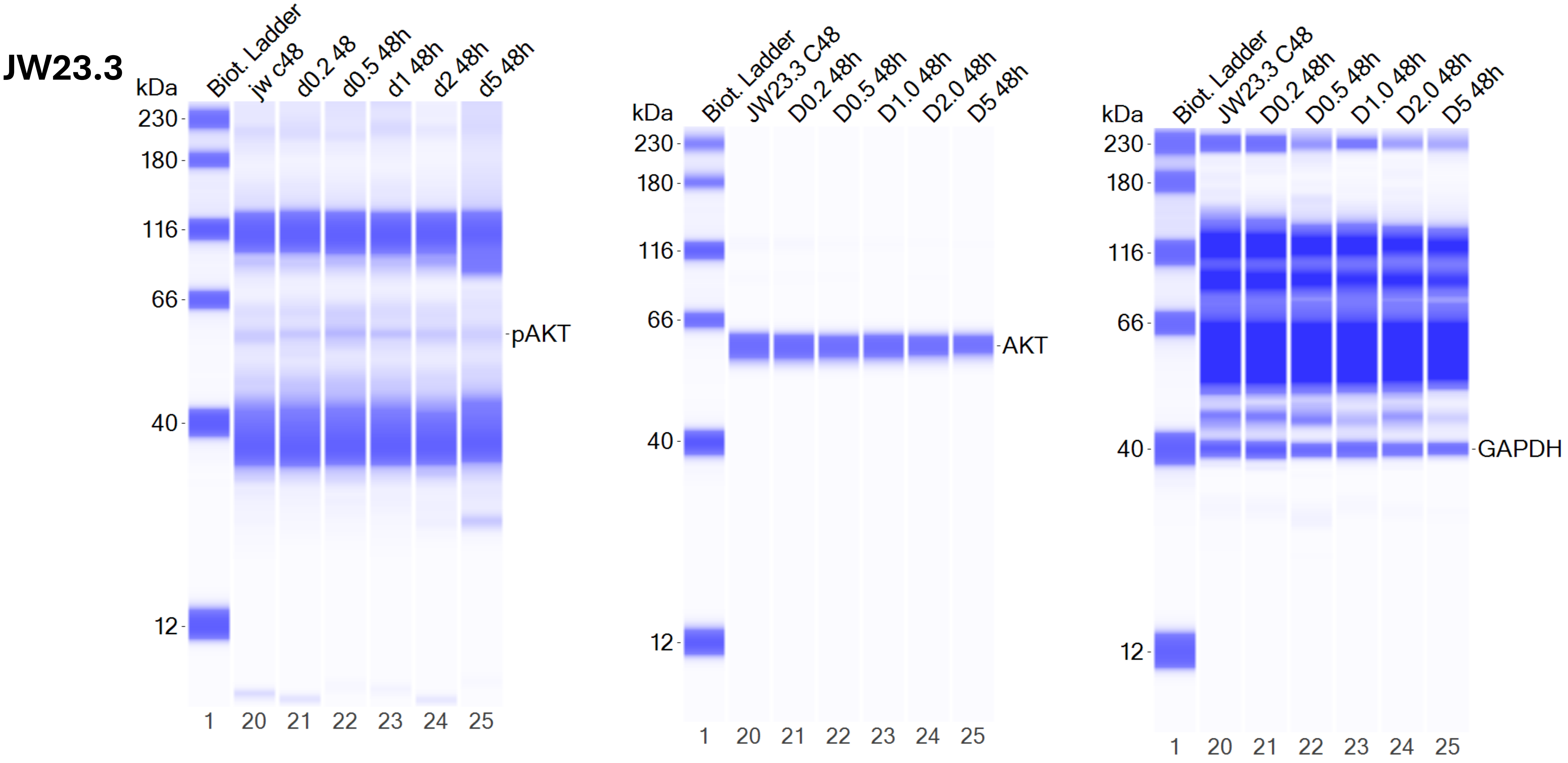

JW23.3
kDa
Biot. Ladder
jw c48
d0.2 48
d0.5 48h
d1 48h
d2 48h
d5 48h
230
180
116
66
40
12
pAKT
1
20
21
22
23
24
25
kDa
Biot. Ladder
JW23.3 C48
D0.2 48h
D0.5 48h
D1.0 48h
D2.0 48h
D5 48h
230
180
116
66
40
12
AKT
1
20
21
22
23
24
25
kDa
Biot. Ladder
JW23.3 C48
D0.2 48h
D0.5 48h
D1.0 48h
D2.0 48h
D5 48h
230
180
116
66
40
12
GAPDH
1
20
21
22
23
24
25

Figure 3D

JW23.3

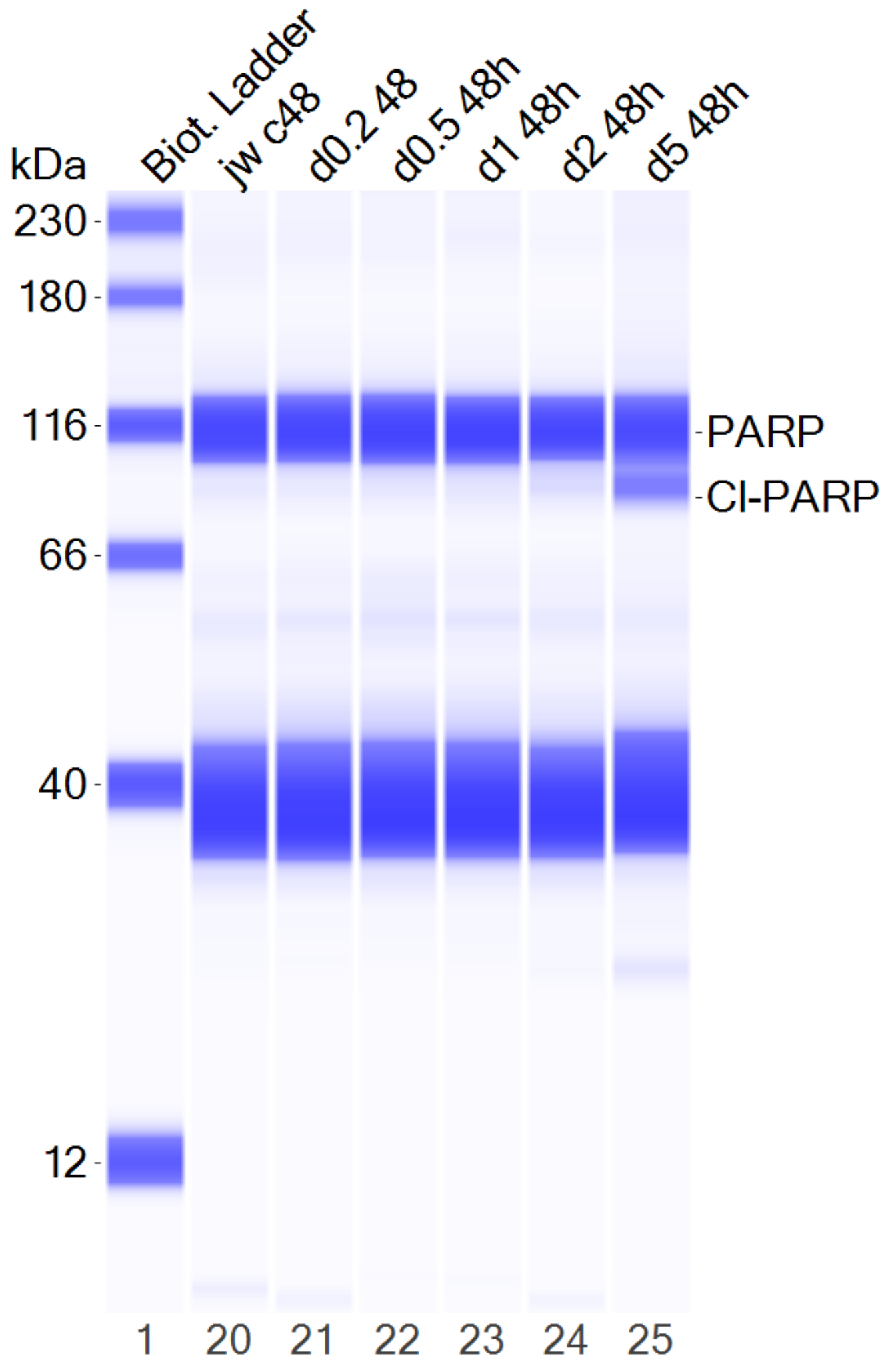

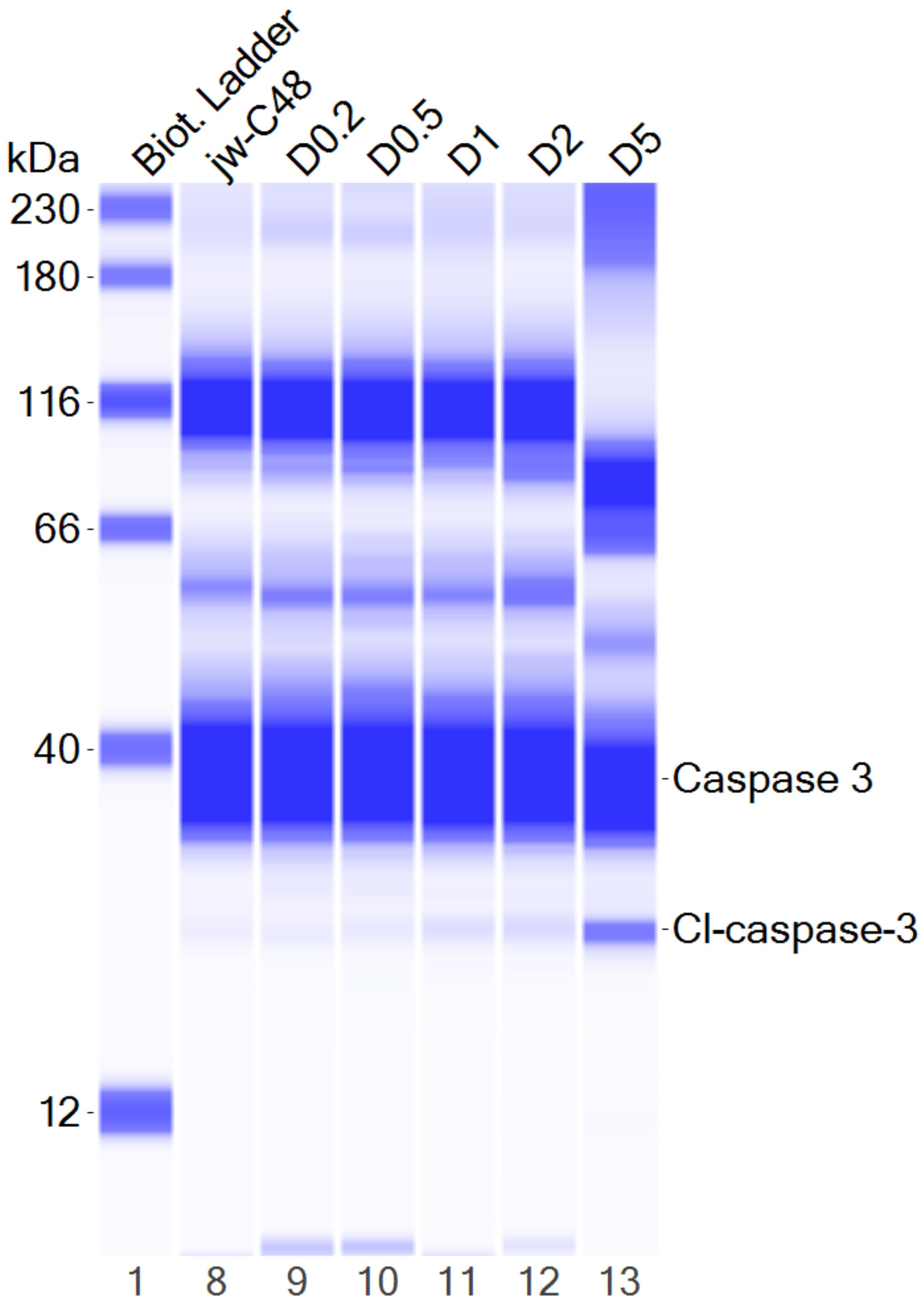

Figure 3D

JH-2-002

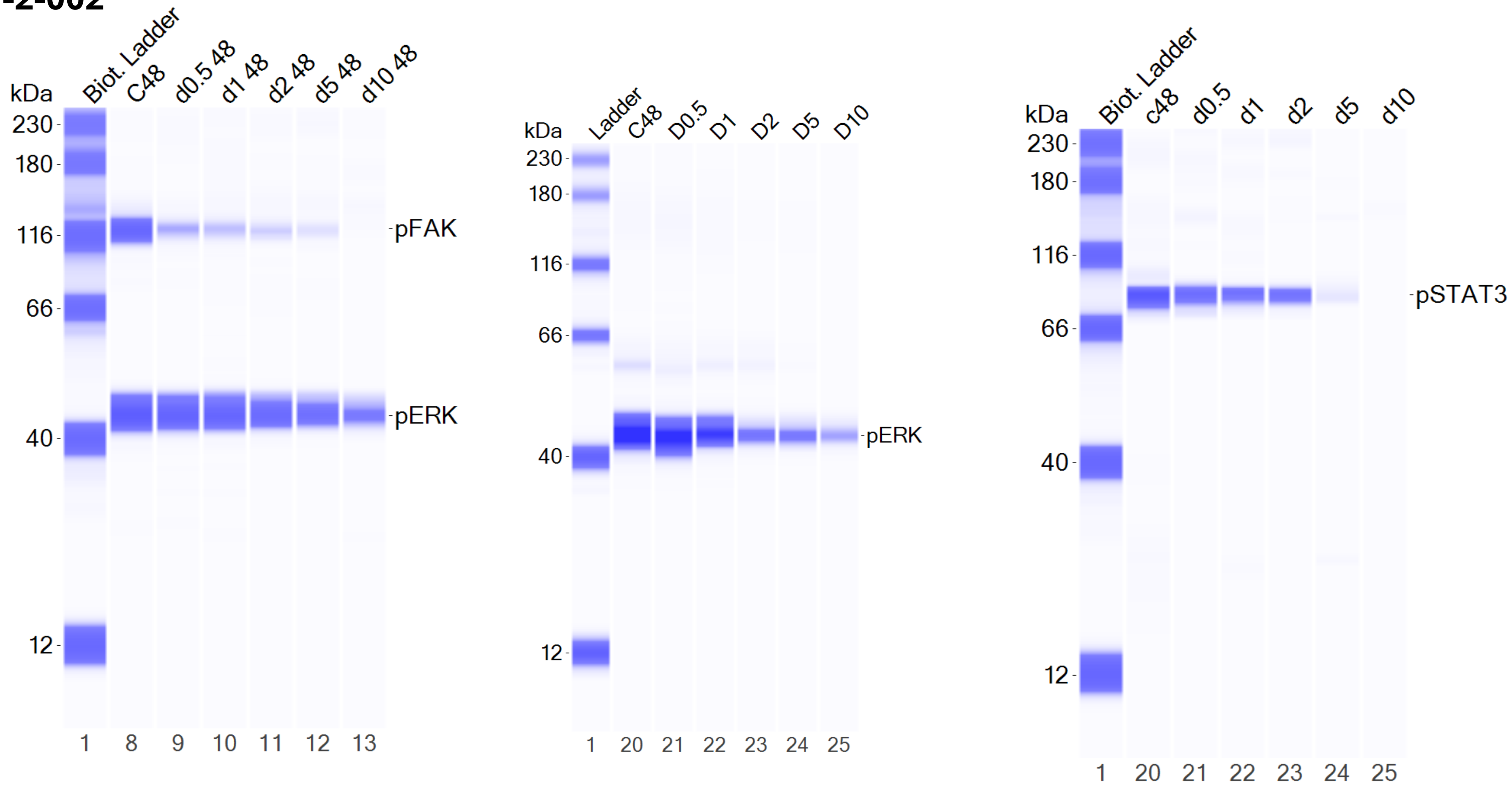

Figure 3D

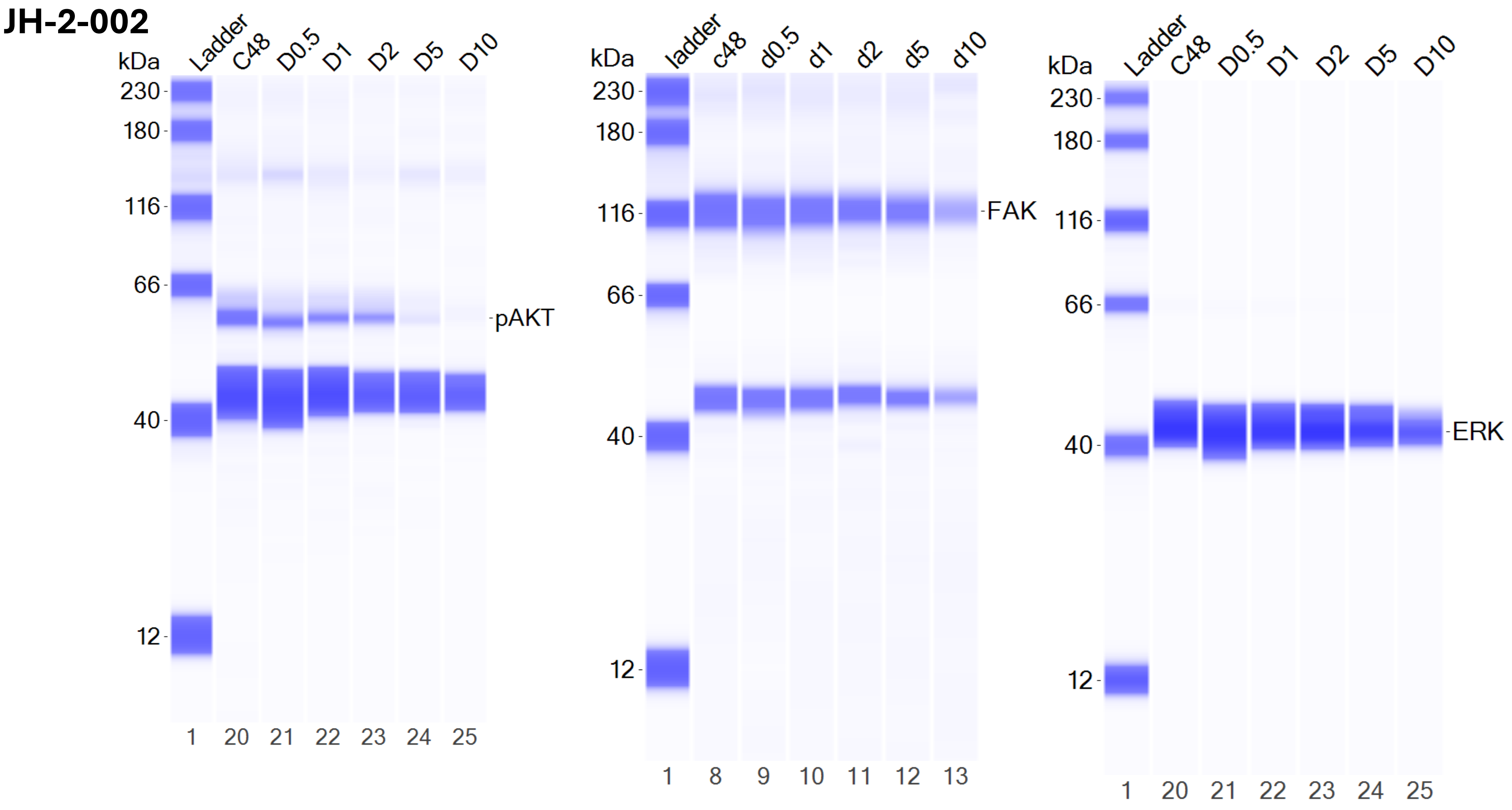

JH-2-002
kDa
Ladder
C48
D0.5
D1
D2
D5
D10
230
180
116
66
40
12
pAKT
1 20 21 22 23 24 25
kDa
ladder
c48
d0.5
d1
d2
d5
d10
230
180
116
66
40
12
FAK
1 8 9 10 11 12 13
kDa
Ladder
C48
D0.5
D1
D2
D5
D10
230
180
116
66
40
12
ERK
1 20 21 22 23 24 25

Figure 3D

JH-2-002

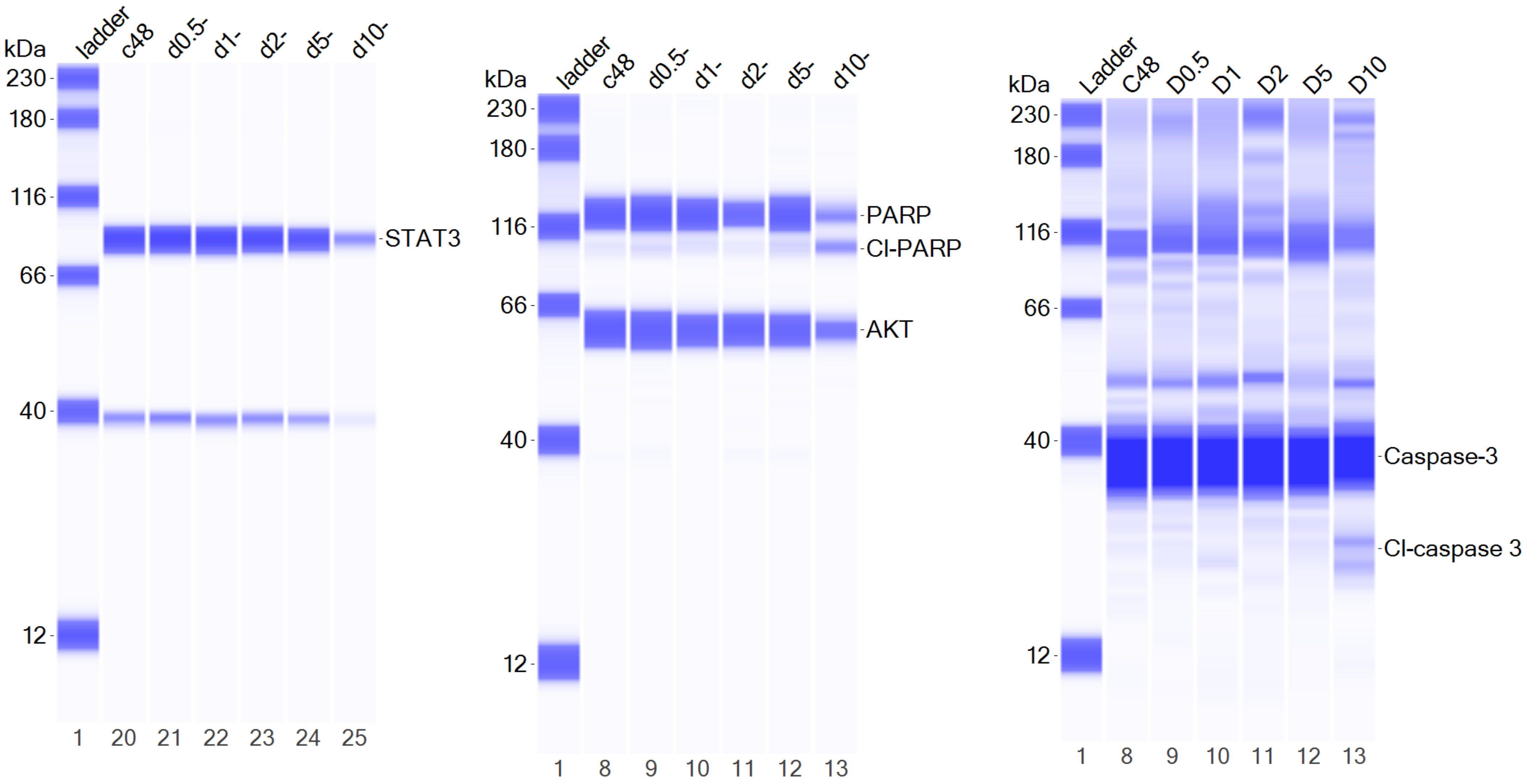

Figure 3D

JH-2-002

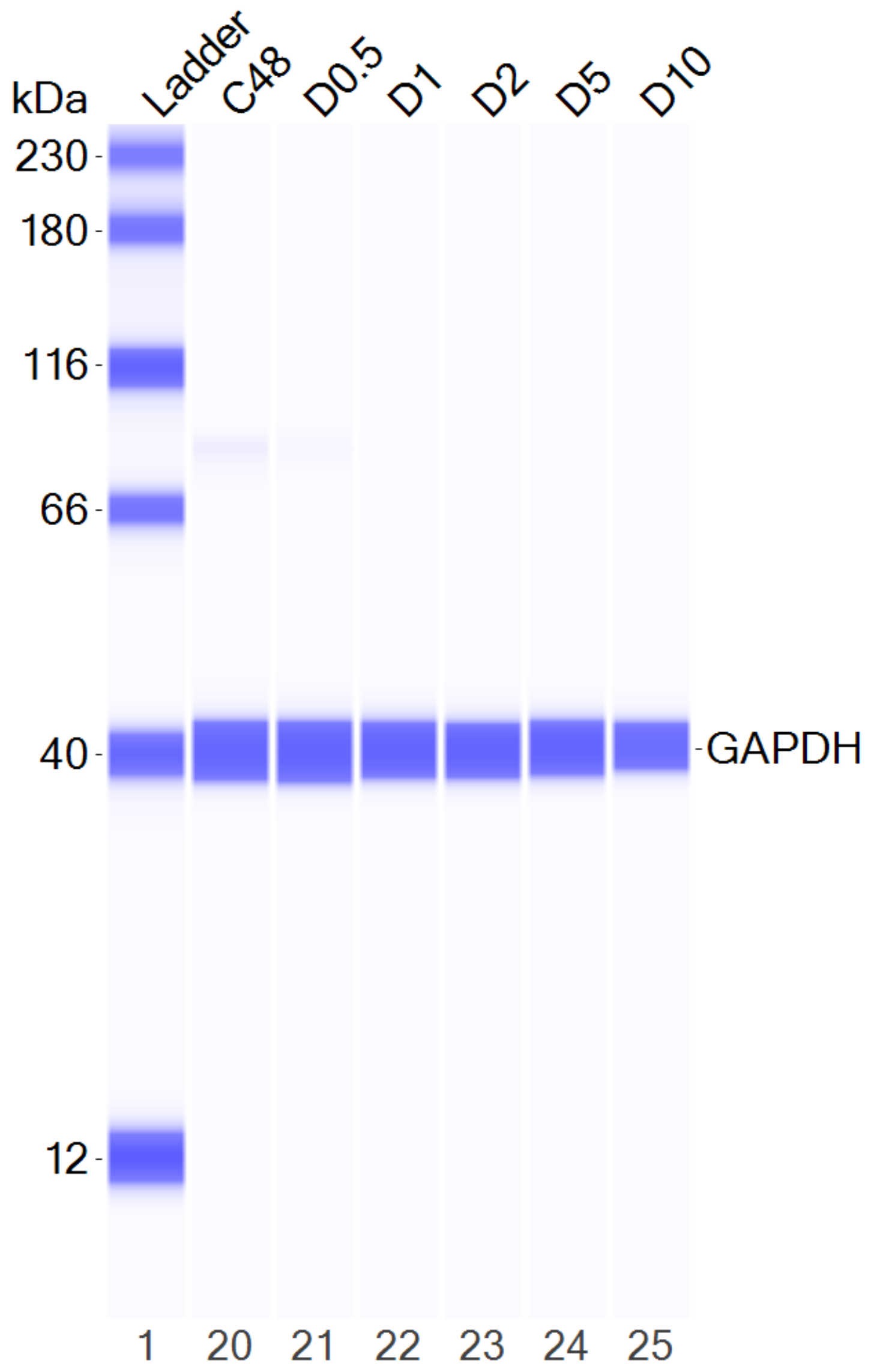

Figure 3D

WU356

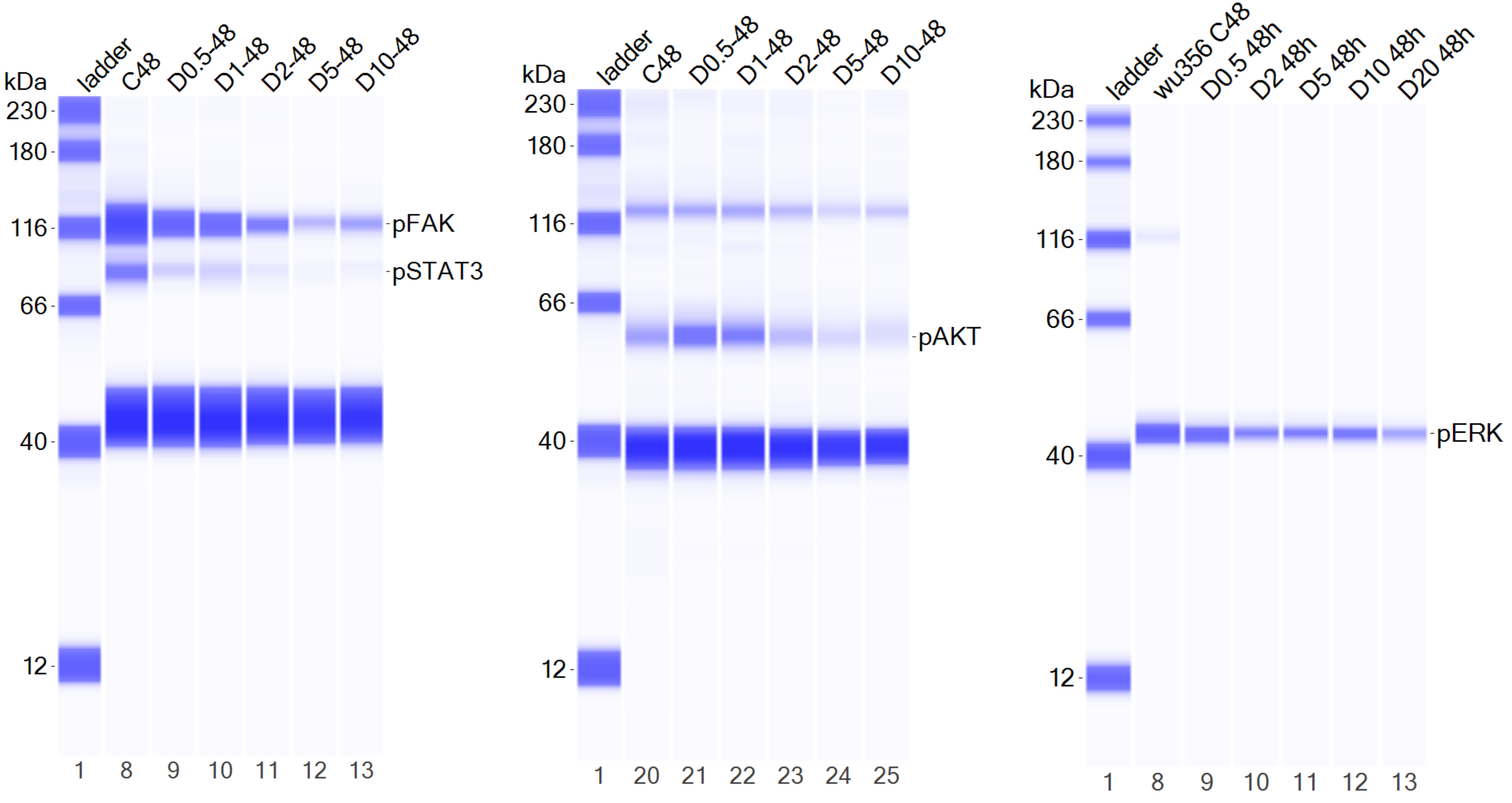

Figure 3D

WU356

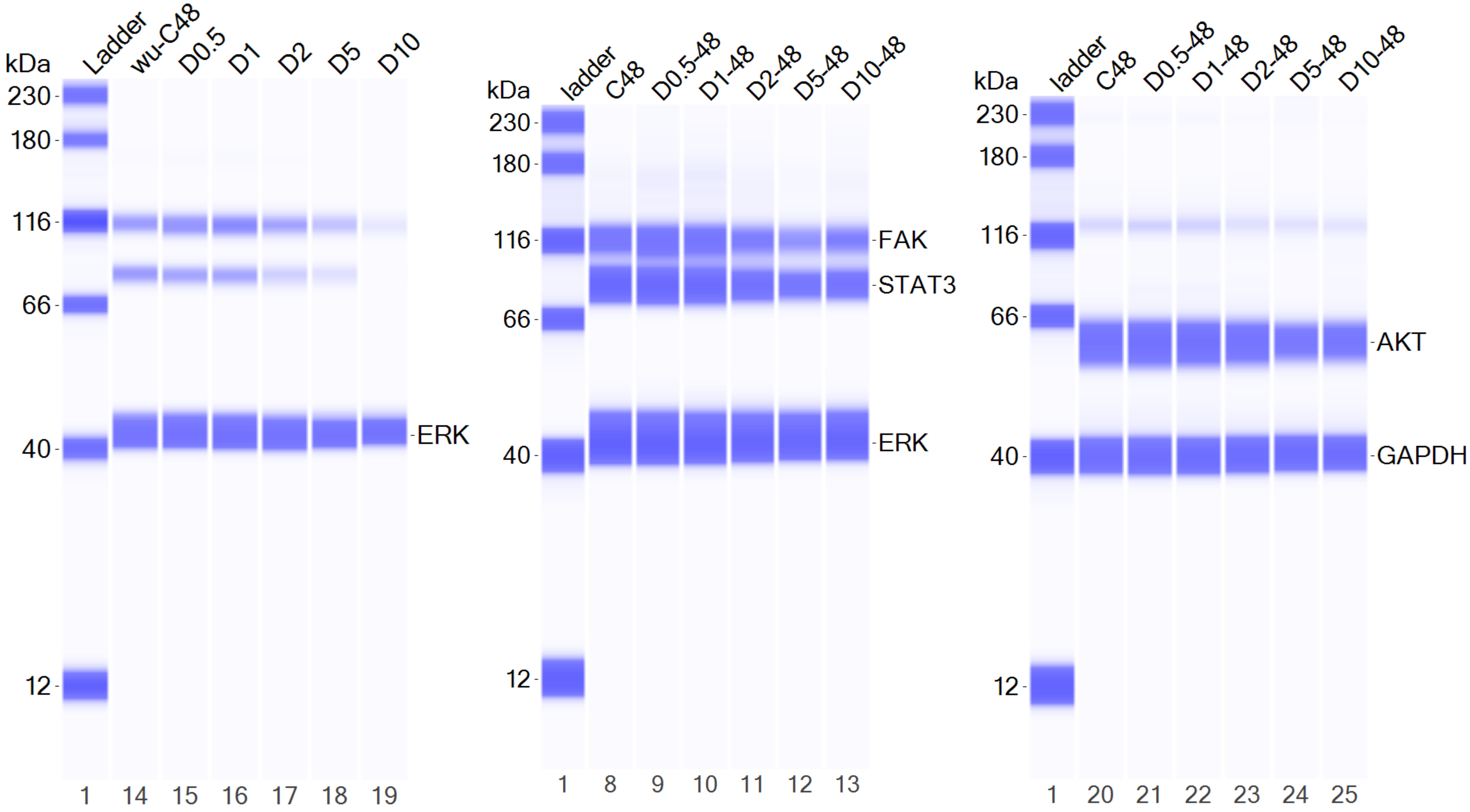

Figure 3D

WU356

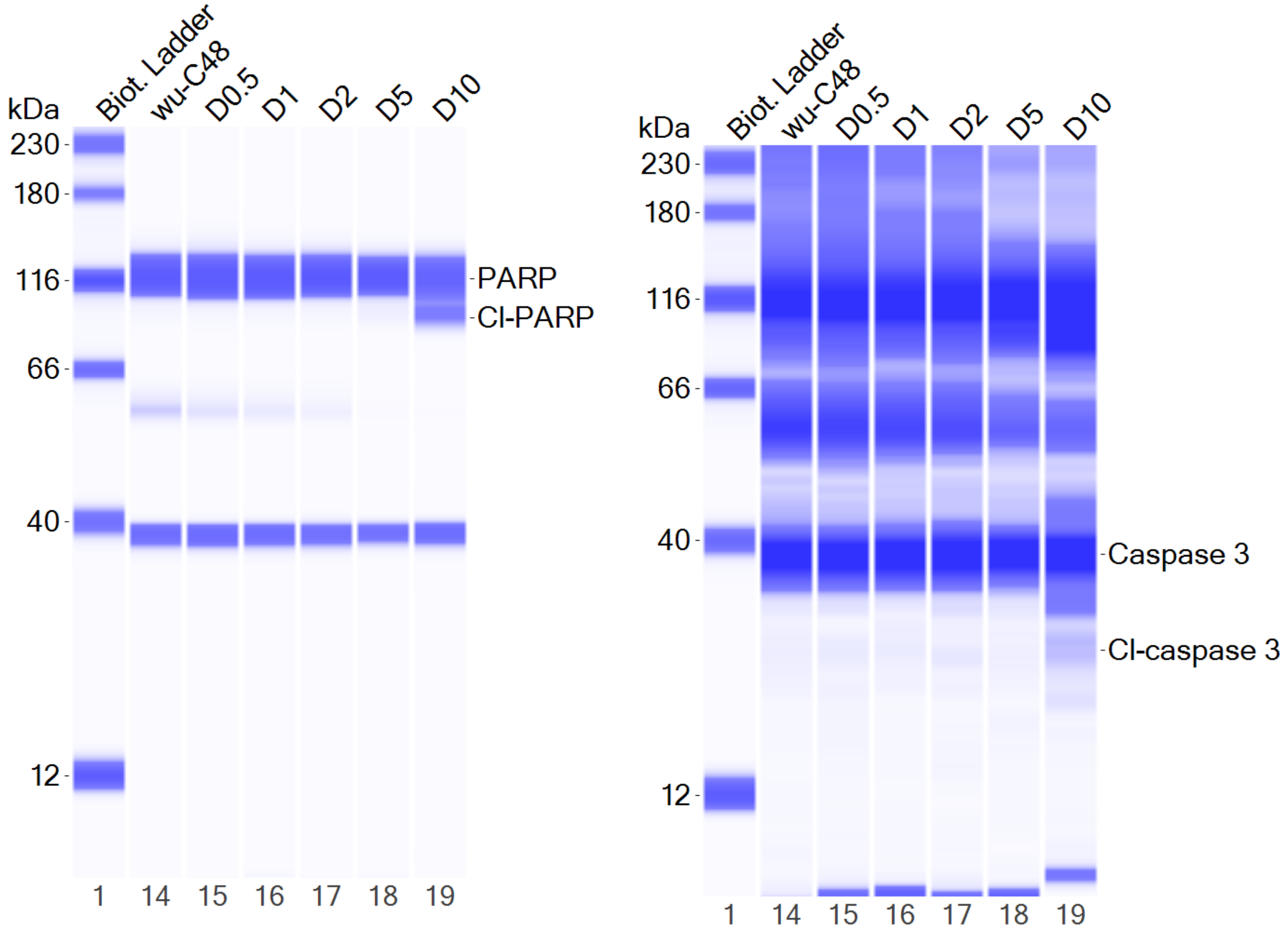

Figure 5C

JW23.3

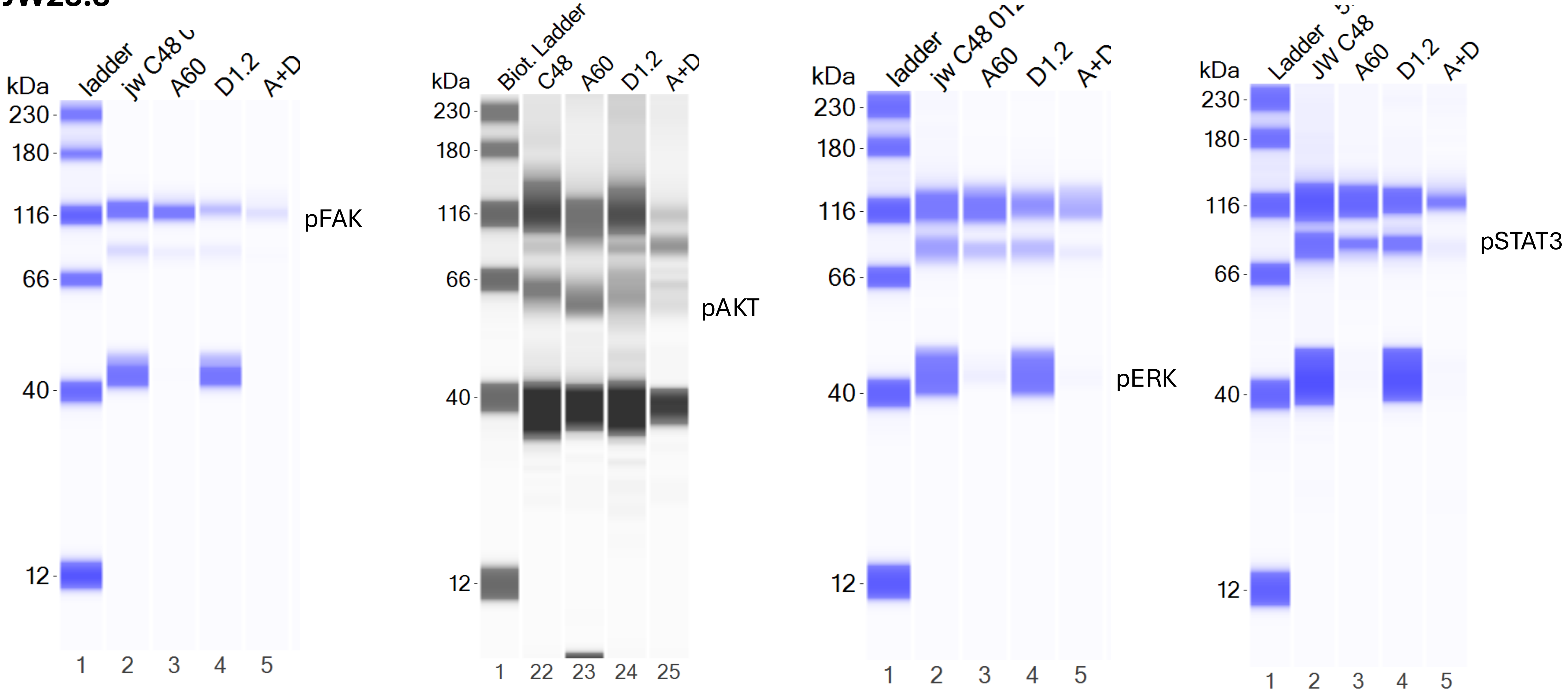

Figure 5C

JW23.3

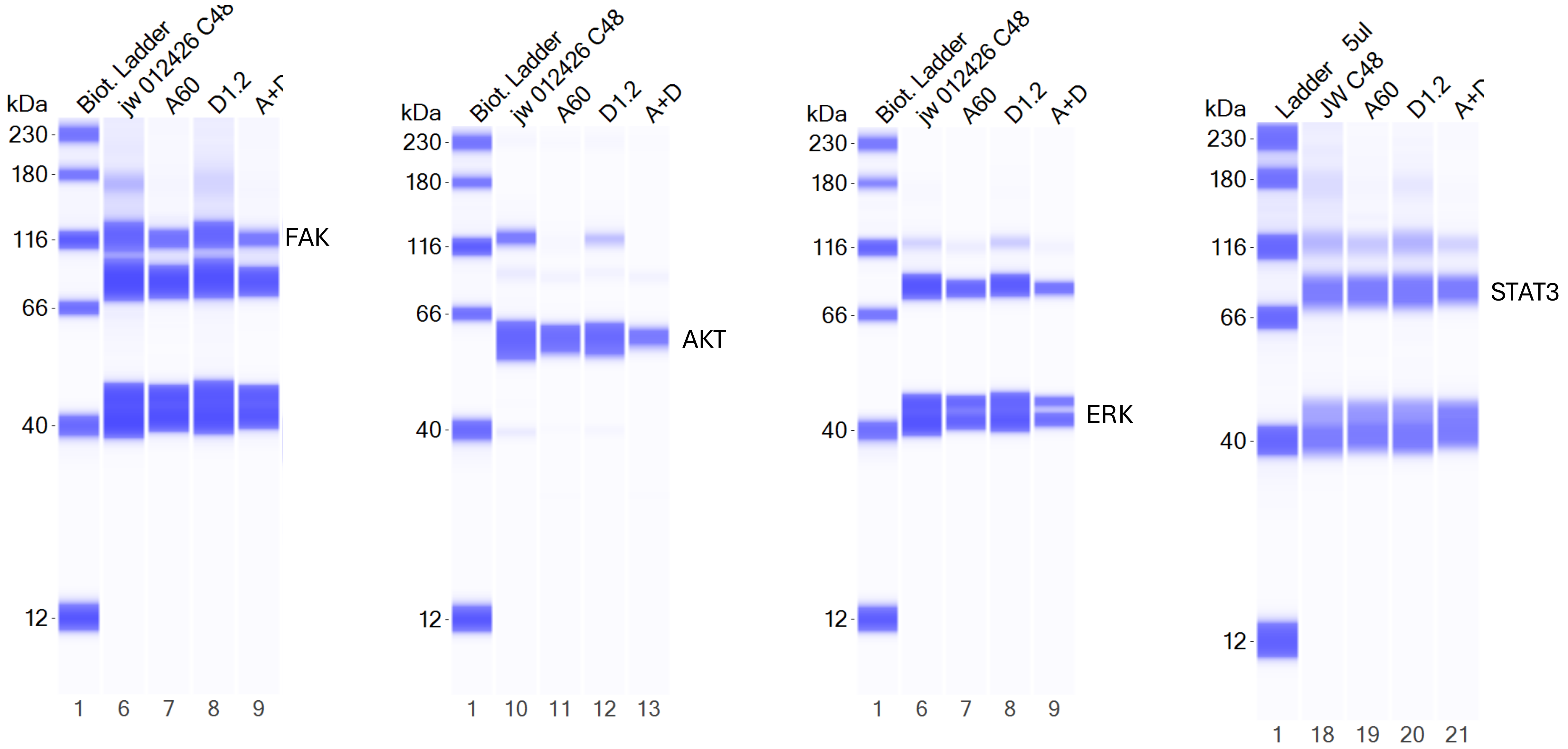

Figure 5C

JW23.3

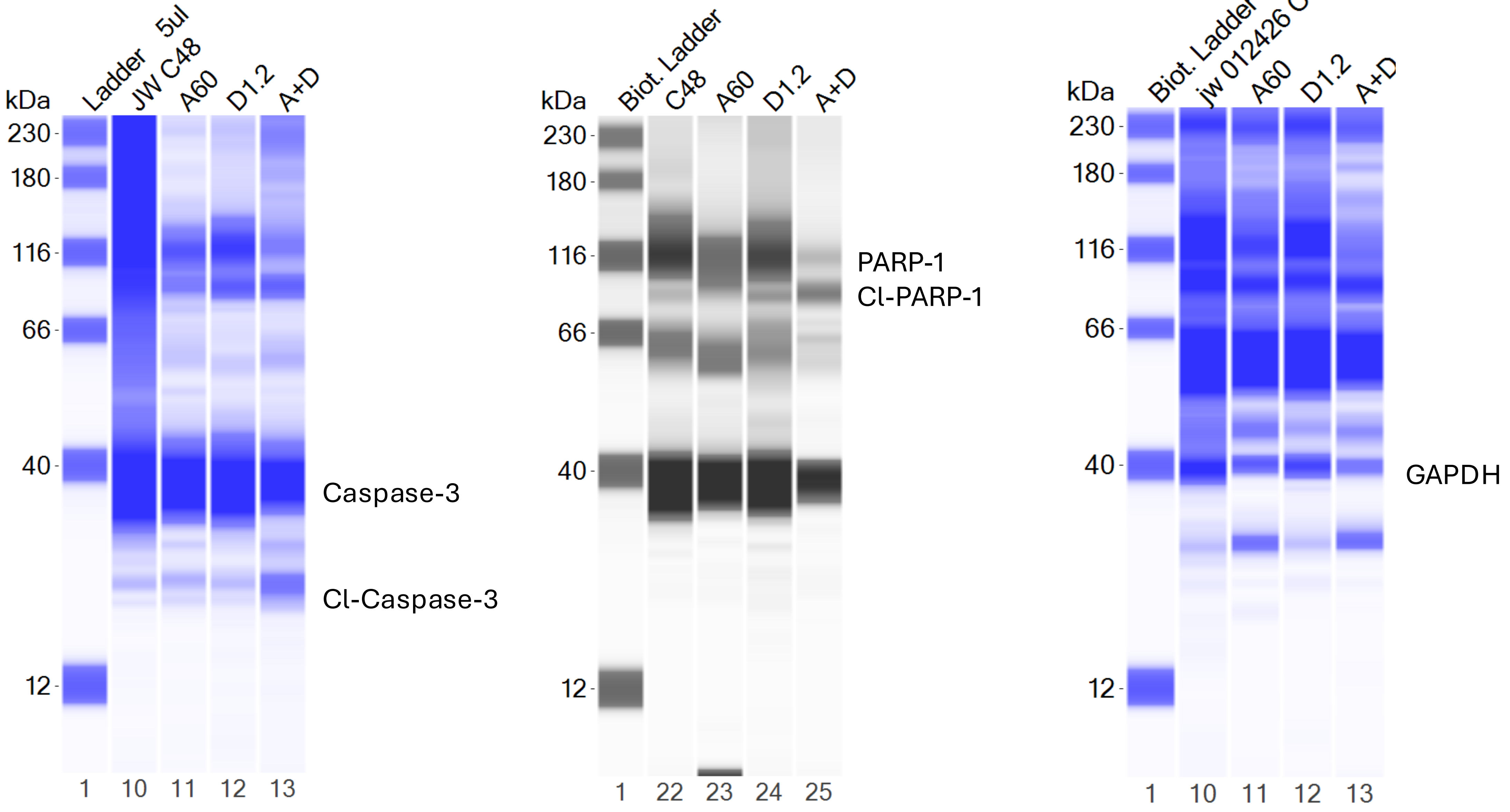

Figure 5C

JH-2-002

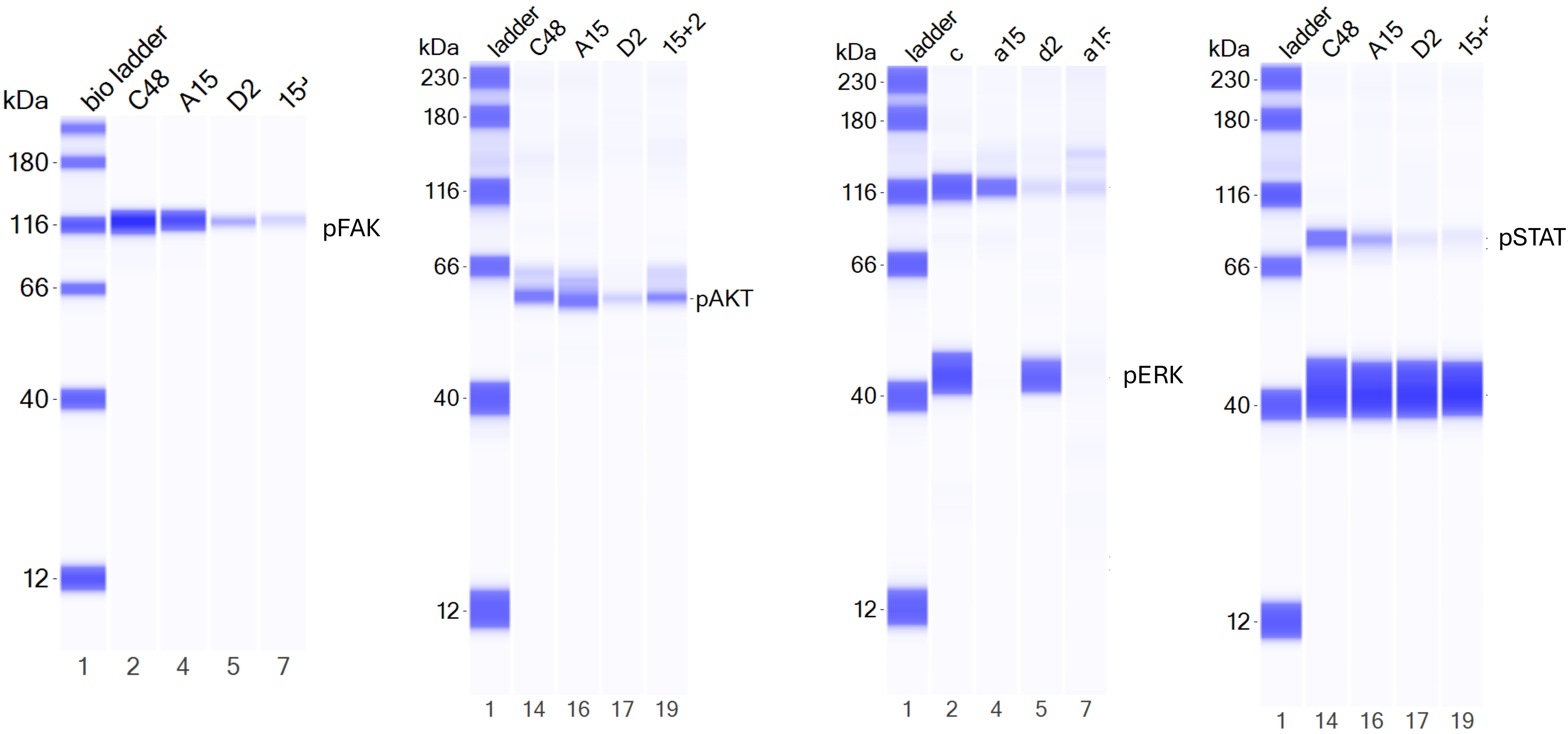

Figure 5C

JH-2-002

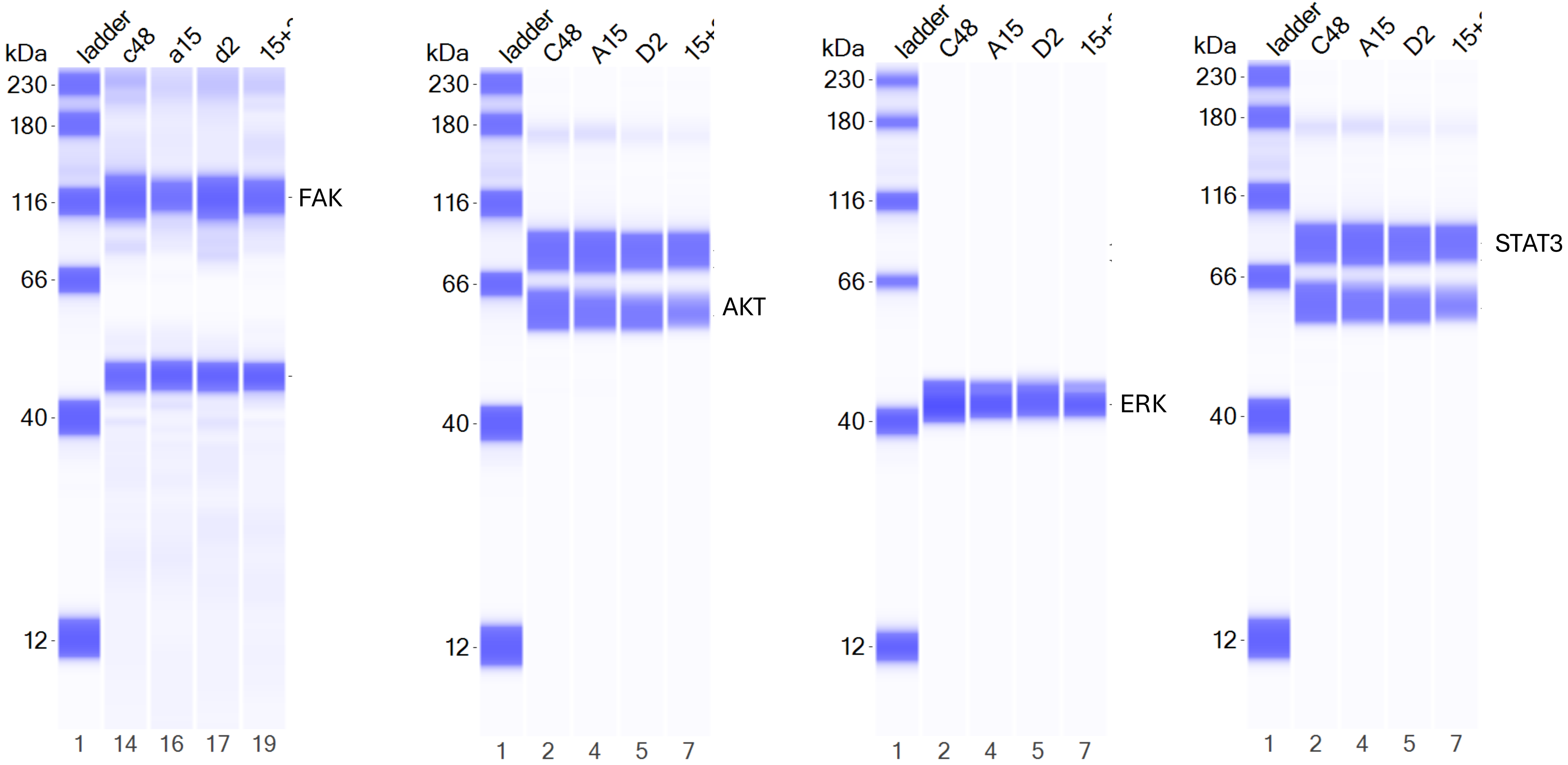

Figure 5C

JH-2-002

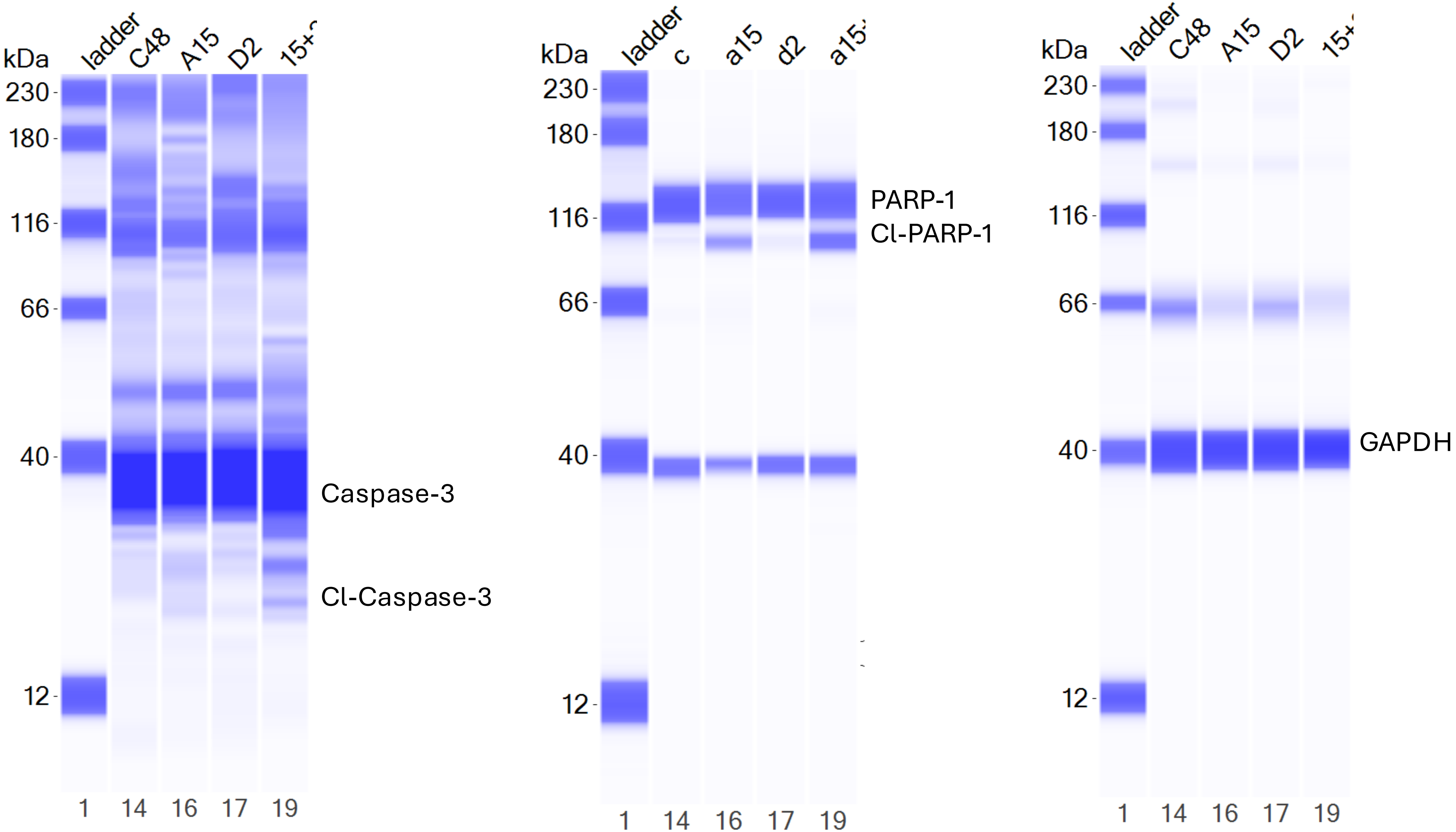

Figure 5C

WU356

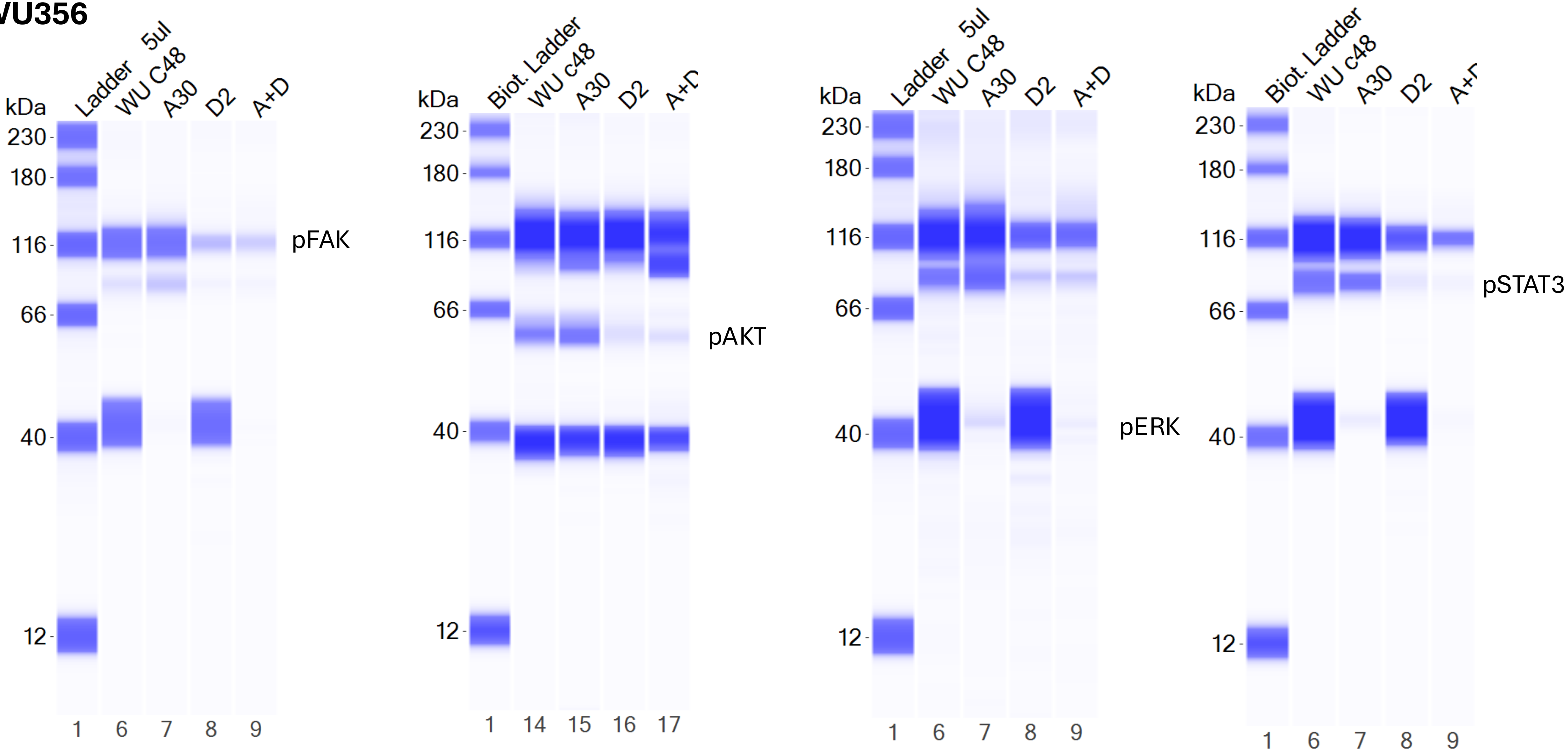

Figure 5C

WU356

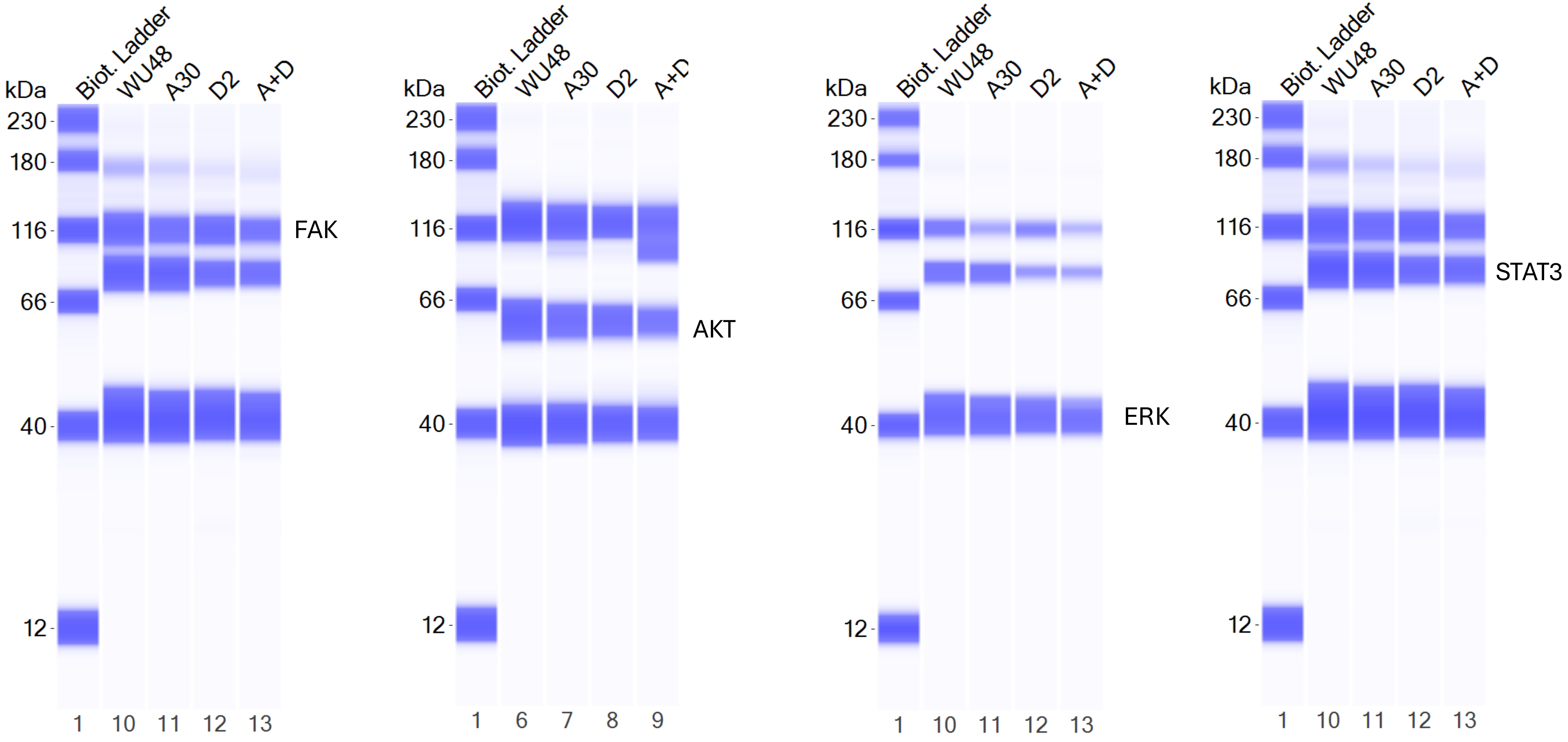

Figure 5C

WU356

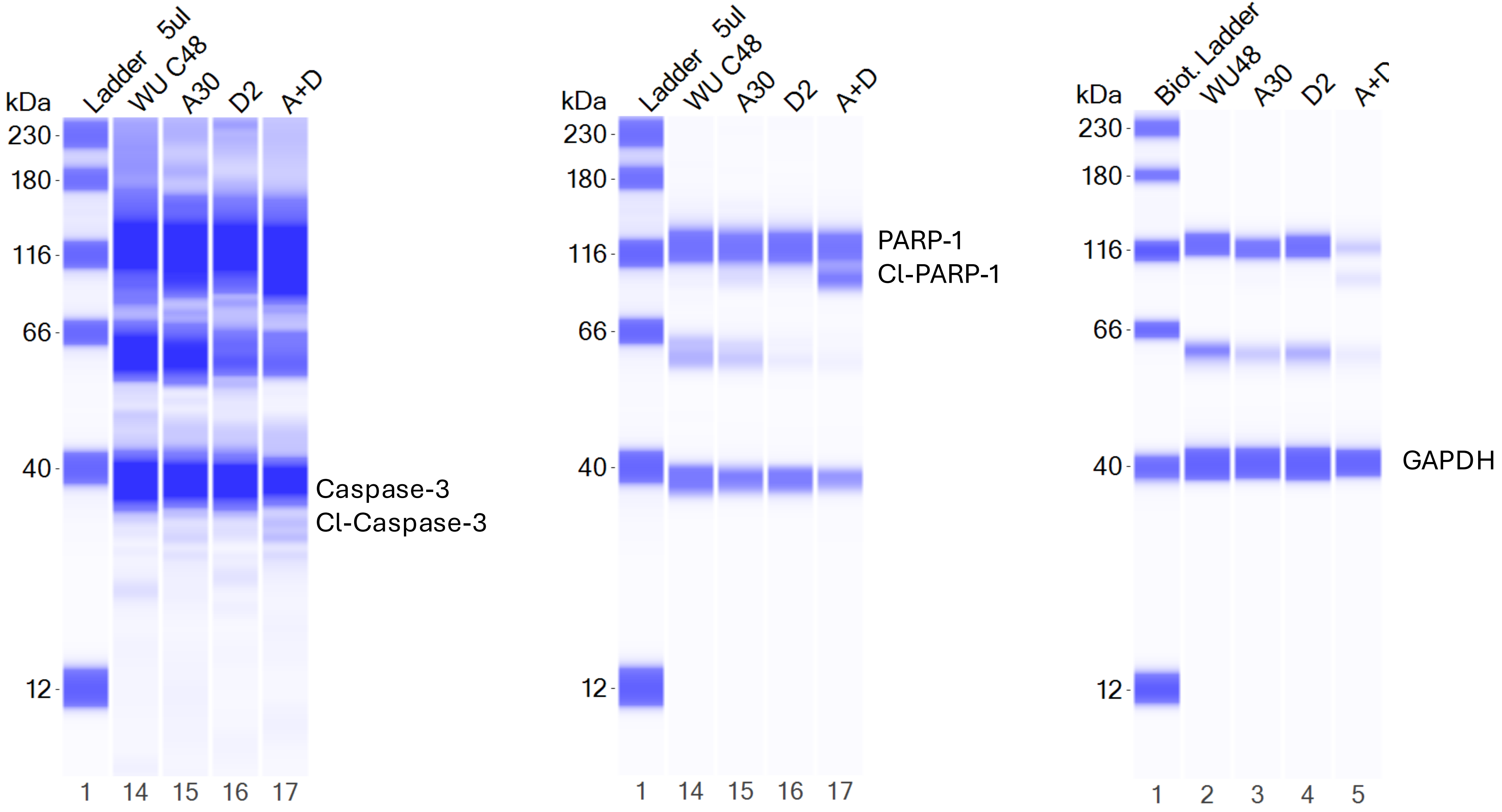

Supplementary Figure 7A

JH-2-002

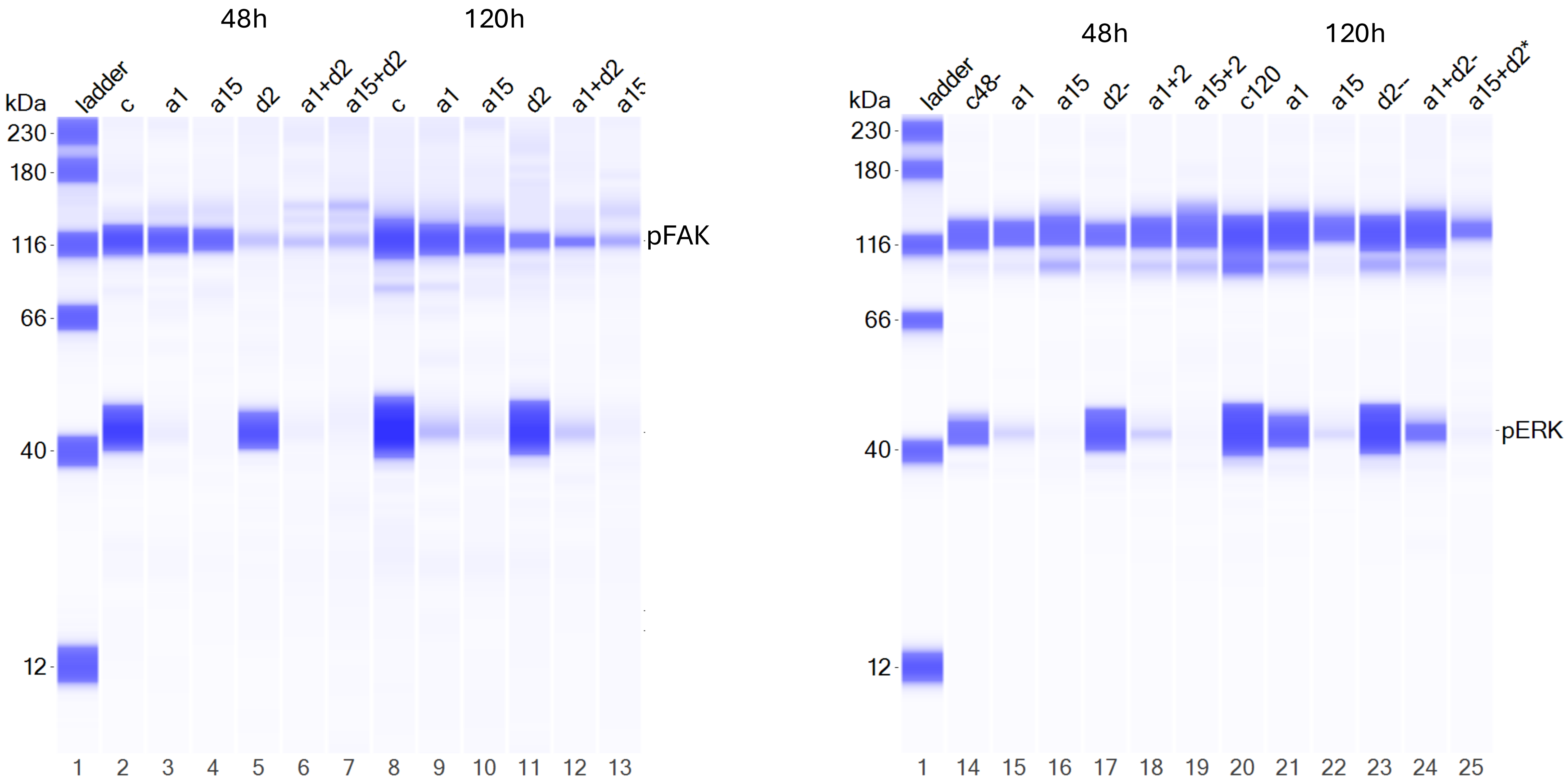

Supplementary Figure 7A

JH-2-002

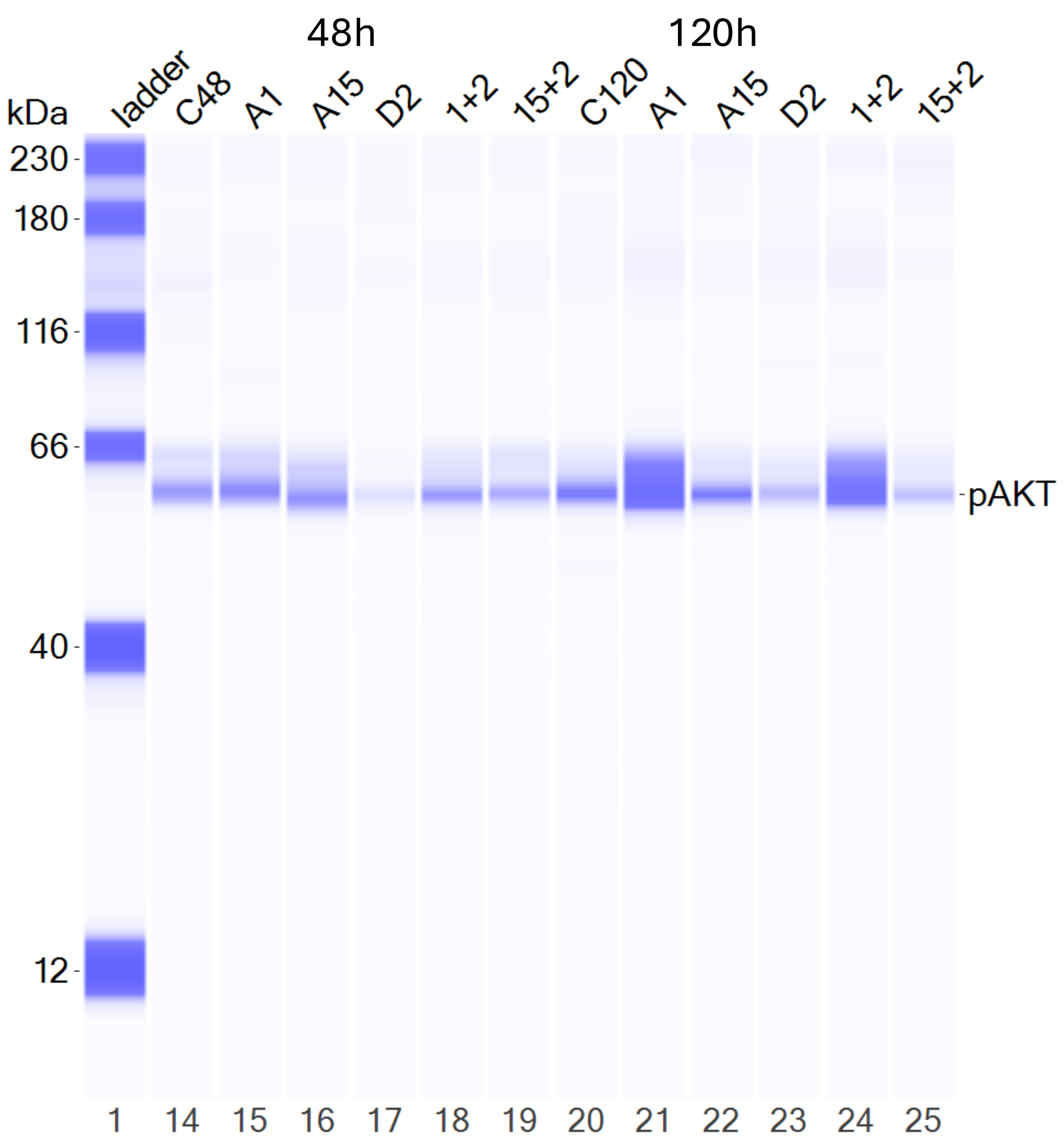

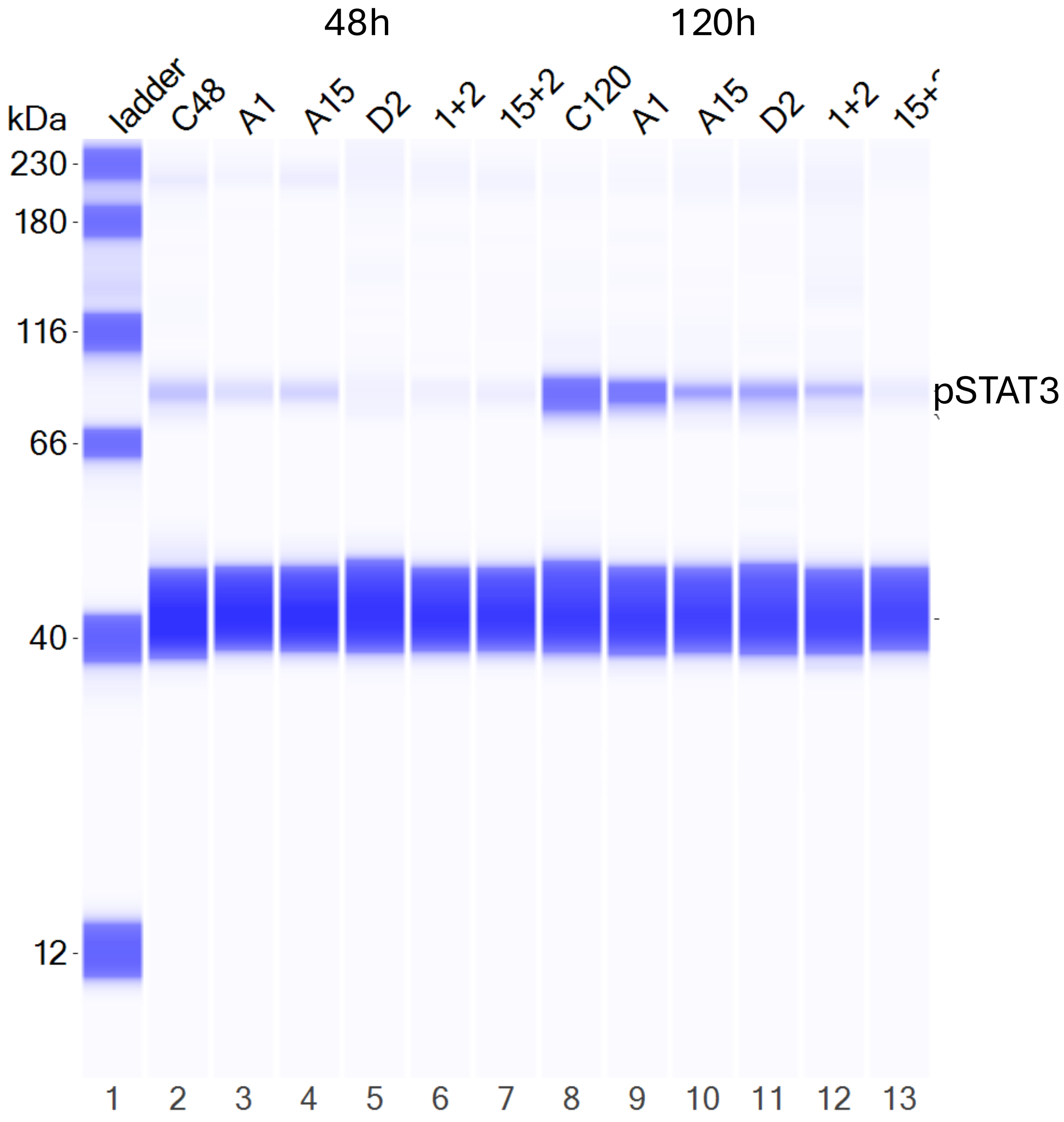

Supplementary Figure 7A

JH-2-002

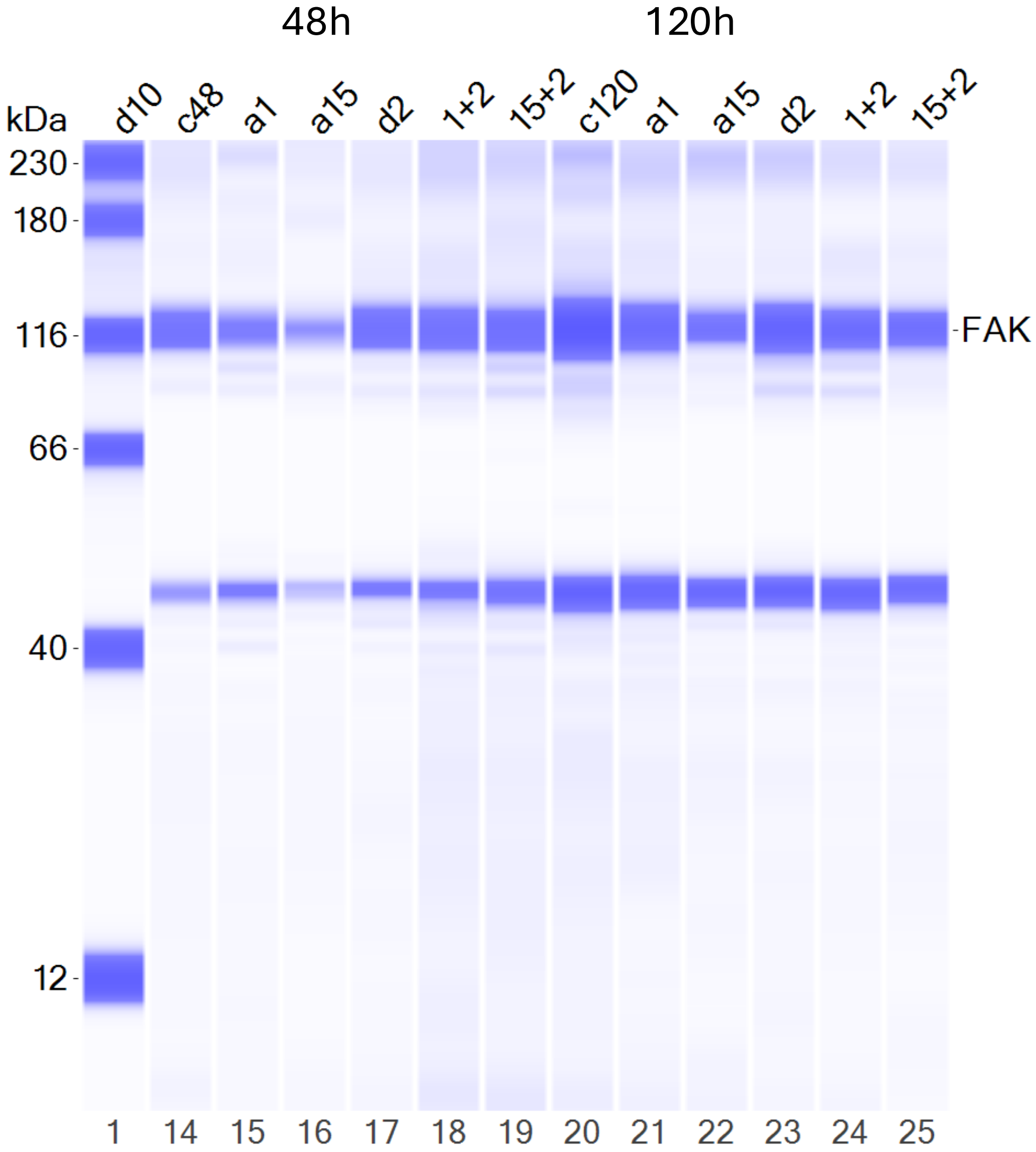

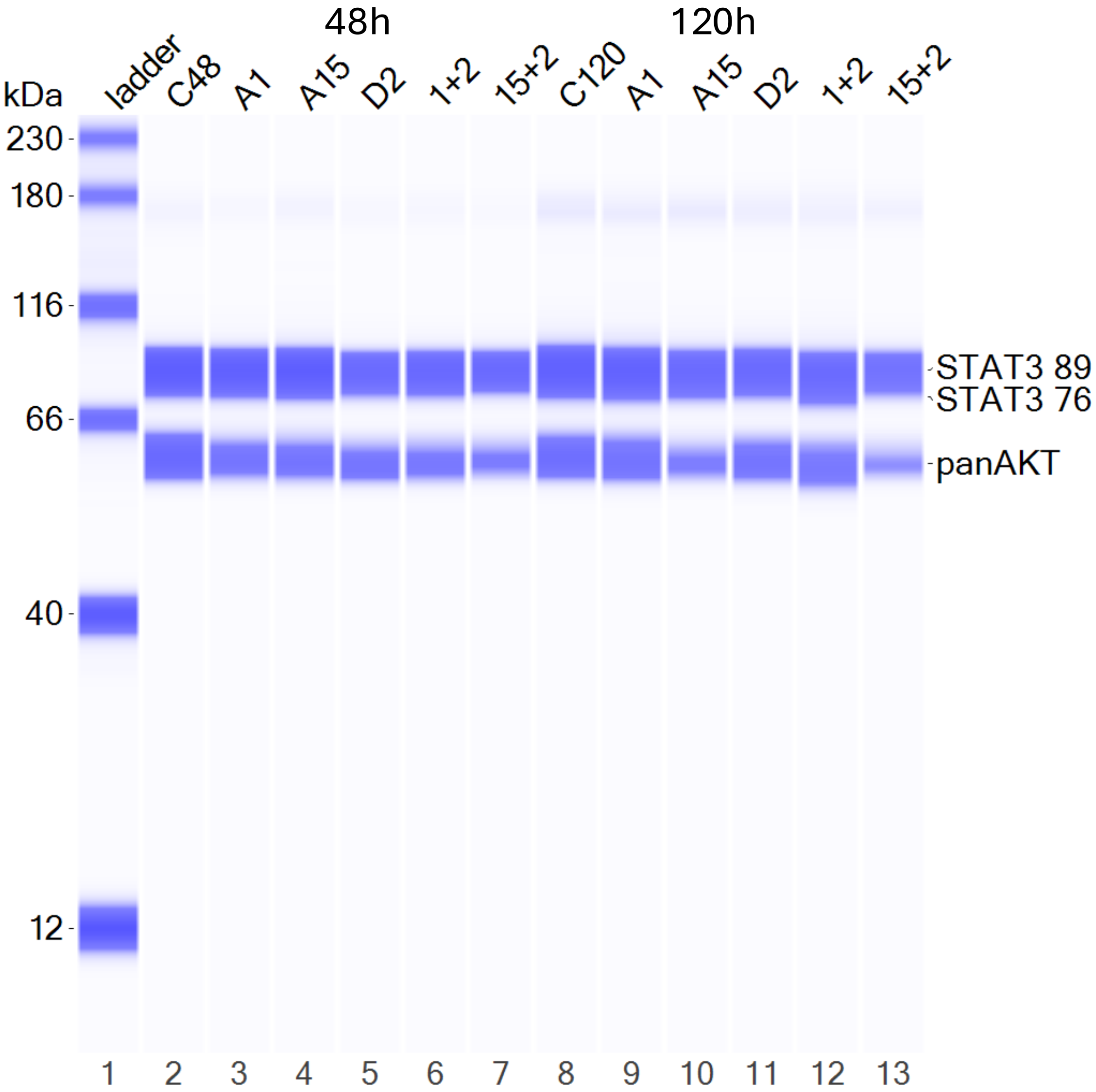

Supplementary Figure 7A

JH-2-002

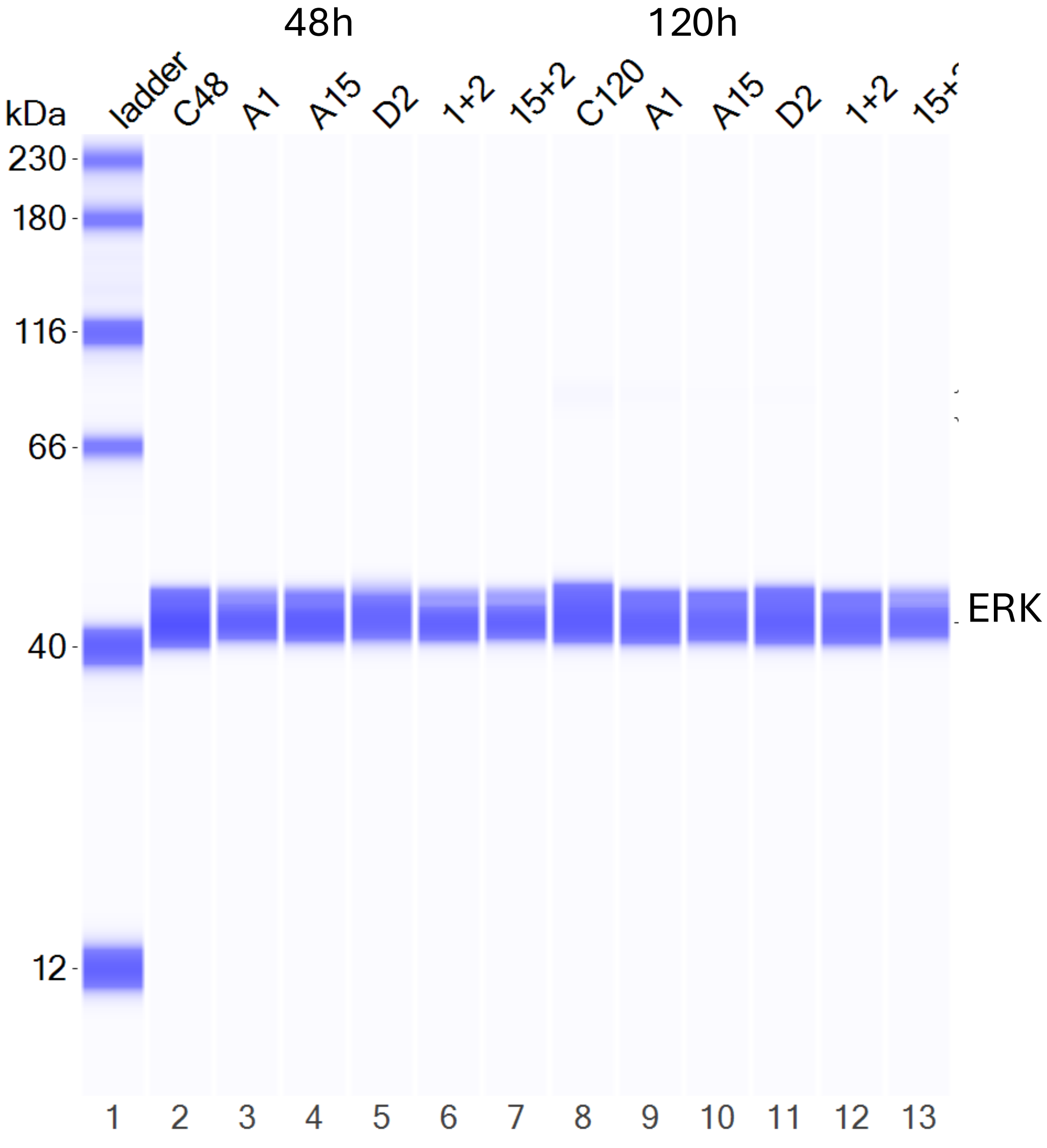

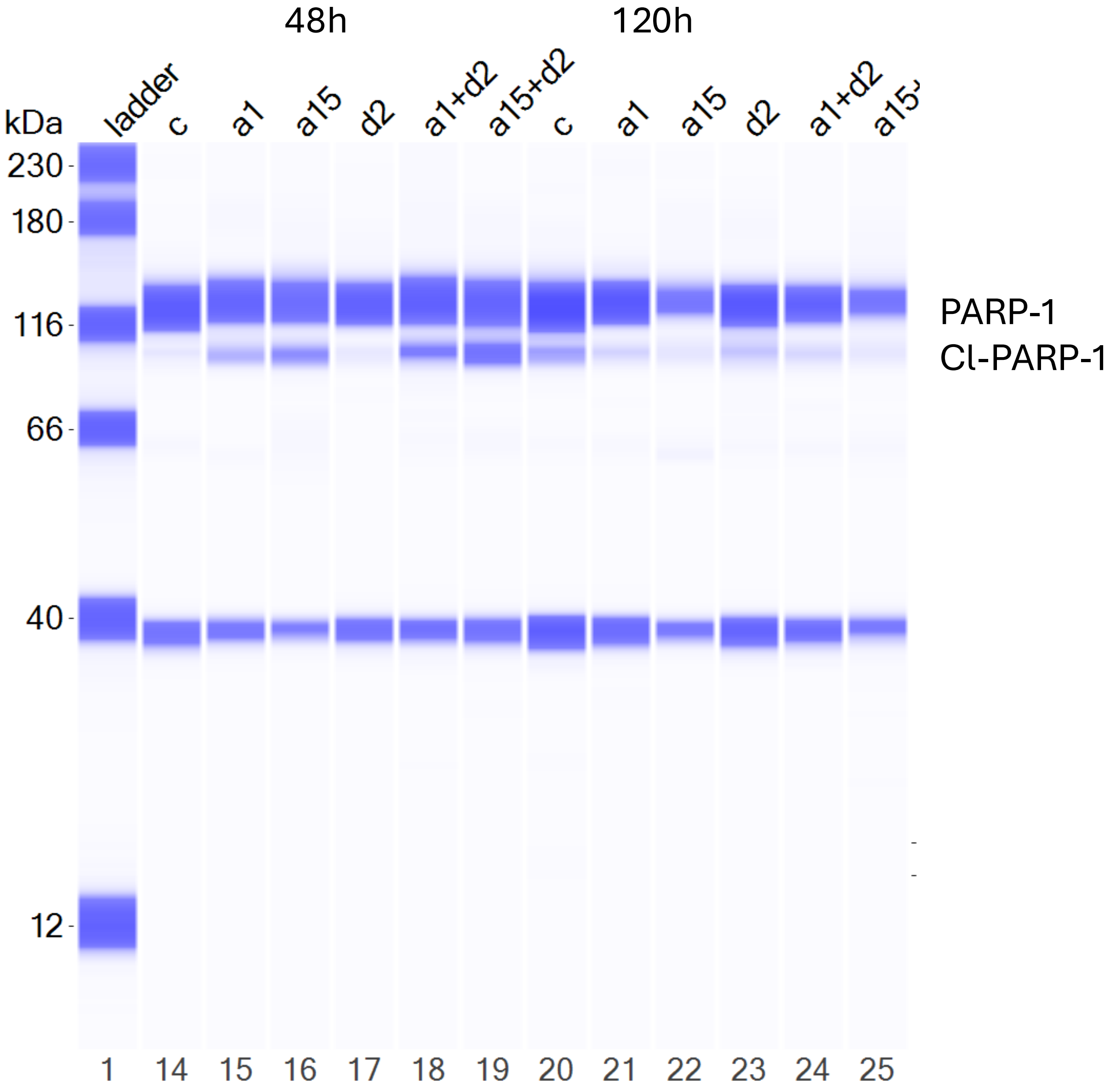

Supplementary Figure 7A

JH-2-002

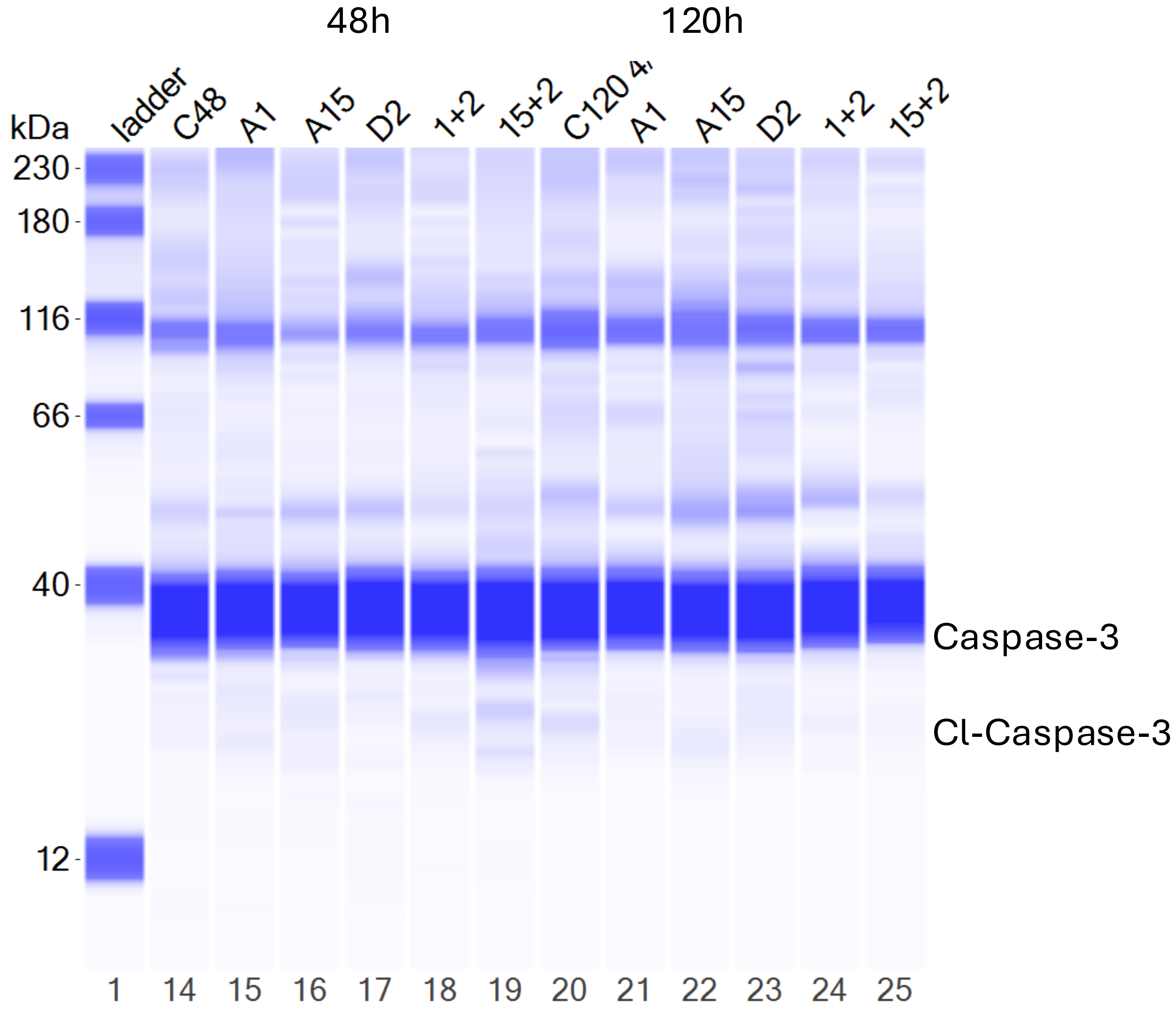

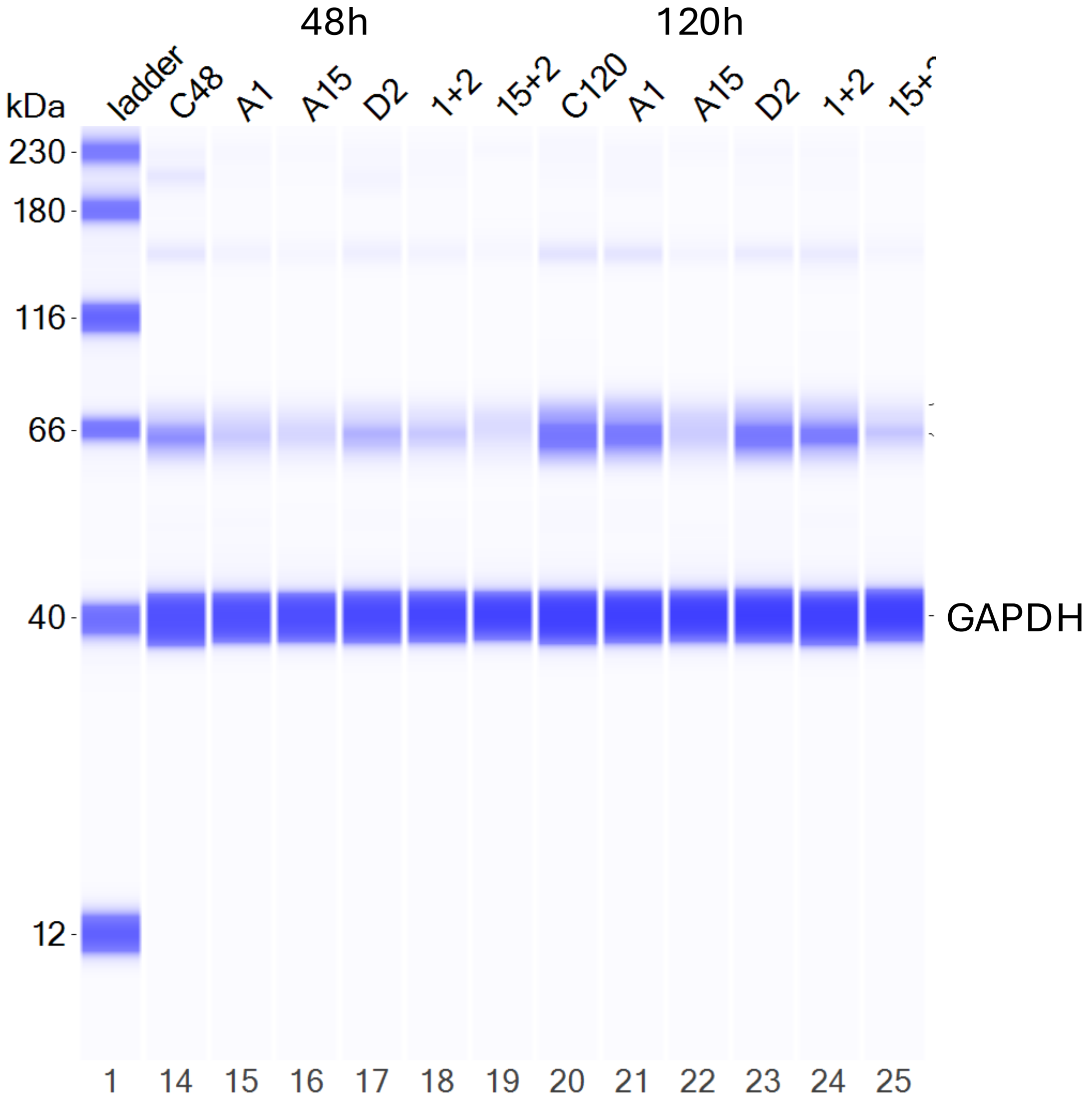

Supplementary Figure 7C

JW23.3

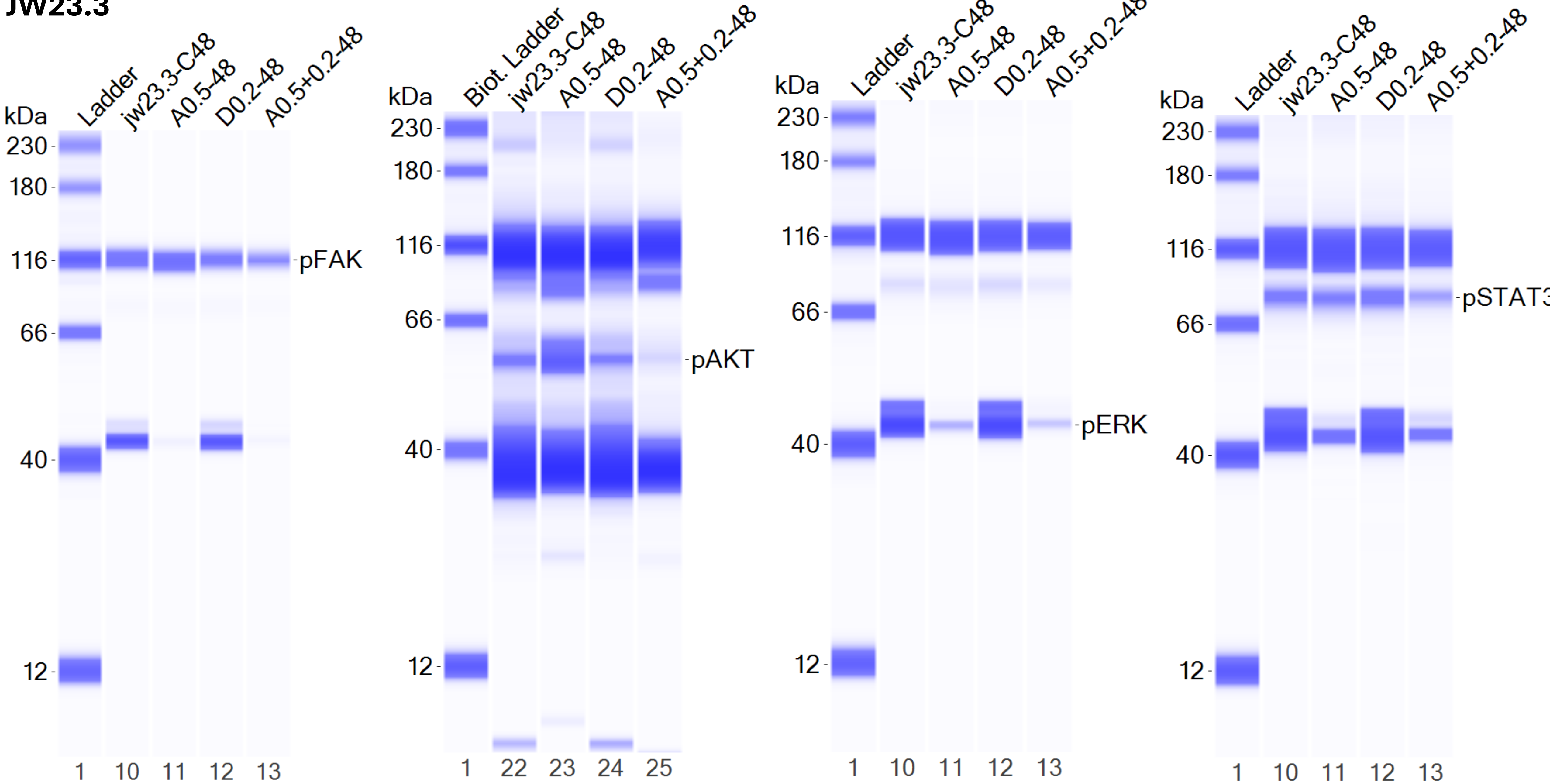

Supplementary Figure 7C

JW23.3

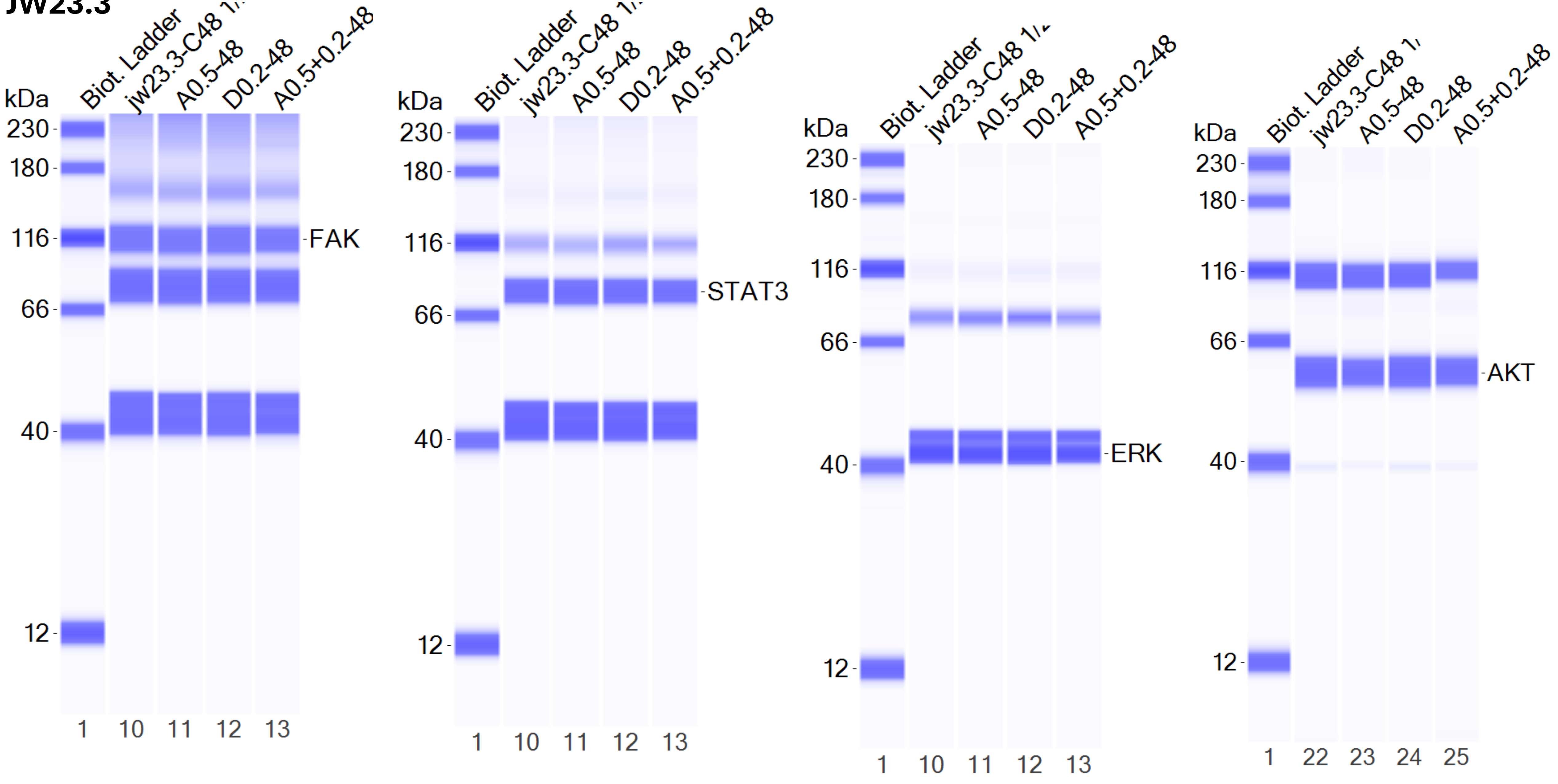

Supplementary Figure 7C

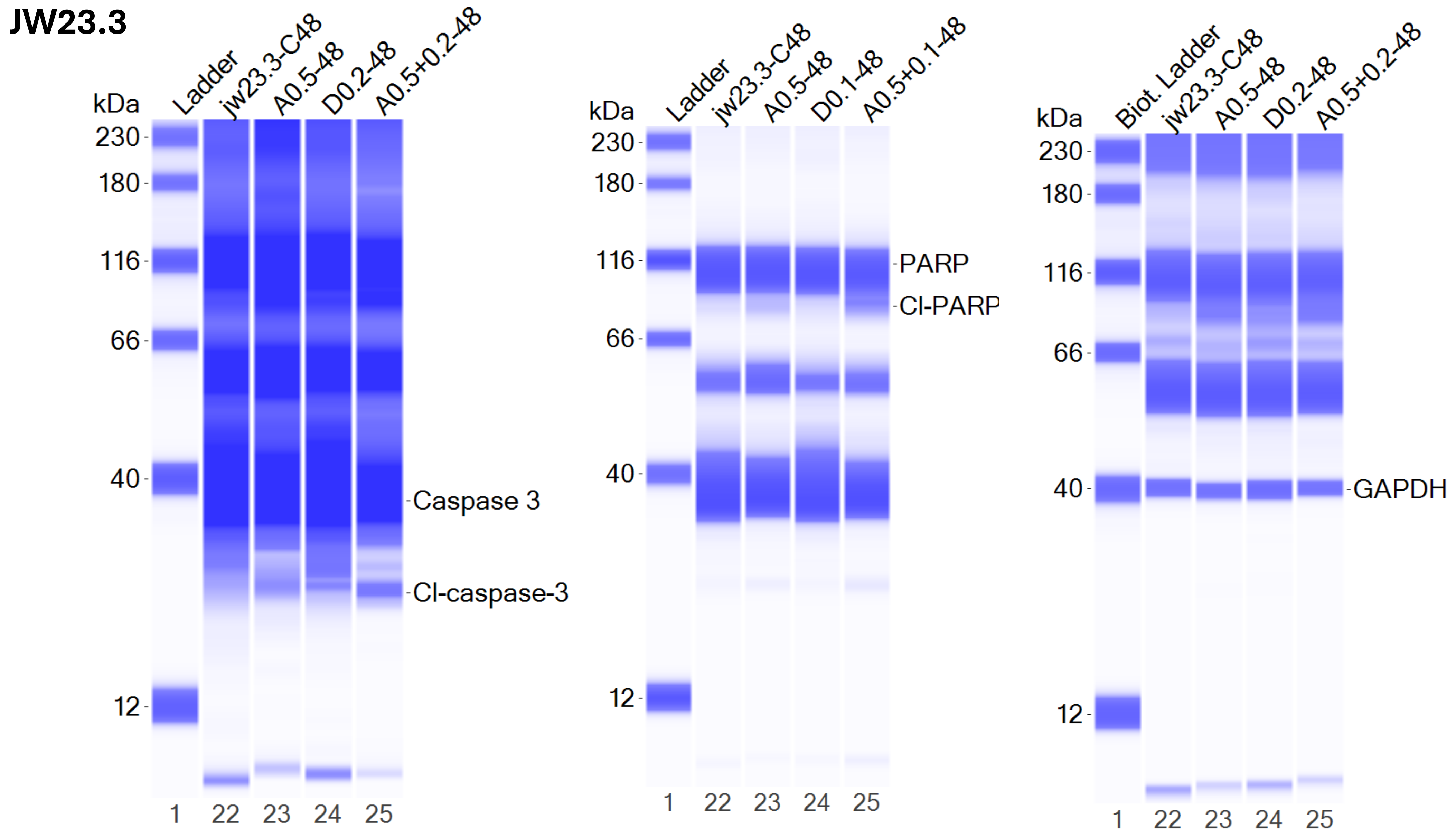

Supplementary Figure 7C

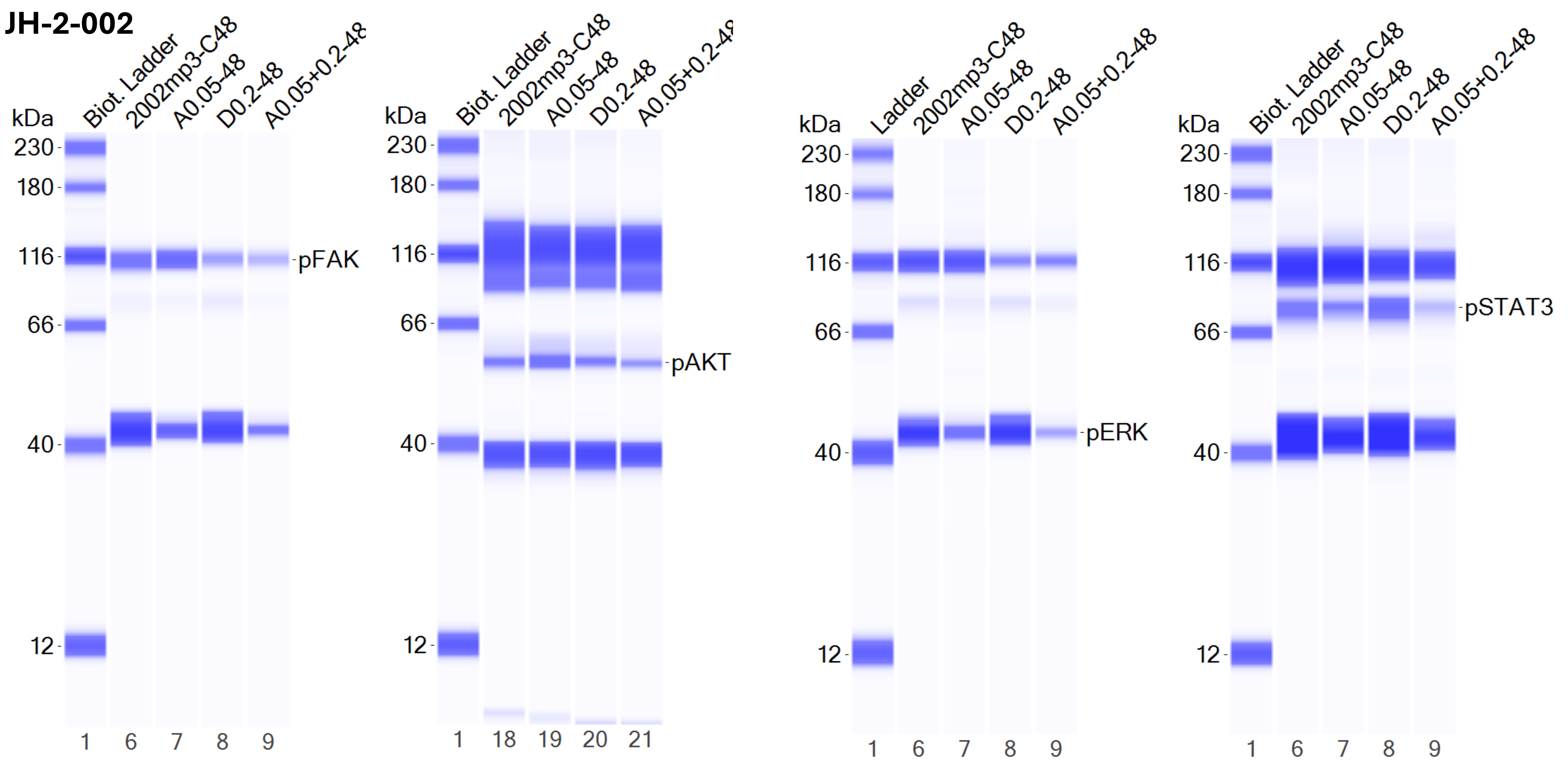

Supplementary Figure 7C

JH-2-002

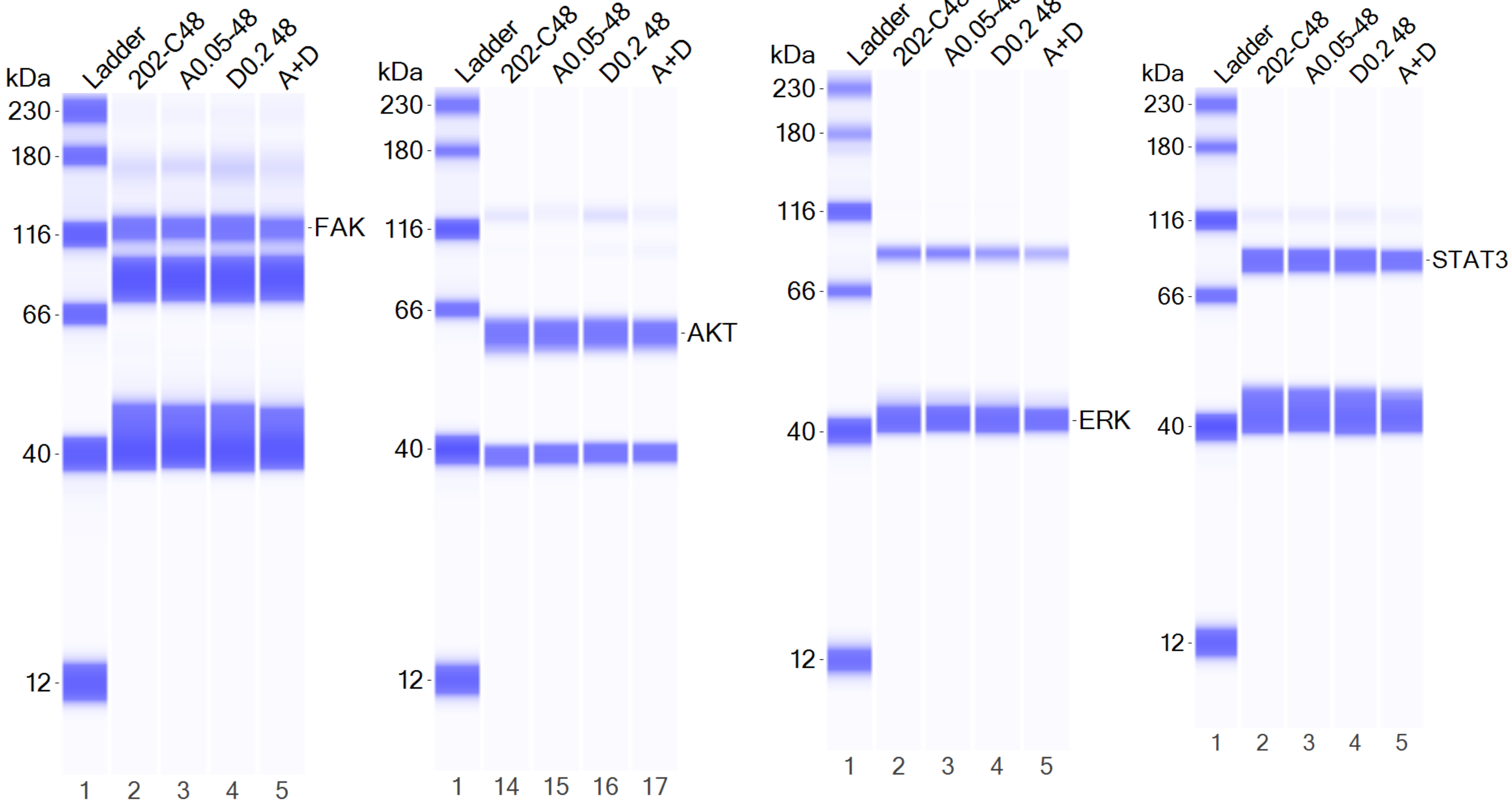

Supplementary Figure 7C

JH-2-002

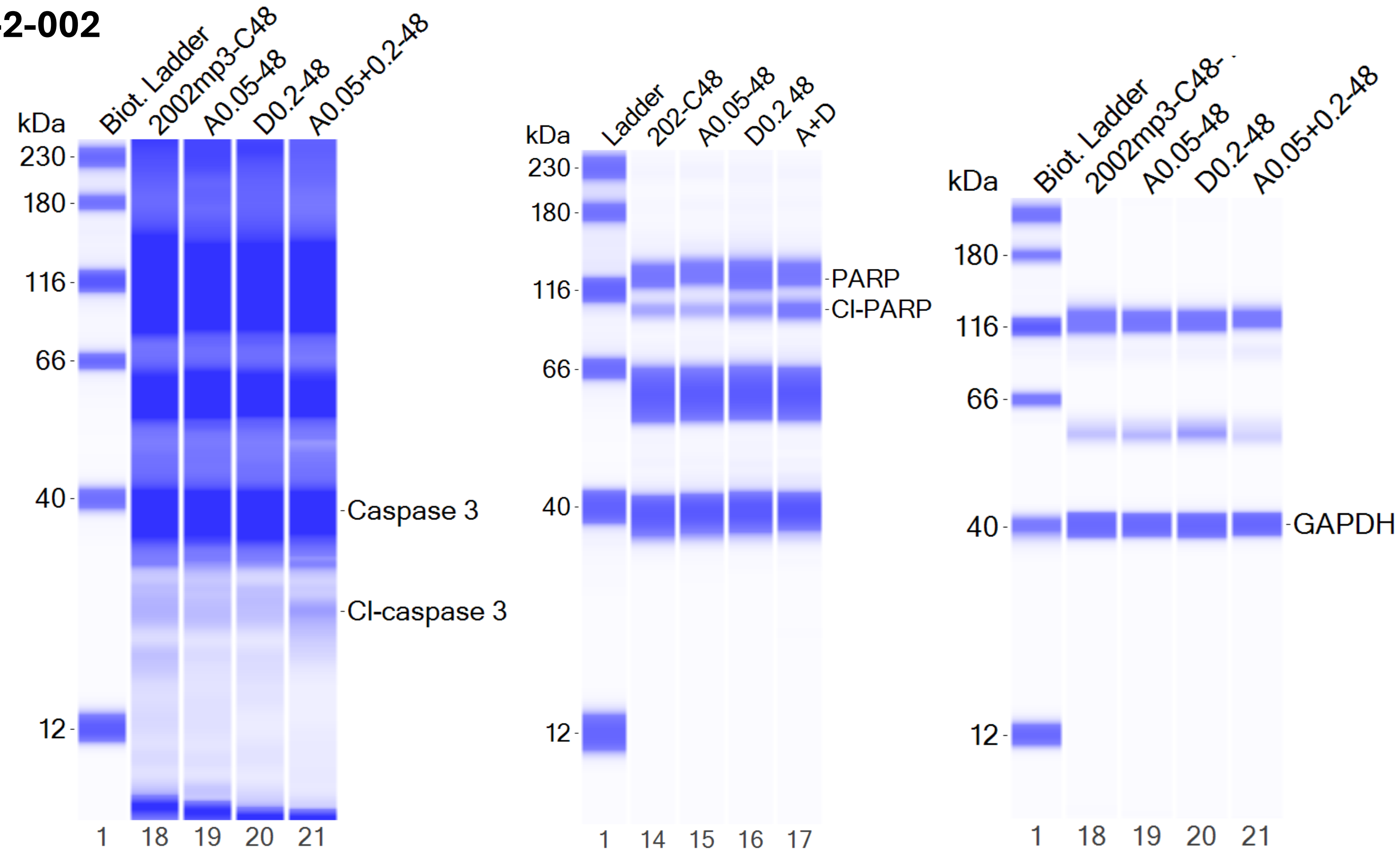

Supplementary Figure 7C

WU356

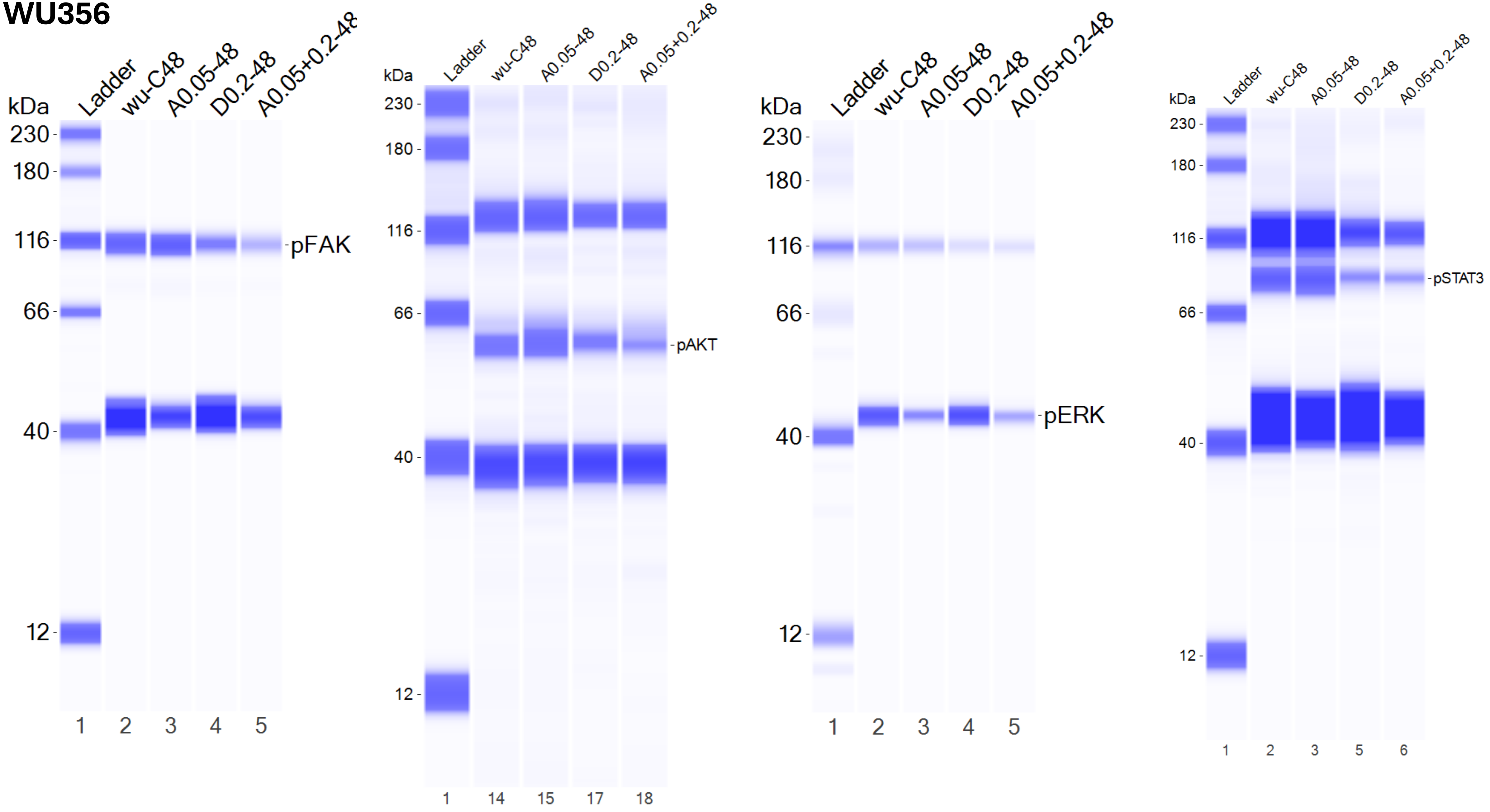

Supplementary Figure 7C

WU356

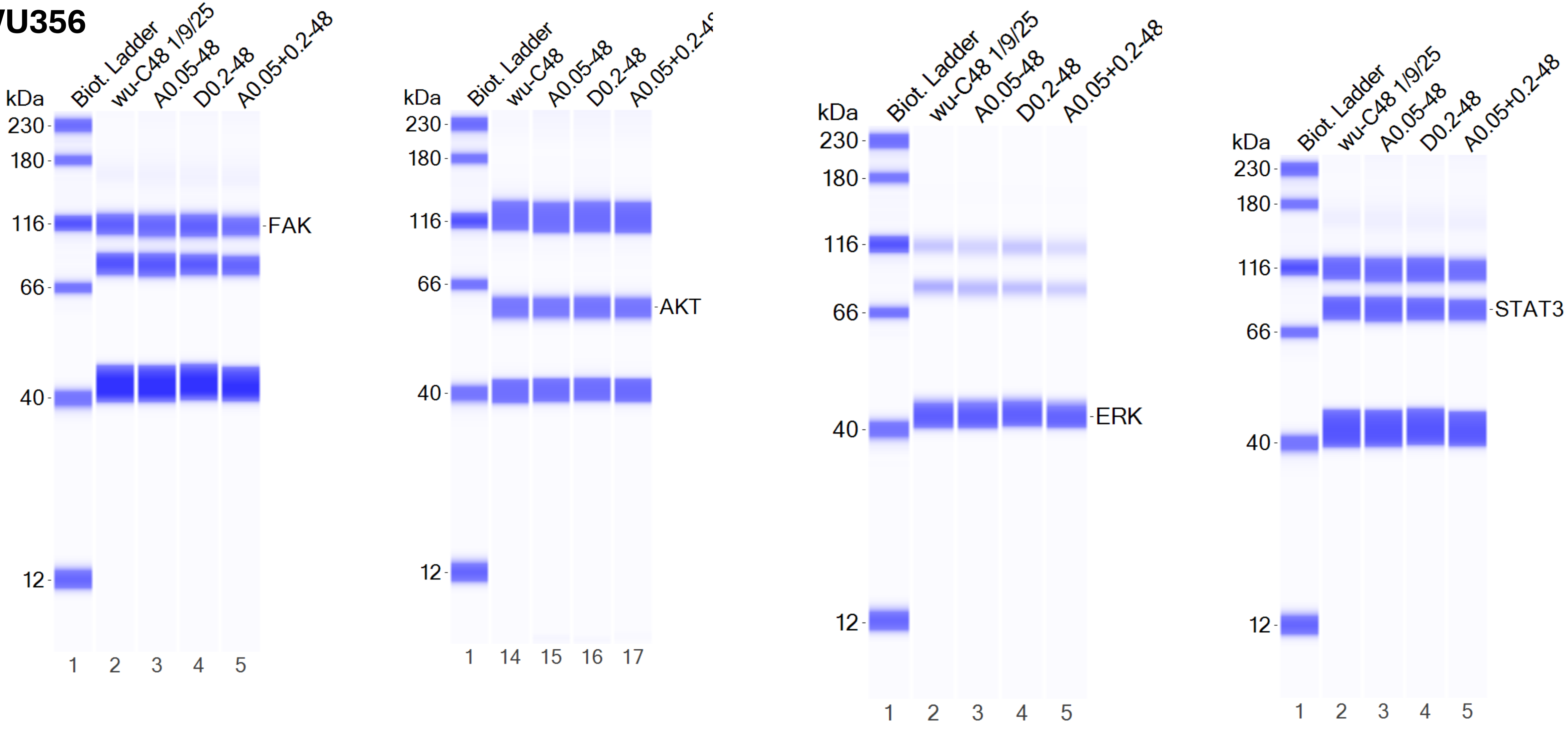

Supplementary Figure 7C

WU356

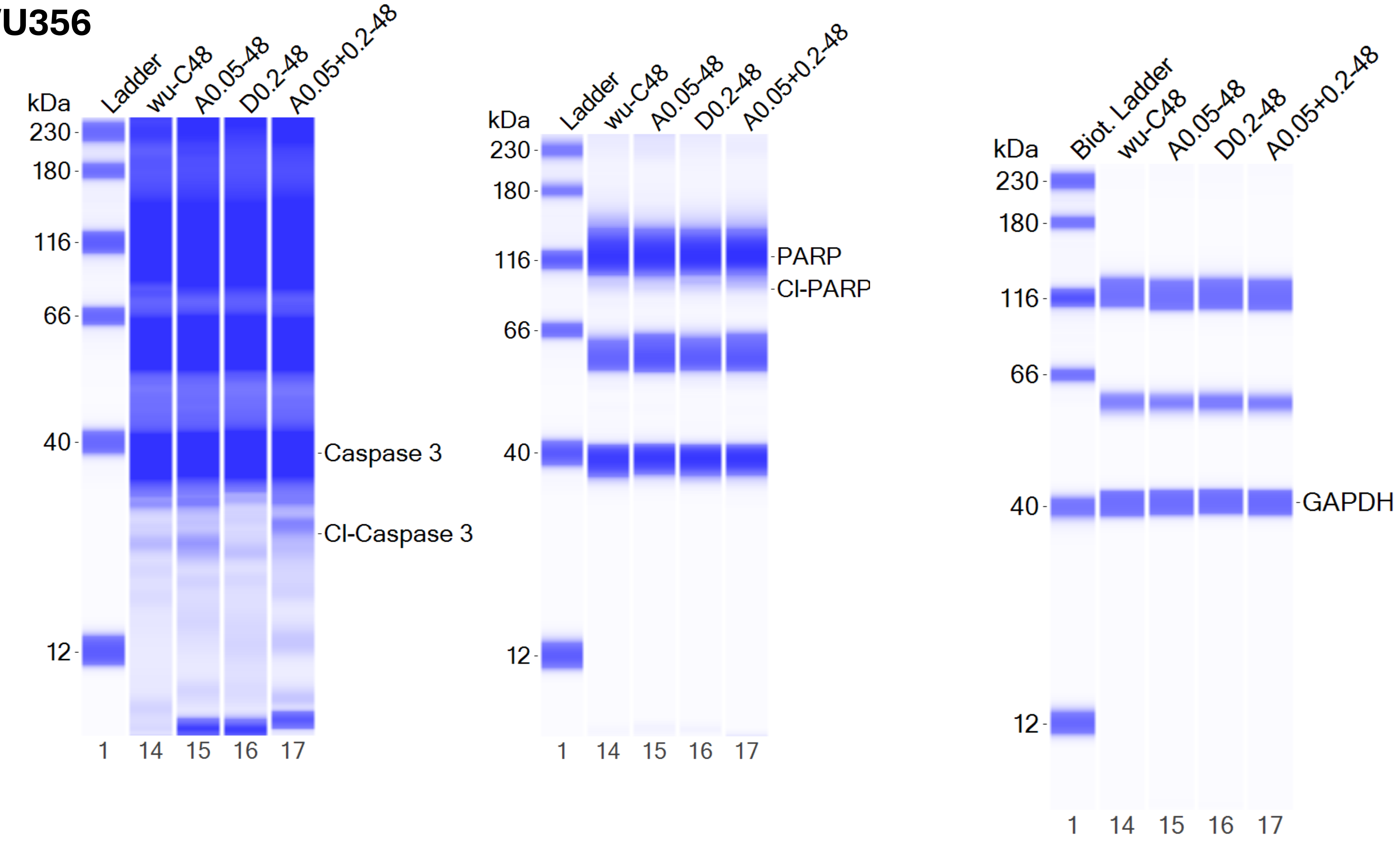

Supplementary Figure 8B

JH-2-002

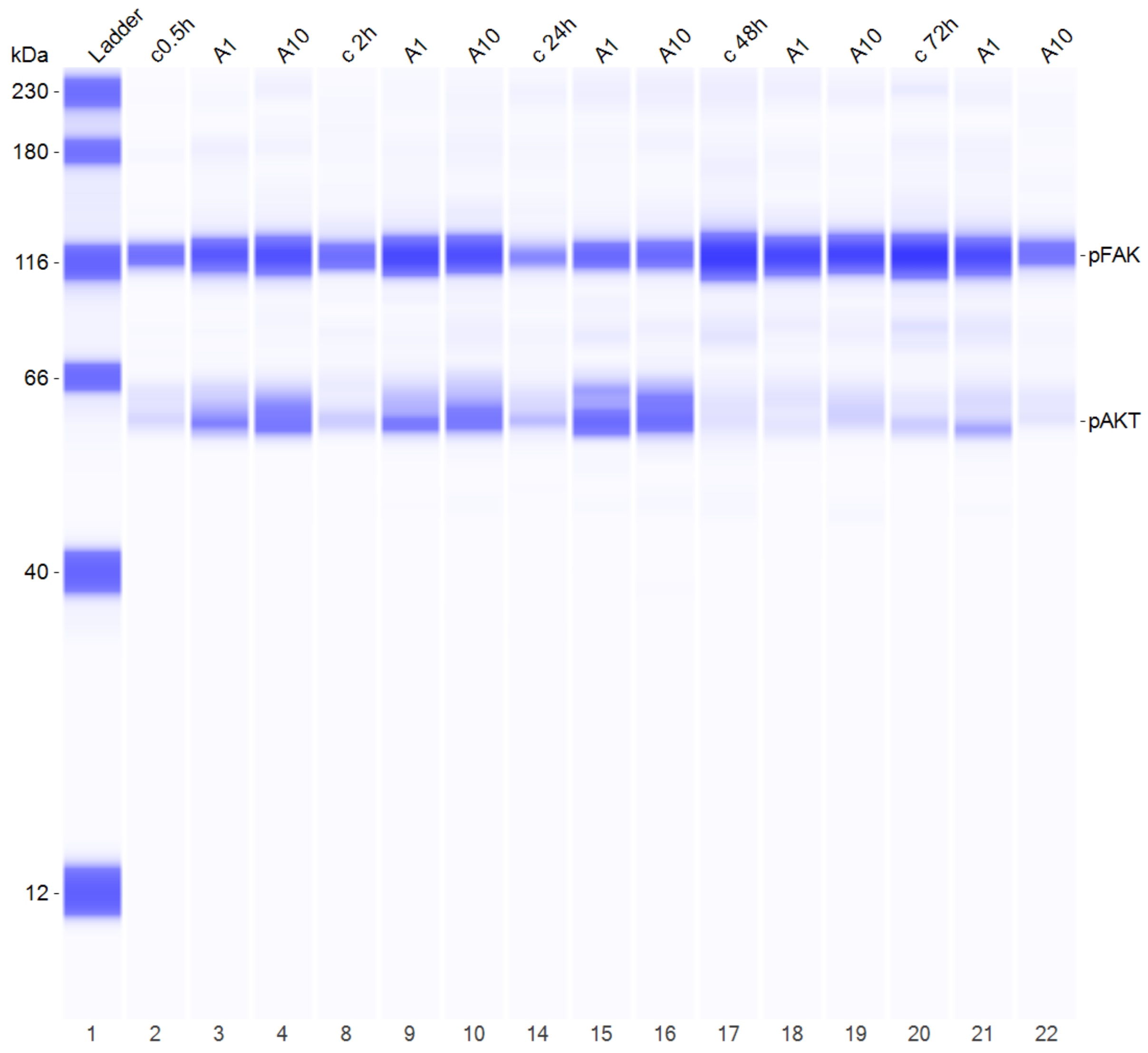

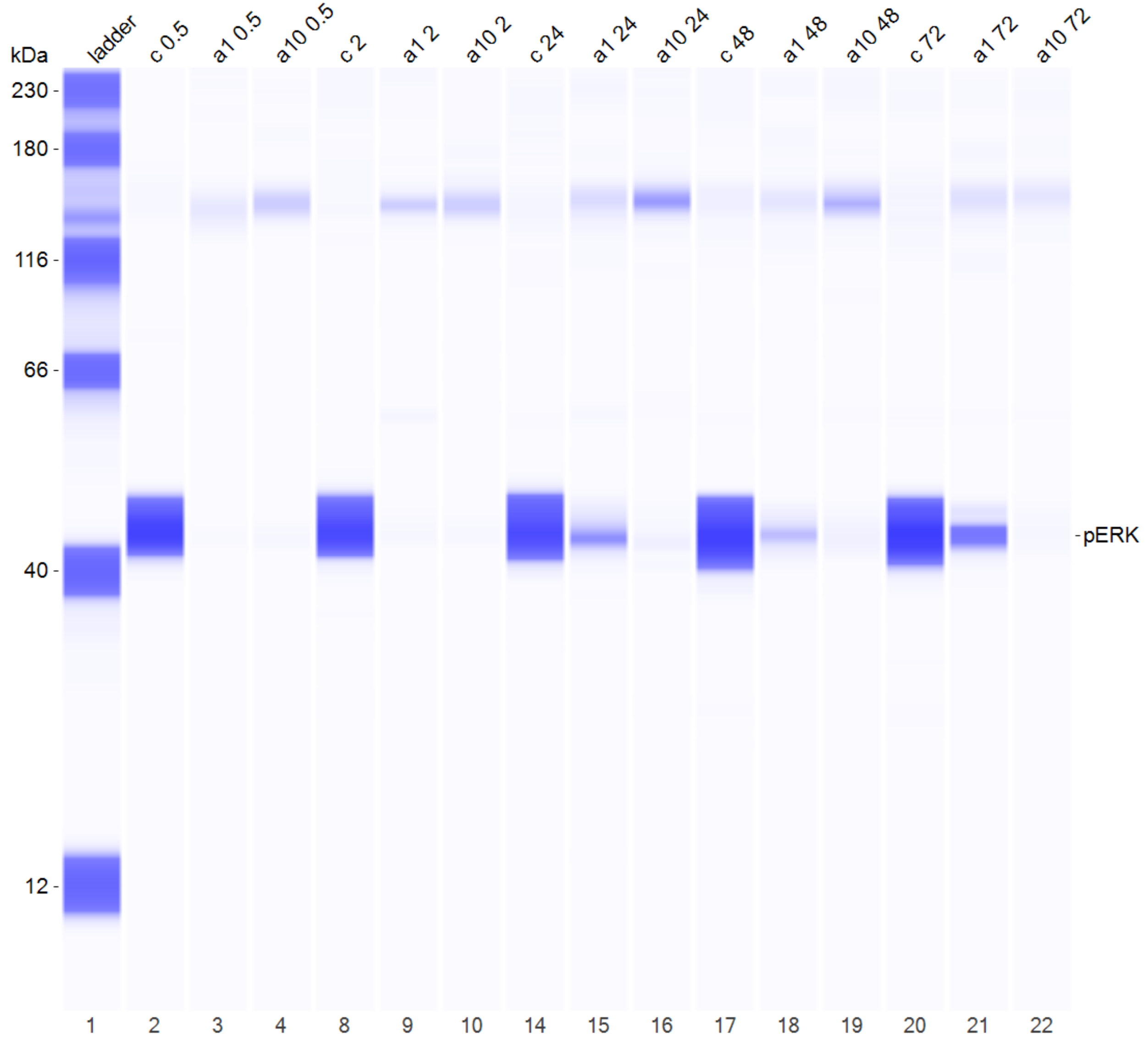

Supplementary Figure 8B

JH-2-002

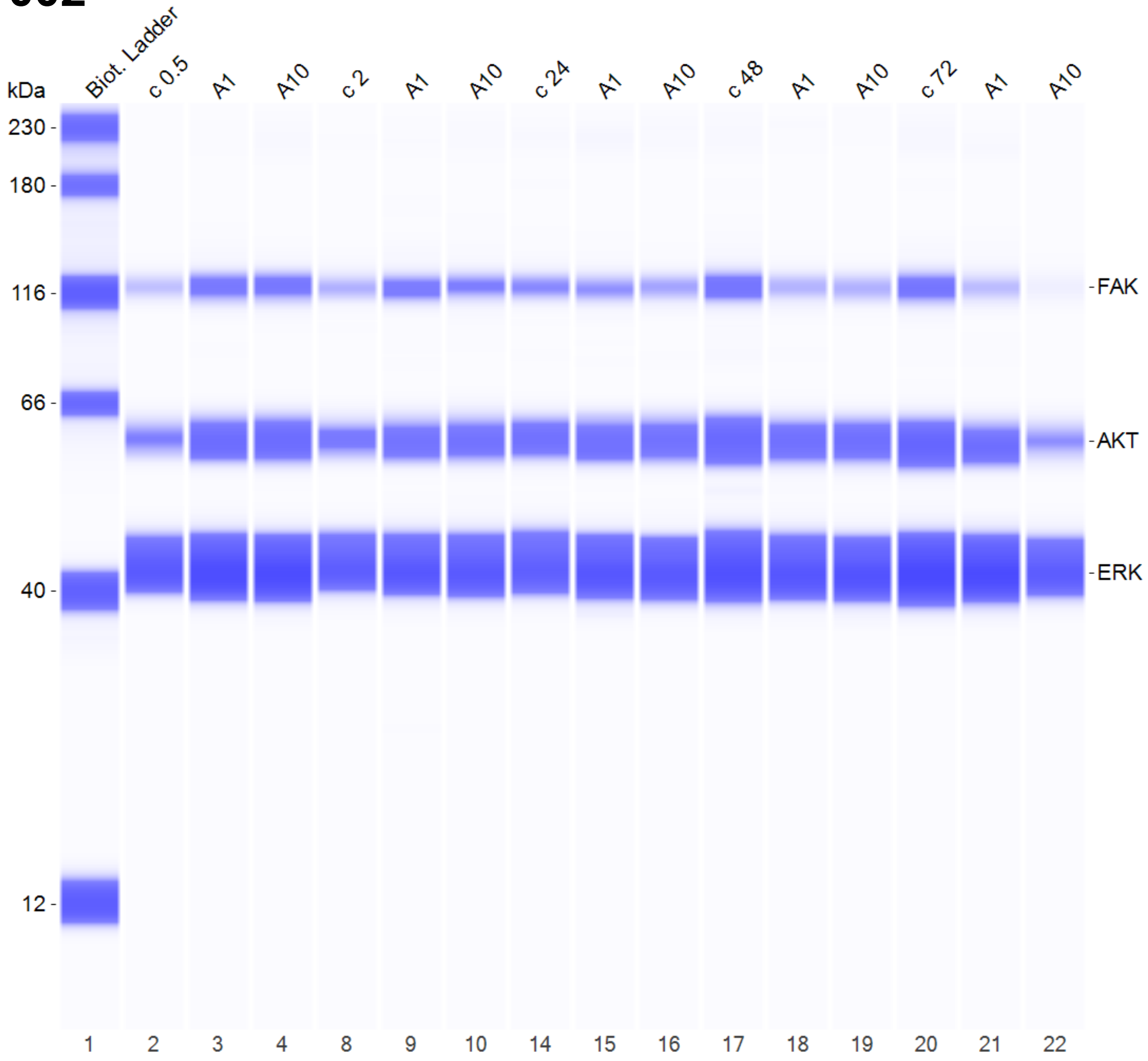

Supplementary Figure 12

MN2

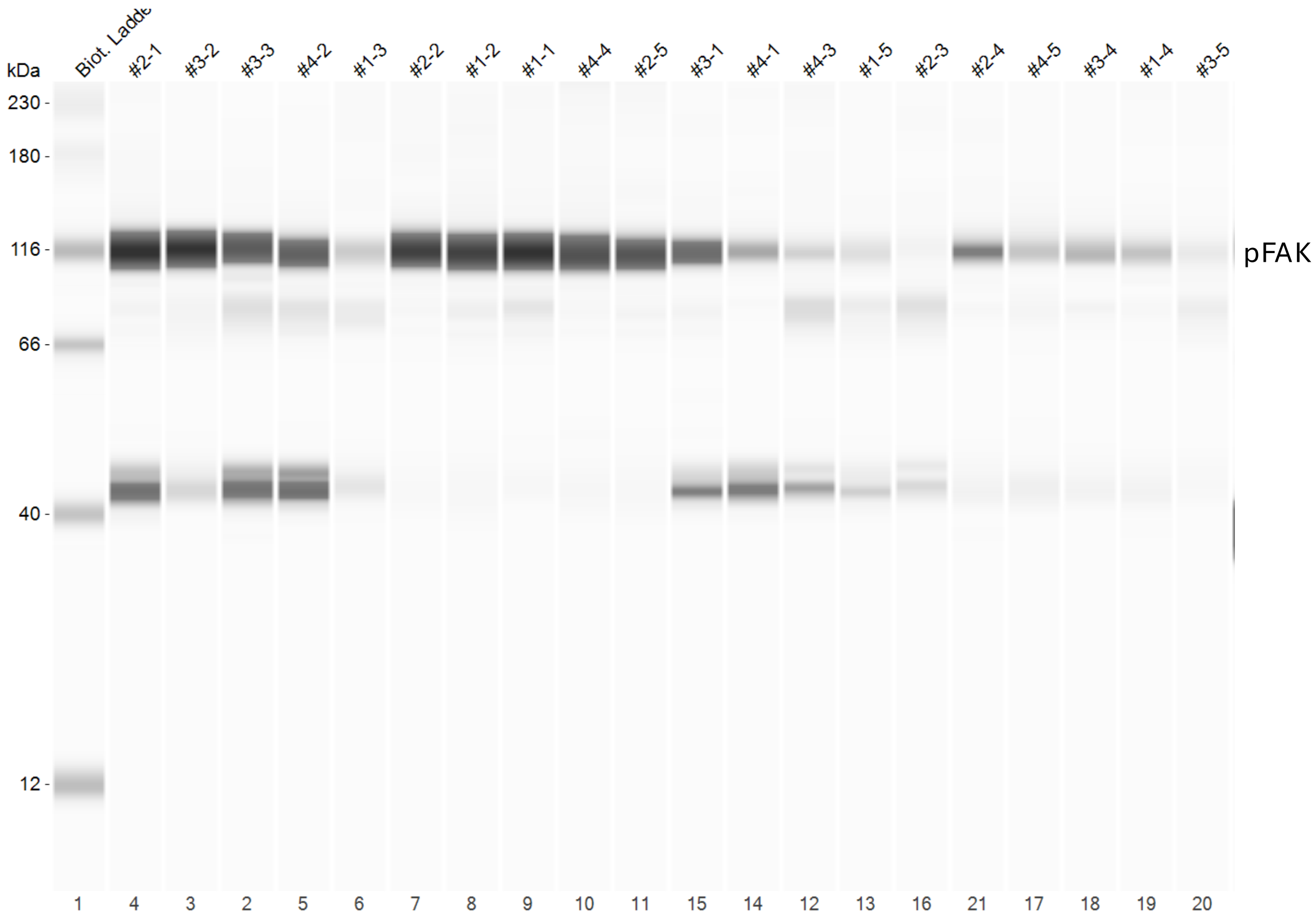

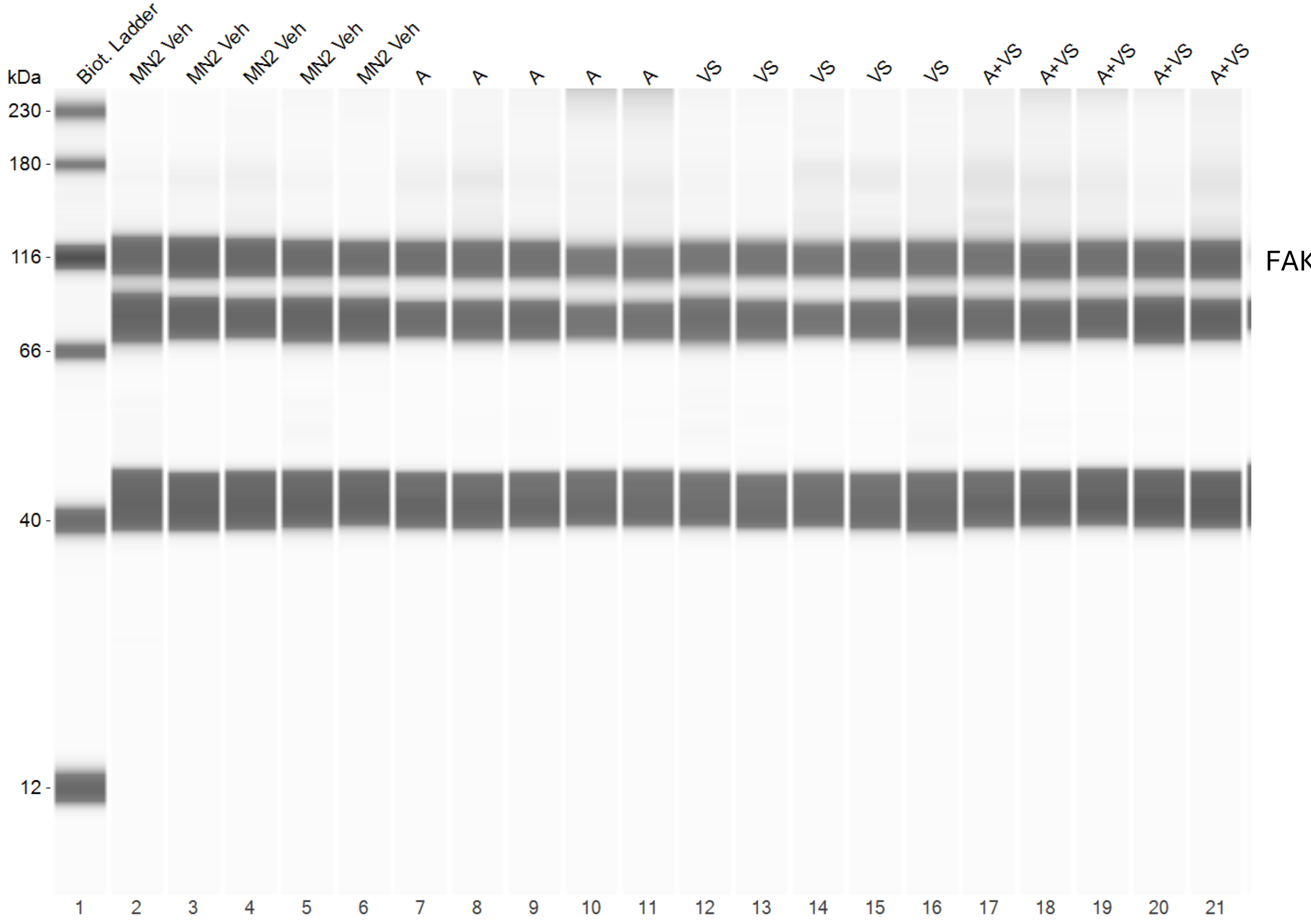

Supplementary Figure 12

MN2

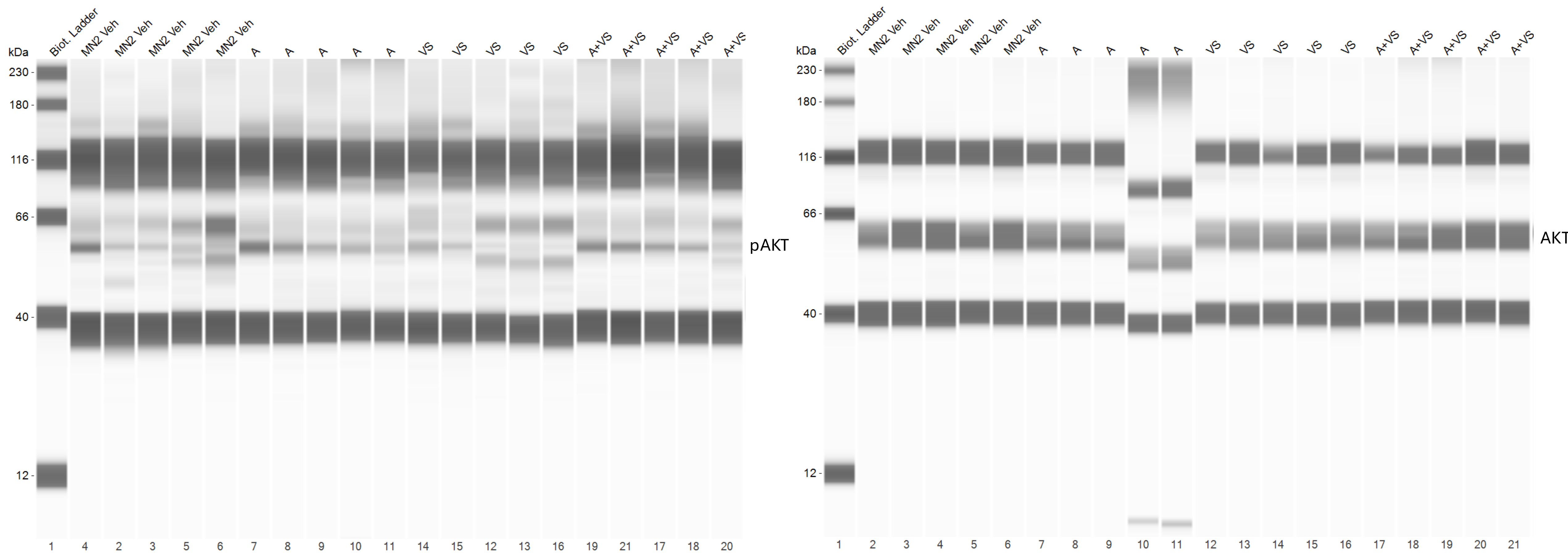

Supplementary Figure 12

MN2

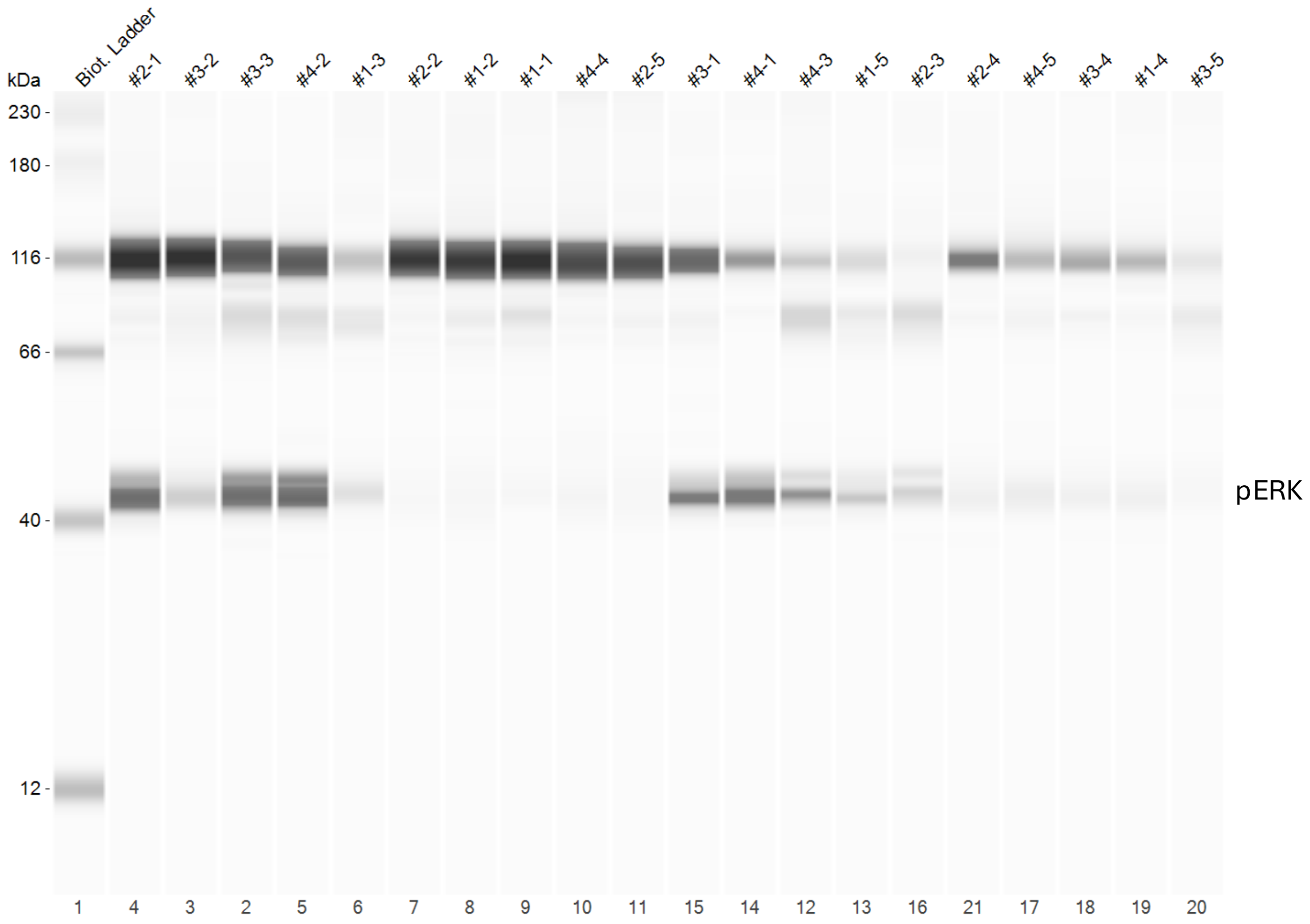

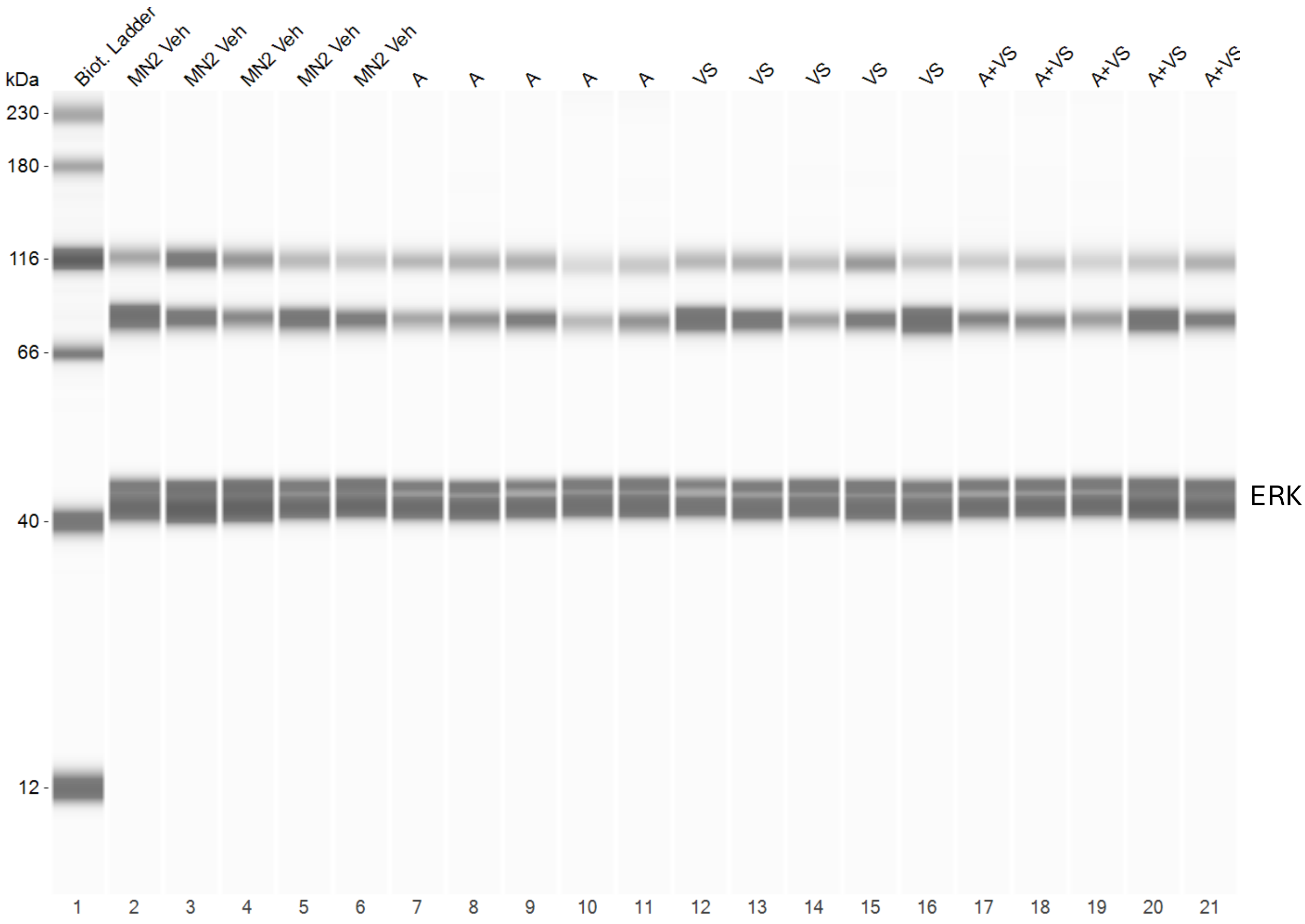

Supplementary Figure 12

MN2

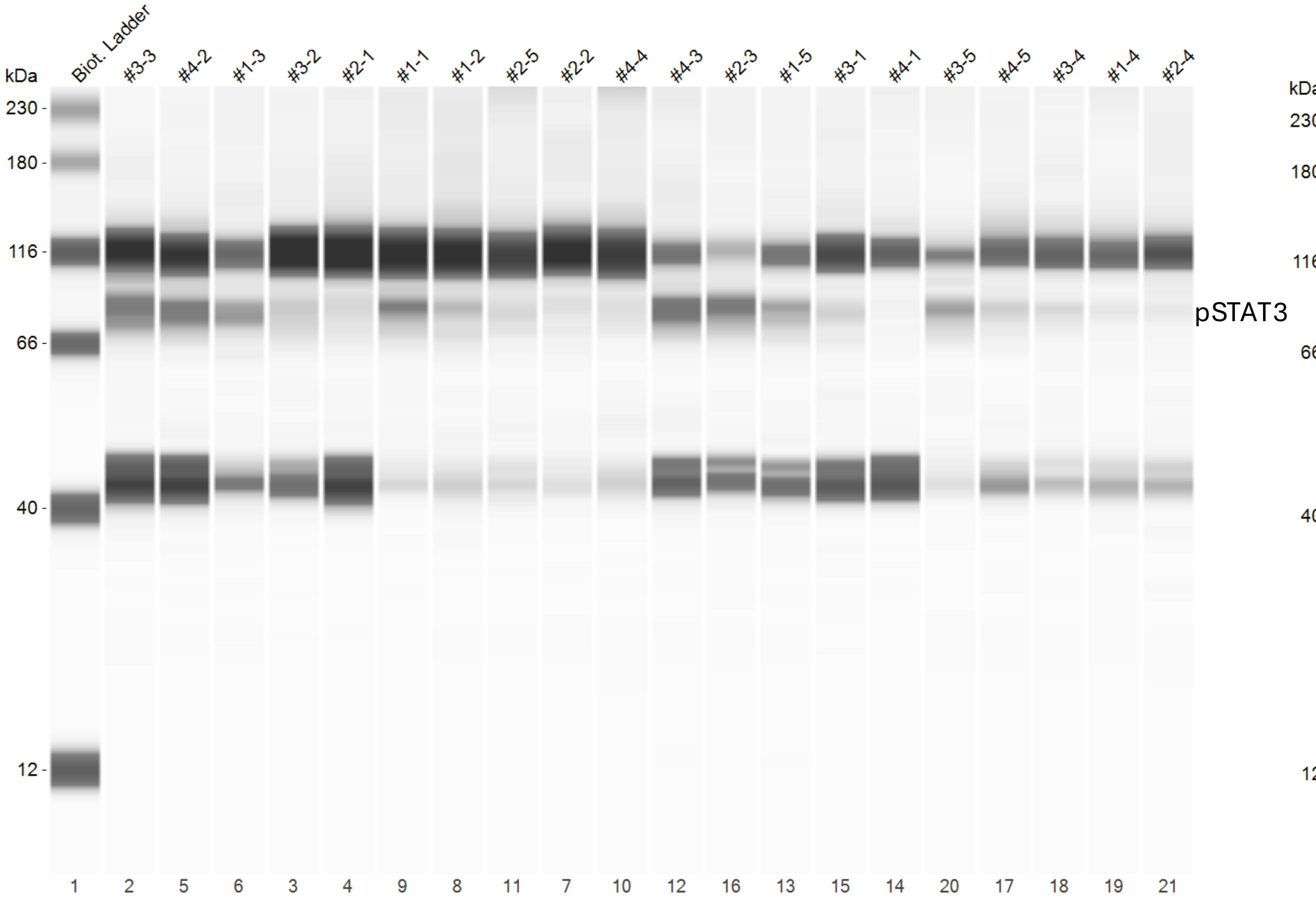

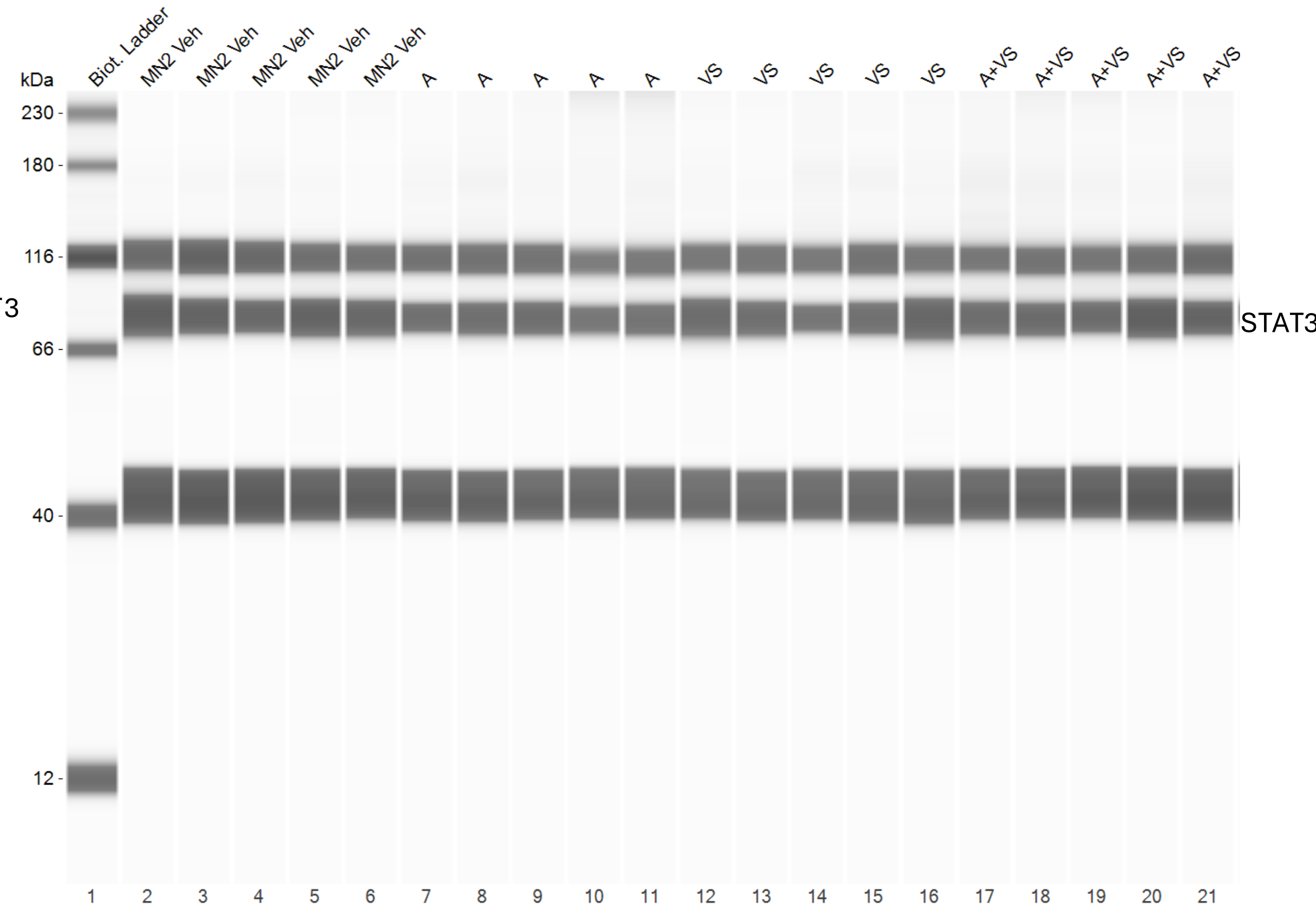

Supplementary Figure 12

MN2

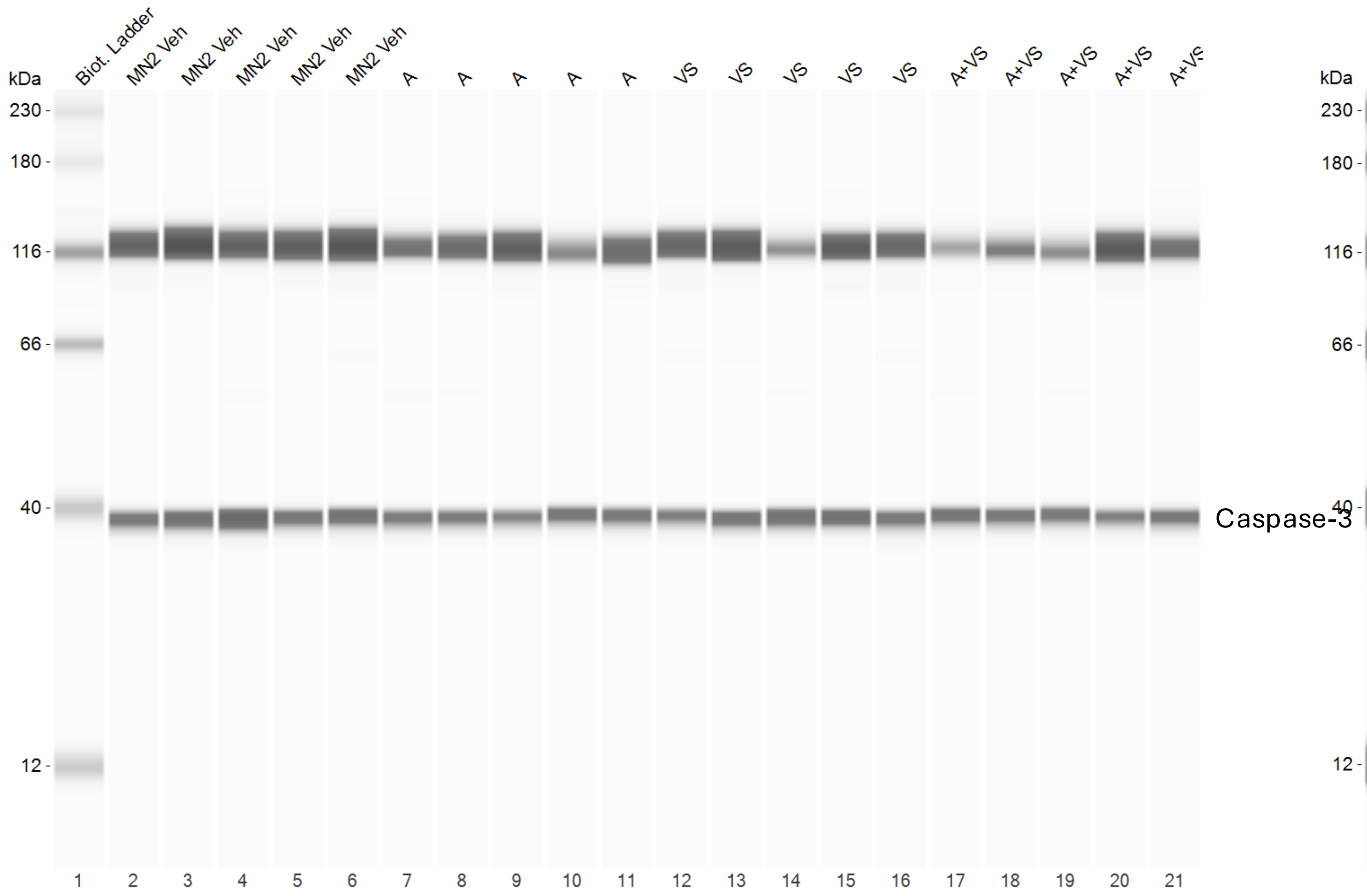

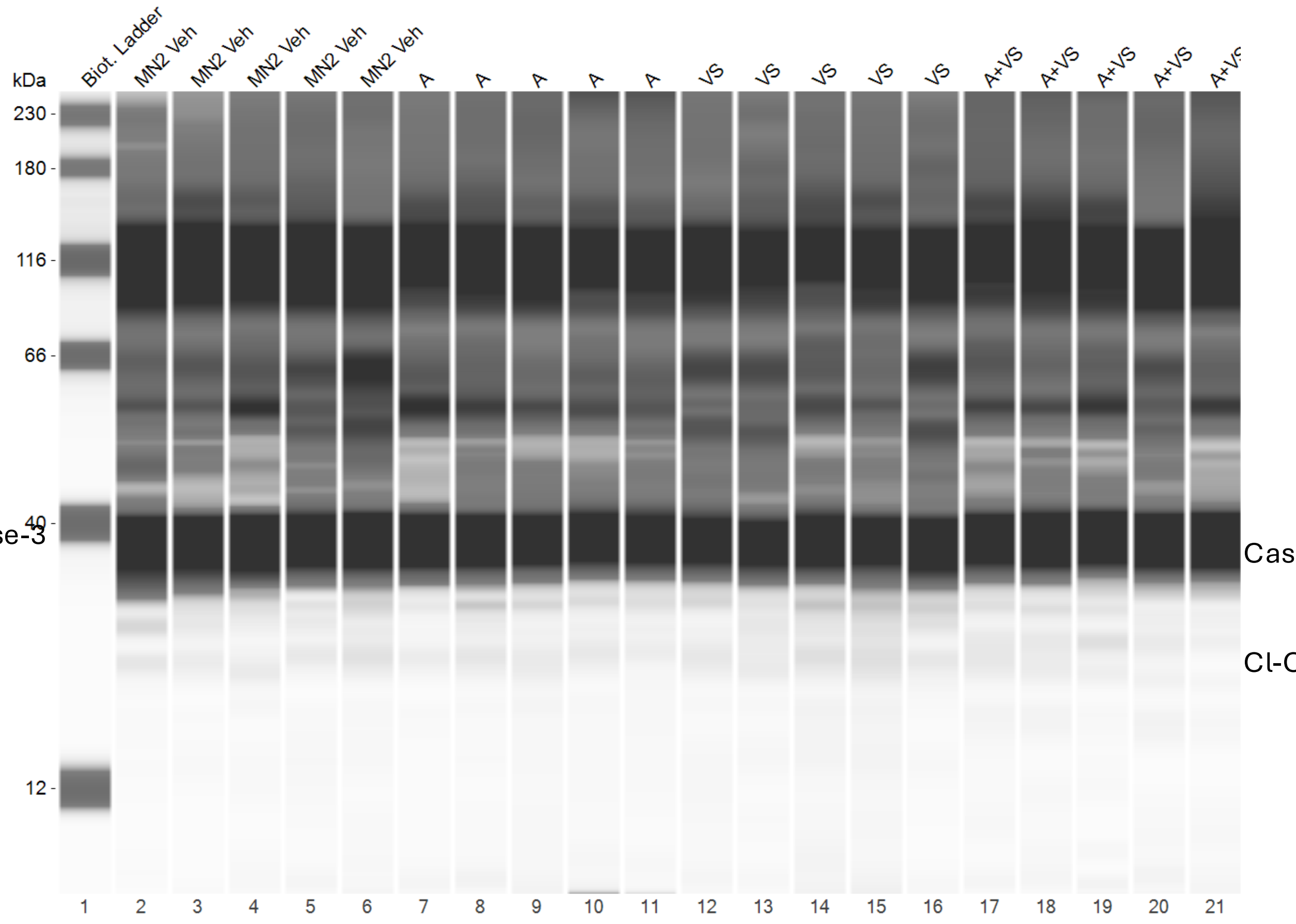

Supplementary Figure 12

MN2

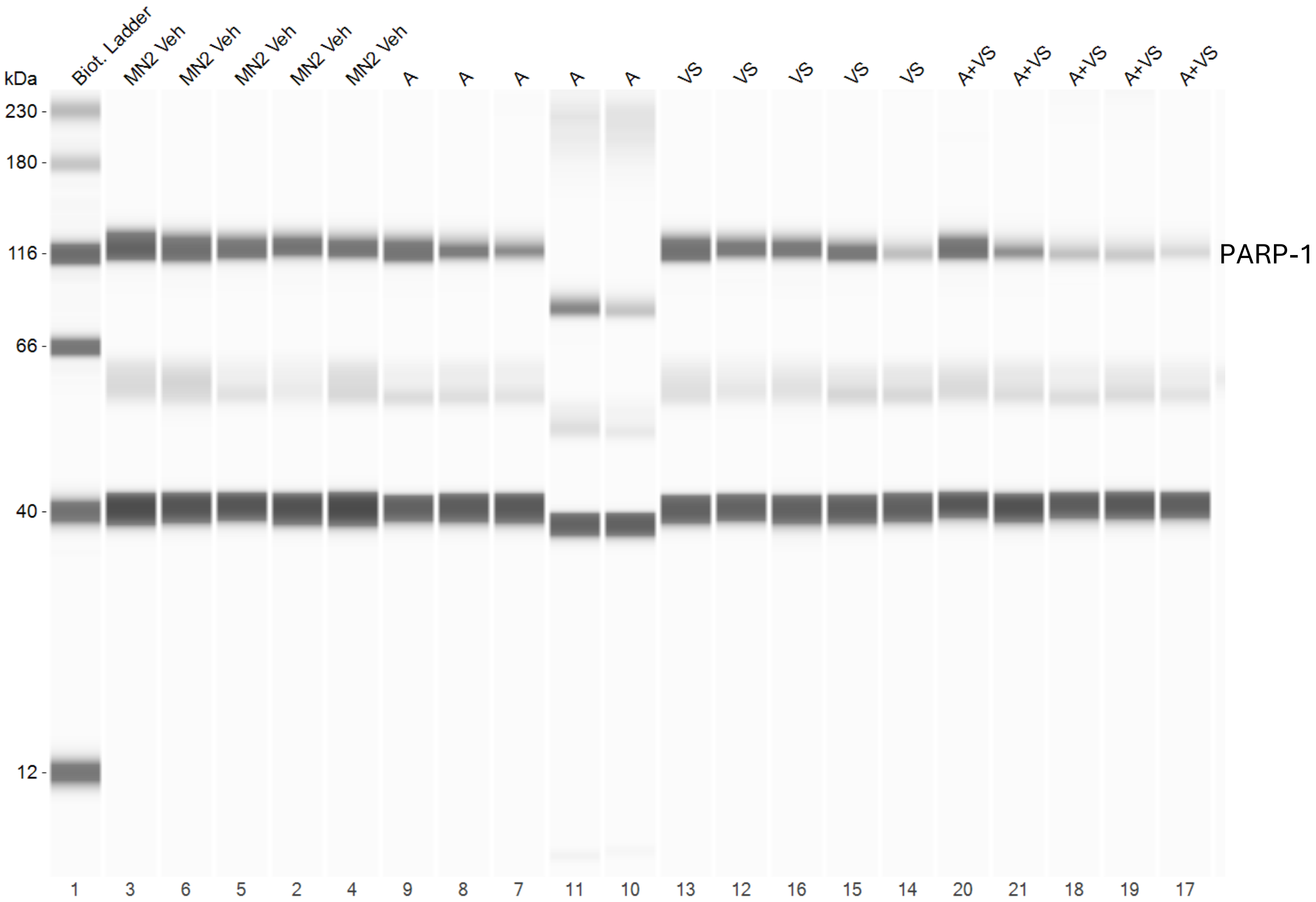

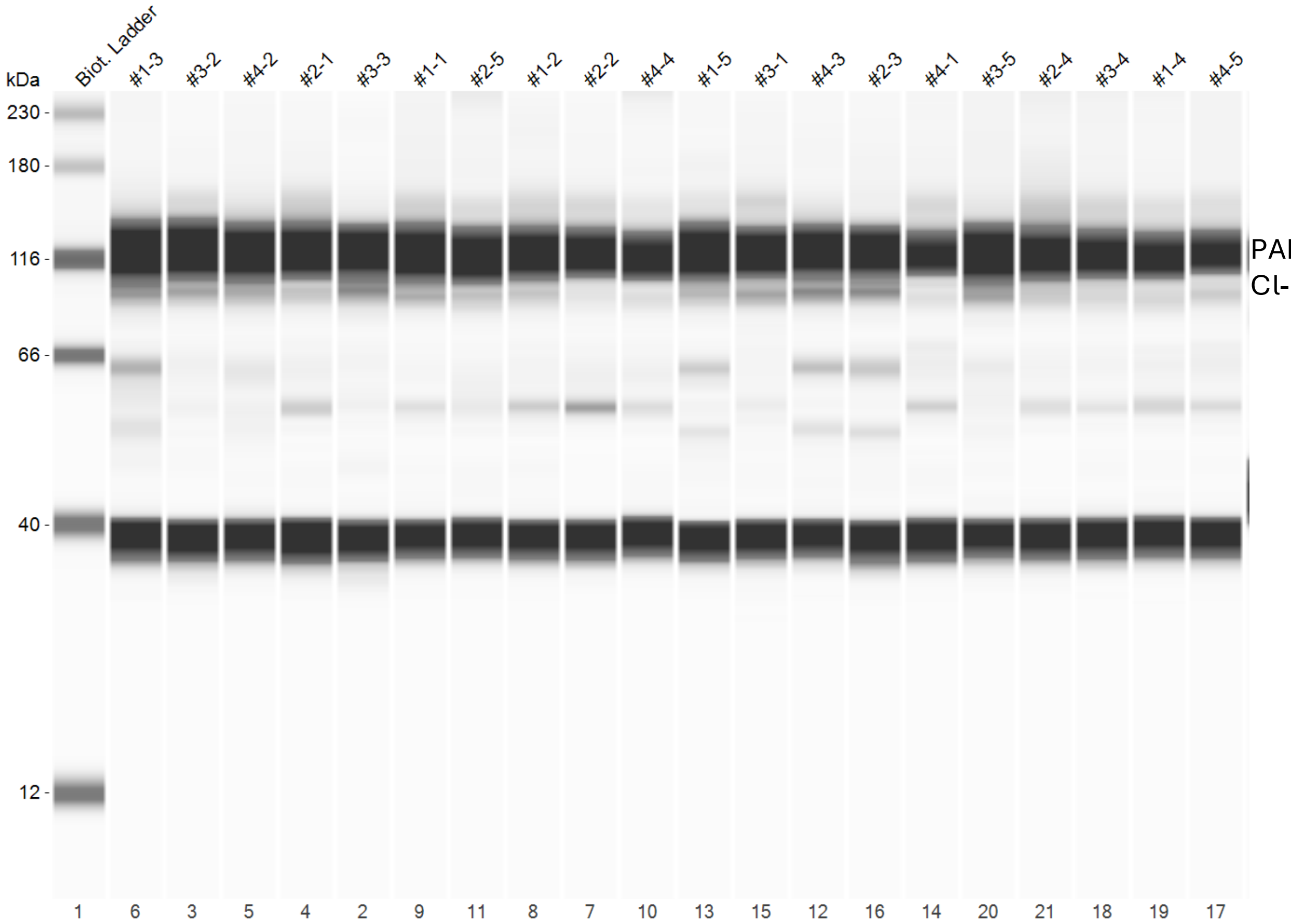

Supplementary Figure 12

MN2

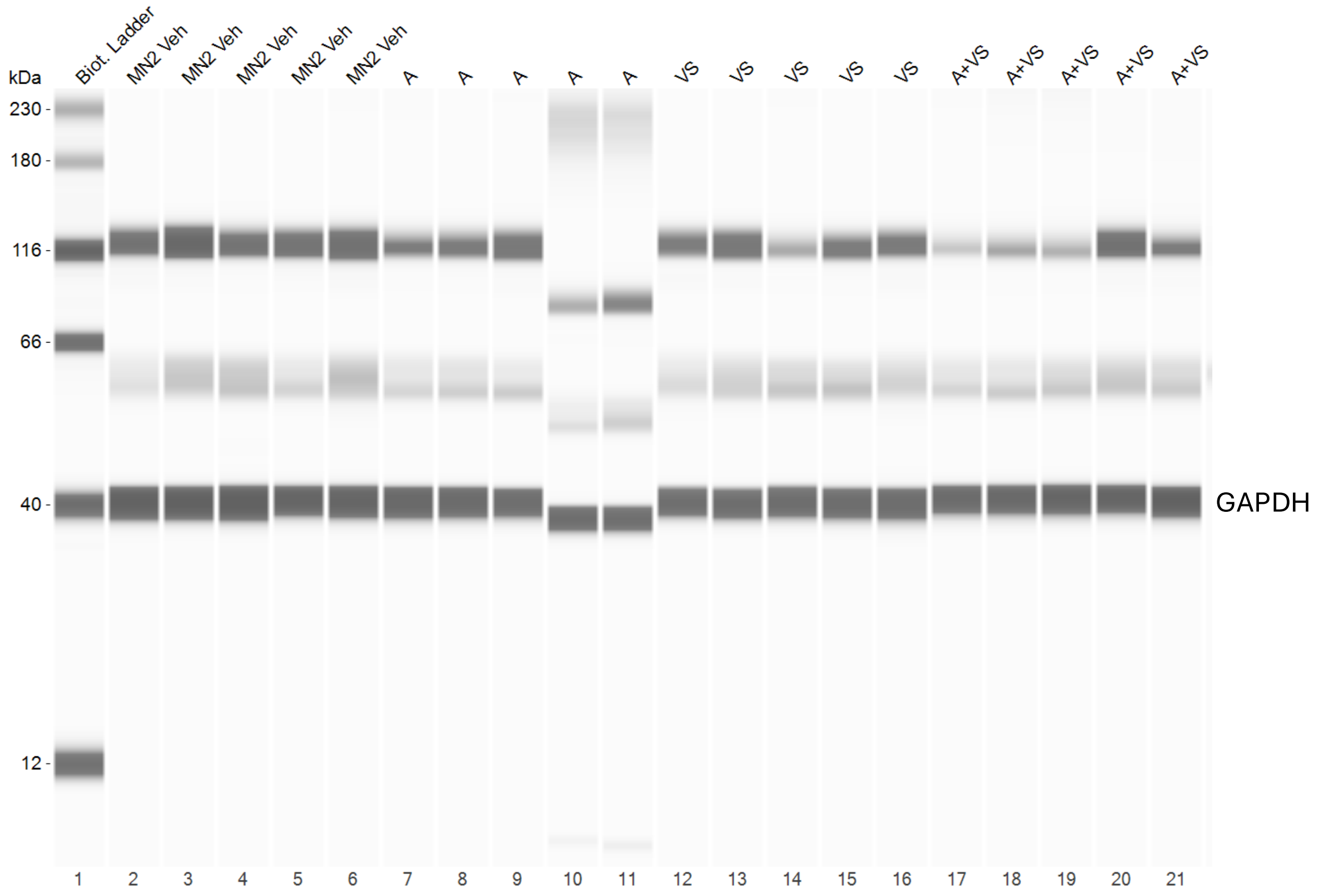

Supplementary Figure 13D
Raw blots

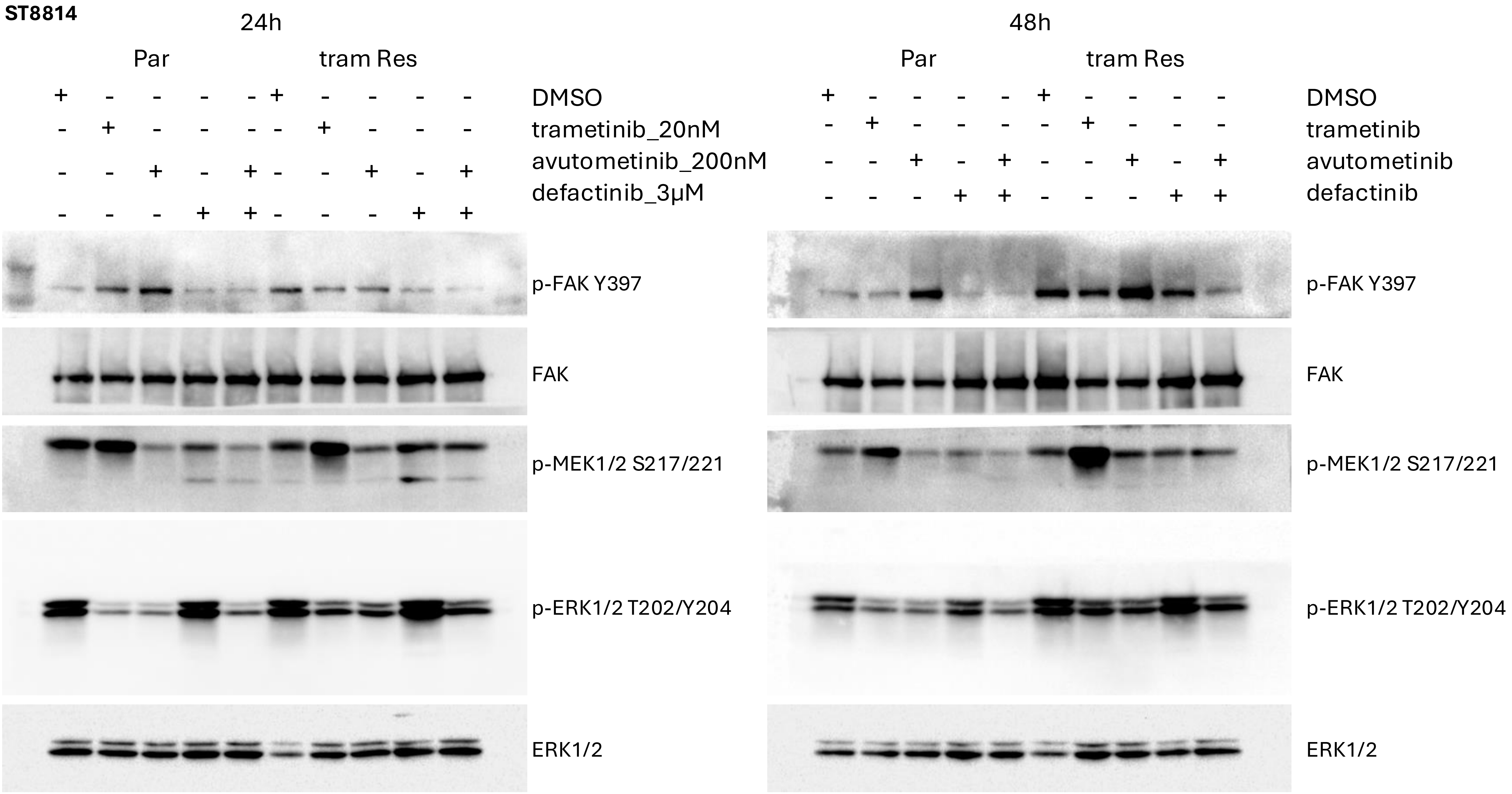

Supplementary Figure 13D

Raw blots

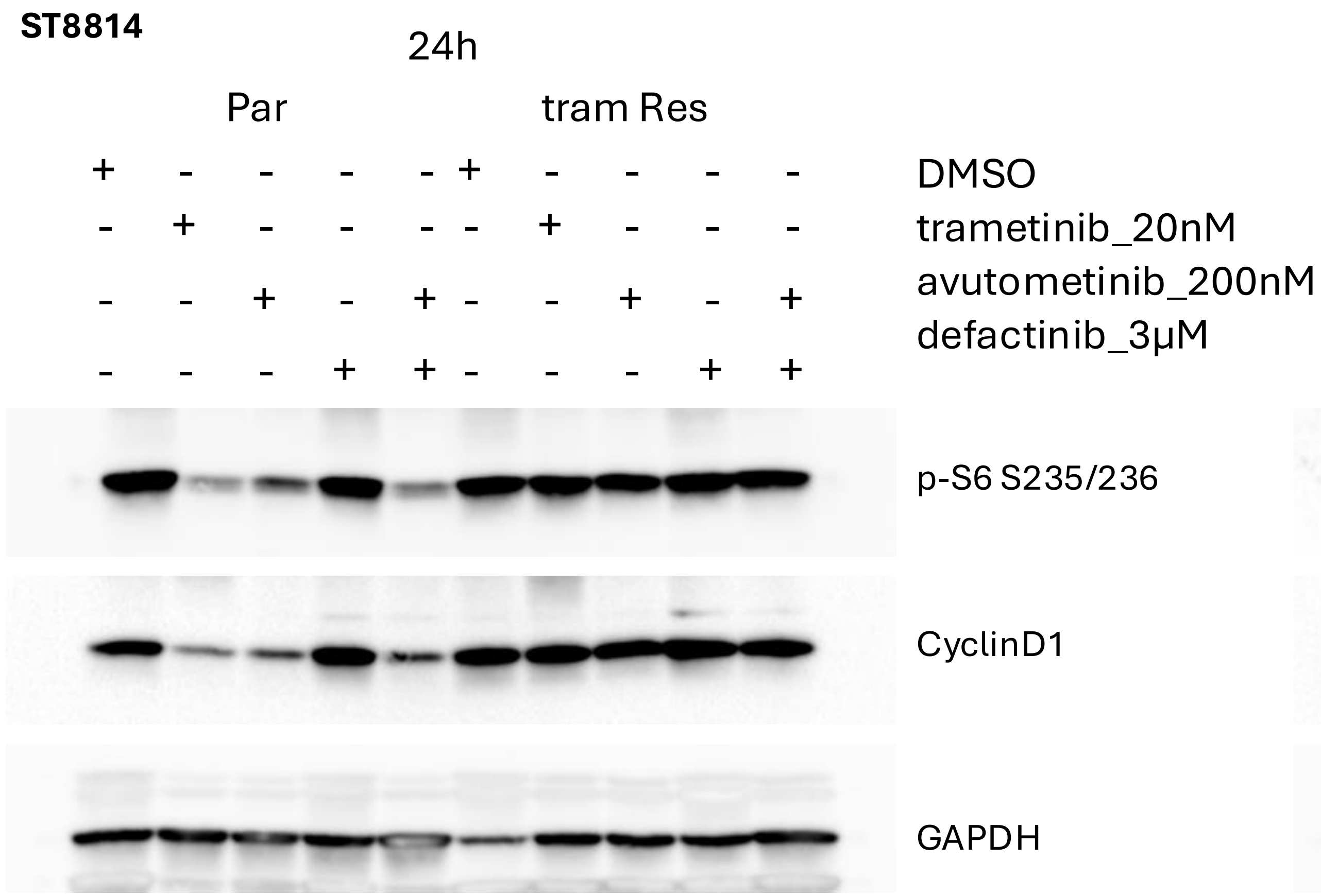

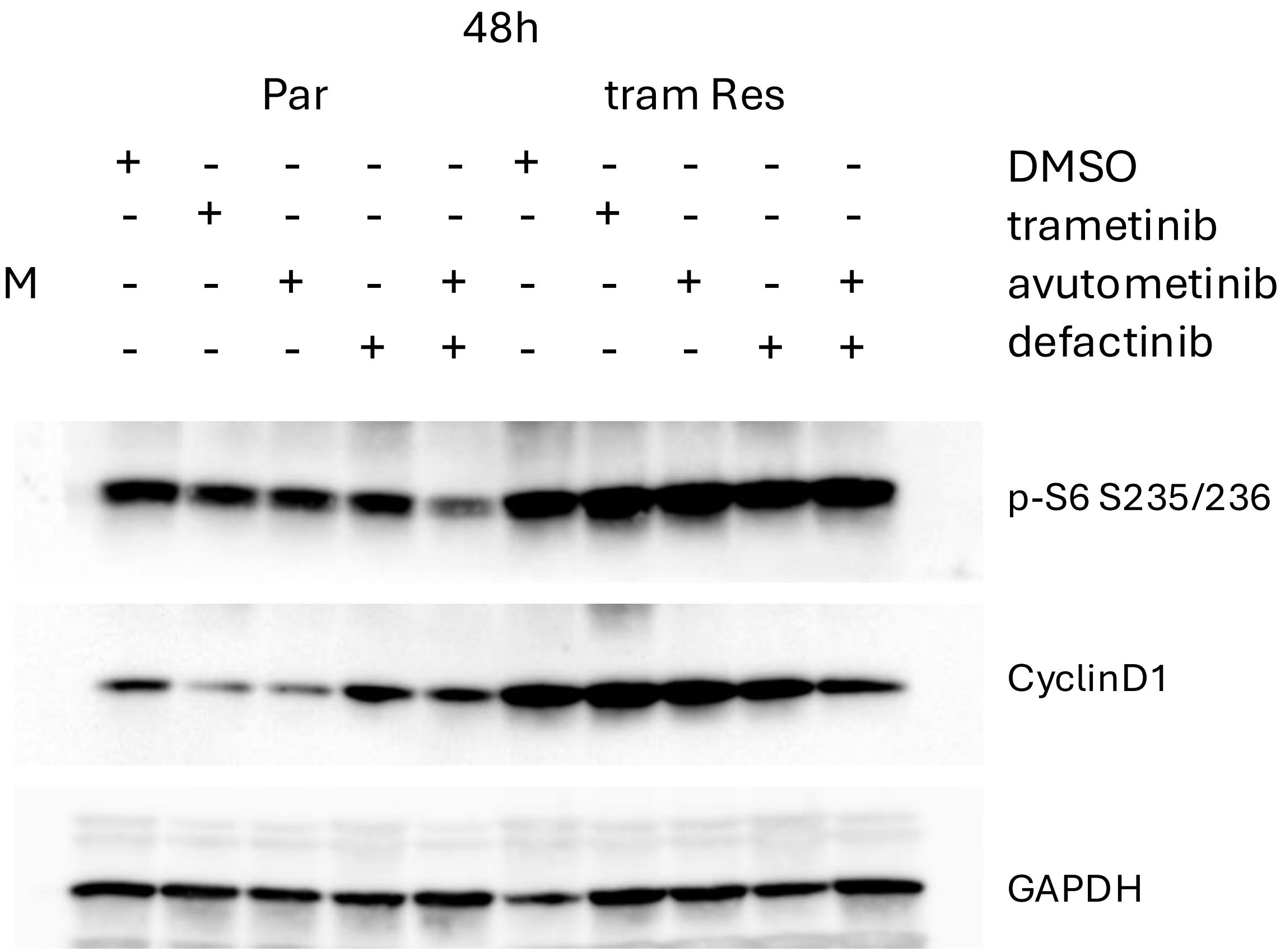

Supplementary Figure 13D

Raw blots

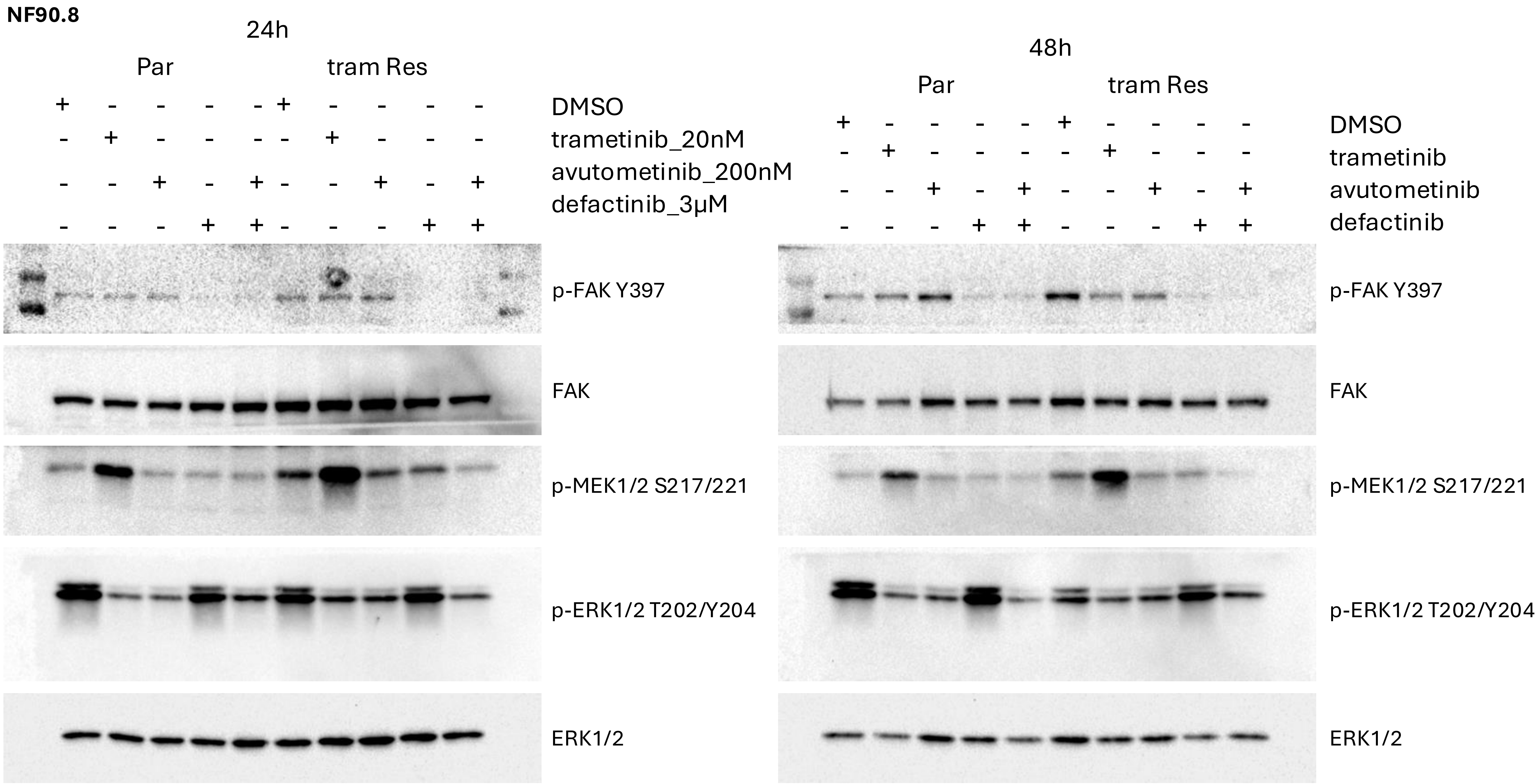

Supplementary Figure 13D
Raw blots

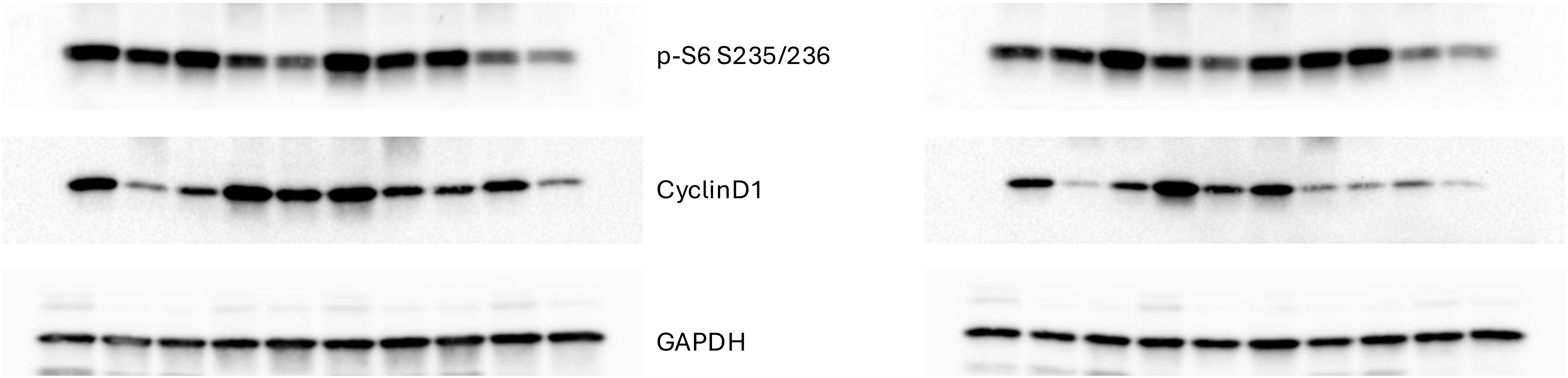

Supplement: Supplementary file 2 [file jci-136-201331-s008.pdf]
